# Fundamental Characterization, Photophysics and Photocatalysis of a Base Metal Iron(II)‐Cobalt(III) Dyad

**DOI:** 10.1002/chem.202100766

**Published:** 2021-06-01

**Authors:** Marina Huber‐Gedert, Michał Nowakowski, Ahmet Kertmen, Lukas Burkhardt, Natalia Lindner, Roland Schoch, Regine Herbst‐Irmer, Adam Neuba, Lennart Schmitz, Tae‐Kyu Choi, Jacek Kubicki, Wojciech Gawelda, Matthias Bauer

**Affiliations:** ^1^ Department Chemie Universität Paderborn Warburger Straße 100 33098 Paderborn Germany; ^2^ Faculty of Physics Adam Mickiewicz University Poznań ul. Uniwersytetu Poznańskiego 2 Poznań 61-614 Poland; ^3^ Institut für Anorganische Chemie Universität Göttingen Tammannstraße 4 37077 Göttingen Germany; ^4^ European XFEL Holzkoppel 4 22869 Schenefeld Germany; ^5^ Department of Chemistry Universidad Autónoma de Madrid Campus Universitario 28049 Madrid Spain; ^6^ IMDEA-Nanociencia Calle Faraday 9 28049 Madrid Spain

**Keywords:** base metal complexes, dyads, photocatalysis, proton reduction, time-resolved spectroscopy

## Abstract

A new base metal iron‐cobalt dyad has been obtained by connection between a heteroleptic tetra‐NHC iron(II) photosensitizer combining a 2,6‐bis[3‐(2,6‐diisopropylphenyl)imidazol‐2‐ylidene]pyridine with 2,6‐bis(3‐methyl‐imidazol‐2‐ylidene)‐4,4′‐bipyridine ligand, and a cobaloxime catalyst. This novel iron(II)‐cobalt(III) assembly has been extensively characterized by ground‐ and excited‐state methods like X‐ray crystallography, X‐ray absorption spectroscopy, (spectro‐)electrochemistry, and steady‐state and time‐resolved optical absorption spectroscopy, with a particular focus on the stability of the molecular assembly in solution and determination of the excited‐state landscape. NMR and UV/Vis spectroscopy reveal dissociation of the dyad in acetonitrile at concentrations below 1 mM and high photostability. Transient absorption spectroscopy after excitation into the metal‐to‐ligand charge transfer absorption band suggests a relaxation cascade originating from hot singlet and triplet MLCT states, leading to the population of the ^3^MLCT state that exhibits the longest lifetime. Finally, decay into the ground state involves a ^3^MC state. Attachment of cobaloxime to the iron photosensitizer increases the ^3^MLCT lifetime at the iron centre. Together with the directing effect of the linker, this potentially makes the dyad more active in photocatalytic proton reduction experiments than the analogous two‐component system, consisting of the iron photosensitizer and Co(dmgH)_2_(py)Cl. This work thus sheds new light on the functionality of base metal dyads, which are important for more efficient and sustainable future proton reduction systems.

## Graphical abstract

**Keeping it simple**: A new dyad that is active in photocatalytic proton reduction employing only base metals has been achieved. It connects a tetra‐NHC‐Fe^II^ photosensitizer with a Co^III^ cobaloxime catalyst. The photocatalytic activity is explained by comparison to the equivalent two‐component system and thorough ground‐ and excited‐state characterization.

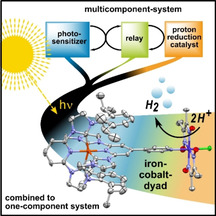

## Introduction

**Figure 1:**
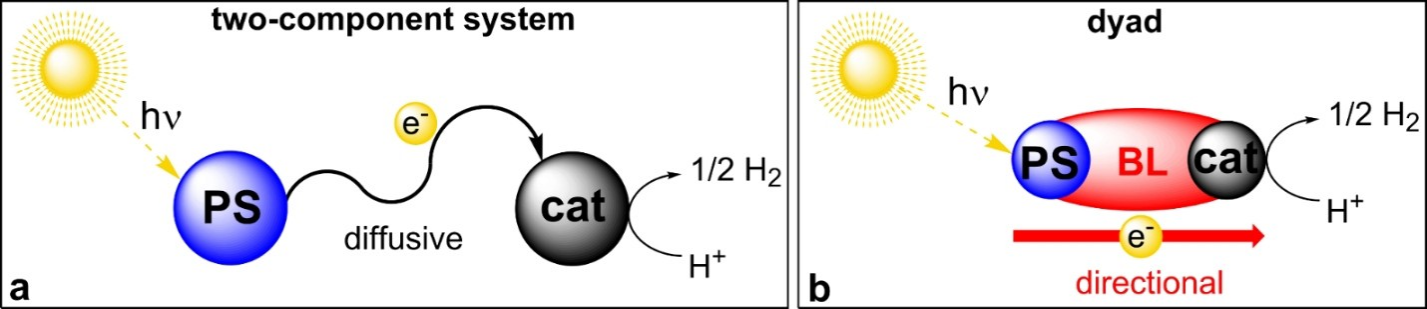
Schematic examples of light‐driven hydrogen evolution systems. a) A two‐component system with diffusive electron transfer and b) a dyad based on covalently linked photosensitizer and catalyst with directional electron transfer. PS: photosensitizer, cat: catalyst, BL: bridging ligand.

The global demand for sustainable hydrogen supply is constantly increasing. Hydrogen production by electrolysis uses electric energy derived from fossil fuels, wind, or solar energy sources. Photocatalysis instead uses sunlight directly and has a large potential to increase the provision of green energy. In homogenous photocatalytic hydrogen evolution reactions, a molecular photosensitizer (PS) is excited by the incident light and excited electrons are transferred to the catalyst (cat), which reduces protons to hydrogen (Figure 1a). Noble metal photosensitizers based on ruthenium^[1]^ and iridium^[2]^ have been widely studied together with noble metal catalysts like platinum.^[3]^ For common applications in the future, abundant and inexpensive metals such as iron or cobalt are required to ensure large area applications. In the past, cobalt was successfully applied not only in photocatalytic but also in electrocatalytic hydrogen evolution reactions.^[4]^ Interest in cobaloximes as catalysts in photochemical reactions evolved when Oishi et al. and later Hawecker et al. applied this catalyst class to light‐driven hydrogen production.^[5]^ In particular, chloro‐(pyridine)cobaloxime(III) (termed hereafter [Co]), which was first synthesised in 1907,^[6]^ has been studied for over a century and is widely applied as a catalyst.[^1b^, ^7^]

For these reasons, it proved to be a suitable candidate to synthesise molecular assemblies, also called dyads, and to study the interaction between a photosensitizer and a catalyst. In contrast to the two‐component system where the electron transfer is diffusion‐limited, in such dyads, photosensitizer and catalyst are covalently linked via a bridging ligand (BL). Here, the electrons can be transferred directionally as shown in Figure 1b. Heterometallic noble metal dyads have been widely studied in photocatalytic hydrogen evolution reactions.^[8]^ Mixed dyads composed of a noble metal photosensitizer and a cobalt catalyst are frequent subject of investigations.^[9]^ On the other hand, base metal photosensitizers are rare in photocatalytic proton reduction,^[10]^ and in particular in dyads.^[11]^ In iron photosensitizers, the small ligand field splitting causes a fast nonradiative deactivation of catalytically active metal‐to‐ligand charge‐transfer (MLCT) states via a lower‐lying metal‐centred (MC) state. Although reports exist that make use of such MC states in photochemical reactions,^[12]^ so far, they have been described as a sink for photoexcited electrons. Efforts try to increase the MLCT lifetimes of iron(II) photosensitizers from femtosecond timescales to the picosecond regime. Tuning of π‐acceptor properties to lower the energy of MLCT states and application of strong σ‐donors such as N‐heterocyclic carbenes (NHC) to destabilize MC states are two major strategies.^[13]^ Attempts to use these comparatively short lifetimes are, for example, provided by Gros et al., who have shown a successful electron injection into TiO_2_ from carboxylate‐functionalized tetra‐NHC iron(II) photosensitizers in dye‐sensitized solar cells.^[13]^ Moreover, we could provide a first indication for the future potential of iron photosensitizers in light‐driven proton reduction in combination with platinum as a proton reduction catalyst.^[10c]^ Thus, the directional electron transfer in a dyad is another promising attempt to reduce the disadvantage of short excited state lifetimes with respect to photochemical applications (Figure 1b). Here, a novel dyad employing a heteroleptic tetra‐NHC‐iron(II) photosensitizer and a cobaloxime catalyst is presented which shows photocatalytic proton reduction activity to explore further the potential of such assemblies for sustainable hydrogen production.

## Synthesis

A previous published iron‐cobalt dyad consists of iron(II) coordinated by bulky C^N^C ligand L1 (L1=2,6‐bis[3‐(2,6‐diisopropylphenyl)imidazol‐2‐ylidene]pyridine) with spatial demanding DIPP‐substitutes (DIPP=2,6‐diisopropylphenyl), N^N^N bridging ligand pyterpy (pyterpy=4’‐(4’’’‐pyridyl)‐2,2’:6’,2’’‐terpyridine), and Co^III^ dimethylglyoxime.^[11a]^ As described before, the application of carbene ligands increases the MLCT lifetime significantly in contrast to polypyridyl iron complexes. Therefore, the C^N^C bridging ligand BL (BL=2,6‐bis(3‐methyl‐imidazol‐2‐ylidene)‐4,4′‐bipyridine) is used instead of pyterpy. In addition to a C^N^C iron coordination environment, the central pyridine is functionalized by a second pyridine ring resulting in a 4,4’‐bipyridine motif that allows monodentate coordination to cobalt(III).

**Scheme 1:**
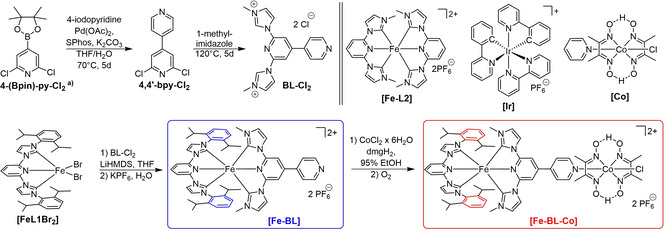
Synthesis of BL‐Cl_2_, [Fe−BL] and [Fe−BL−Co], structures of reference compounds [Fe−L2], [Ir]=[Ir(ppy)_2_(bpy)]PF_6_ and [Co]=Co(dmgH)_2_(py)Cl. Protons at the *meta*‐positions in 2,6‐diisopropylphenyl rings (coloured in blue and red) are used for calculating the dyad fraction in the ^1^H NMR study. 4‐(Bpin)‐py‐Cl_2_ was synthesised from 2,6‐dichloropyridine in an iridium‐catalysed C−H borylation.^[14]^

The three‐step synthesis route to a new carbene precursor ligand BL‐Cl_2_ is shown in the top part of Scheme 1. It starts with an iridium‐catalysed C−H borylation of 2,6‐dichloro‐4,4’‐pyridine.^[14]^ 4‐Borylated 2,6‐dichloropyridine (4‐(Bpin)‐py‐Cl_2_) is coupled to 4‐iodopyridine employing Suzuki coupling to give 2,6‐dichloro‐4,4’‐bipyridine (4,4’‐bpy‐Cl_2_) in 58 % yield. BL‐Cl_2_ is generated by nucleophilic aromatic substitution with 1‐methylimidazole. Vapor diffusion crystallization with MeOH‐Et_2_O gives blue needles suitable for X‐ray diffraction. The new heteroleptic bis‐C^N^C Fe^II^ complex [Fe−BL] is synthesised according to previous work.^[10c]^ Deprotonation of precursor salt BL‐Cl_2_ results in the free carbene ligand BL. Mixing of iron precursor [FeL1Br_2_]^[15]^ and carbene solution overnight, removal of solvent, and anion exchange with KPF_6_ in water gives the heteroleptic dyad precursor [Fe−BL] in 50 % yield after purification. This heteroleptic complex protocol is superior to stochastic approaches. Bimetallic dyad [Fe−BL−Co] is synthesised following the protocol published by Schrauzer et al.^[16]^ by addition of [Fe−BL] to a solution of cobalt(II)chloride hexahydrate and dimethylglyoxime in ethanol at 70 °C. Oxidation of Co^II^ to Co^III^ by air, filtration, and washing give [Fe−BL−Co] as a red powder in 86 % yield. Iron photosensitizer [Fe−L2], iridium photosensitizer [Ir(ppy)_2_(bpy)]PF_6_, and [Co], which are used in the following study as references are depicted in Scheme 1 as well.[^9e^, ^16^, ^17^]

## Results

### X‐ray diffraction

**Figure 2:**
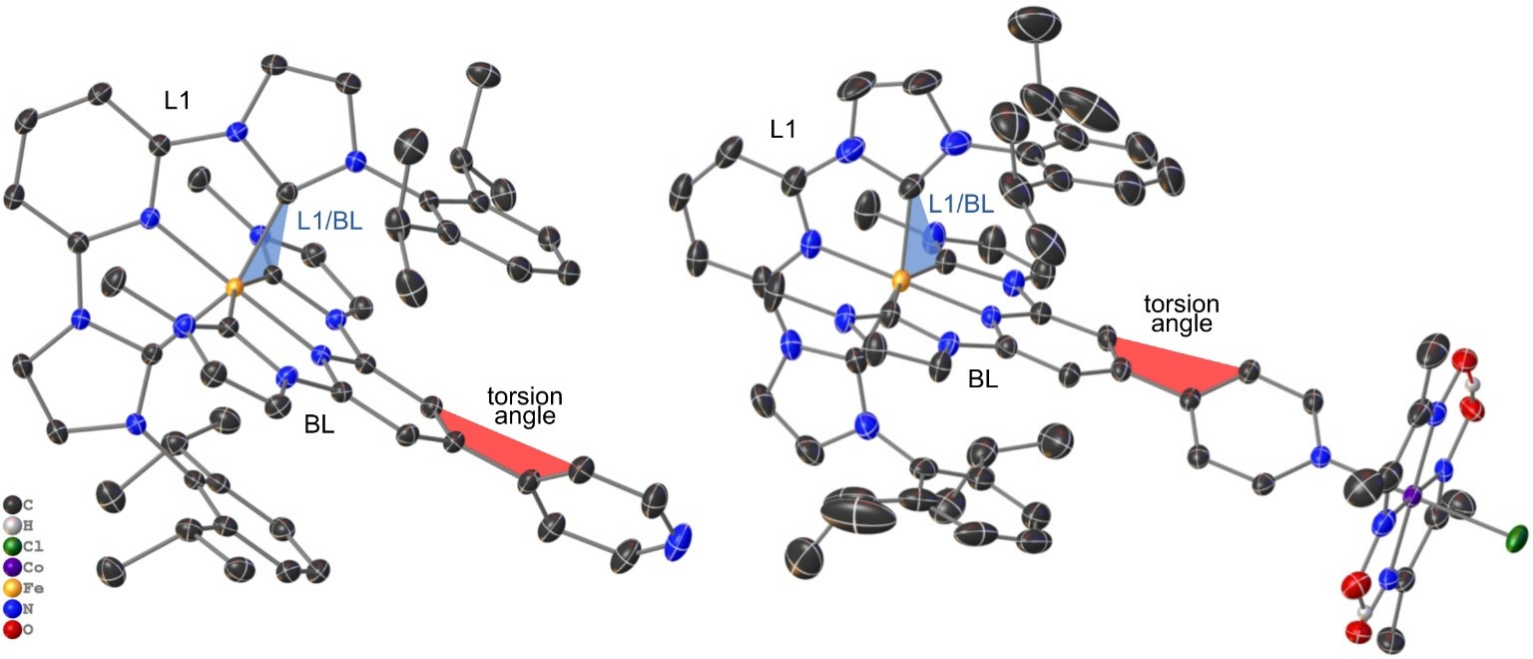
Molecular structures of [Fe−BL] (left) and [Fe−BL−Co] (right). Hydrogen atoms, except in the glyoxime moiety, anions and co‐crystalized solvent molecules are omitted for clarity. Anisotropic displacement ellipsoids are drawn at the 50 % probability level. The dihedral angle L1/BL and torsion angle in the 4,4’‐bipyridine motif are highlighted.

X‐ray crystal structures of [Fe−BL] and [Fe−BL−Co] are shown in Figure 2, the key crystallographic parameters are listed in Table 1. [Fe−BL] is coordinated by L1 and BL in a distorted octahedral geometry with a dihedral angle L1/BL of 92.37(4)°. Fe−N bond lengths for L1 and BL are similar with 1.9150(14) and 1.9152(14) Å, respectively, whereas Fe−C bond lengths in L1 and BL, Fe−C_L1_=1.9675(17) Å and Fe−C_BL_=1.9490(17) Å, differ slightly, due to the steric demand of DIPP substitutes in L1. Additionally, in the 4,4’‐bipyridine motif of BL, the two pyridine rings are twisted by 45.45(7)° against each other, which is well known for similar ligands with 4,4’‐bipyridine motifs.^[18]^ In contrast, both pyridine rings in BL‐Cl_2_ are only twisted by 12.77(9)° (cf. Figure S1 in the Supporting Information).

**Table 1:** Crystallographic data, DFT parameters, and EXAFS fitting results.

	Bond lengths [Å] and angles [°]						
	[Fe−BL]_XRD_	[Fe−BL]_calc_^[a]^	[Fe−BL]_EXAFS_^[b]^	[Fe−BL−Co]_XRD_	[Fe−BL−Co]_calc_^[a]^	[Fe−BL−Co]_EXAFS_^[b]^	[Co]_XRD_^[c]^
Fe−BLN_L1_	1.9150(14)	1.9305	2.112(24)	1.919(2)	1.9304	2.099(51)	−
Fe−BLN_BL_	1.9152(14)	1.9282		1.907(2)	1.9271		−
Fe−BLC_L1(av)_	1.9675(17)	1.9878	1.945(8)	1.970(3)	1.9878	1.942(11)	
Fe−BLC_BL(av)_	1.9490(17)	1.9686		1.953(3)	1.9681		−
Co−N_py_	−			1.968(2)	2.0086		1.959(2)
Co−N_dmgH(av)_	−			1.894(3)	1.9057		1.905
Co−Cl	−			2.2351(7)	2.1896		2.229(1)
Torsion_BL_	45.45(7)	43.3		30.61(5)	44.76		−

[a] DFT, def2‐TZVPP, PBEh‐3c, gas phase. [b] EXAFS, averaged over 4 C and 2 N, [c] Adapted from ref. [19].

In the case of [Fe−BL−Co], the bond lengths and coordination geometry of the iron centre are comparable to [Fe−BL]. Dihedral angle L1/BL is slightly increased to 94.06(7)°, probably due to packing effects. The pyridine‐pyridine twist angle in BL is reduced to 30.61(5)° when Co dimethylglyoxime is attached to [Fe−BL], which could be an indicator of a second‐order interaction between the two constituting metal fragments. The oxidation state of Fe^II^ in both compounds was confirmed by XANES measurements, and structural parameters extracted from EXAFS measurements are included in Table 1 for comparison (see the Supporting Information for details). Cobalt is equatorially coordinated by two dimethylglyoxime ligands which create a planar pseudo‐macrocycle with two hydrogen bonds as in reference compound [Co]. In the dyad, all Co−N bond lengths are comparable to Co−N lengths in [Co] as well as Co‐Cl bond length.

### Dissociation, association and photostability

The pyridine‐cobaloxime coordinative bond between photosensitizer and catalyst is the weakest link in the dyad. For an appropriate analysis of the spectroscopic and catalytic data, dyad dissociation in solution must be understood. Especially in acetonitrile, which is often used as a solvent in hydrogen evolution reactions, dissociation of the dyad into photosensitizer and acetonitrile‐coordinated cobalt dimethylglyoxime fragment is likely to occur. Mulfort et al. showed the high potential of small‐angle X‐ray scattering (SAXS) to investigate the dissociation of Ru^II^‐Co^II^ dyads in acetonitrile quantitatively.^[20]^ NMR experiments are established in the following to understand the stability of diamagnetic axially‐coordinated cobaloxime dyad [Fe−BL−Co] over a large concentration range. Upon coordination of cobalt dimethylglyoxime, changes in the chemical shift of the [Fe−BL] proton signals are observed, resulting in characteristic dyad proton signals, which are clearly distinguishable from photosensitizer proton signals.

**Figure 3:**
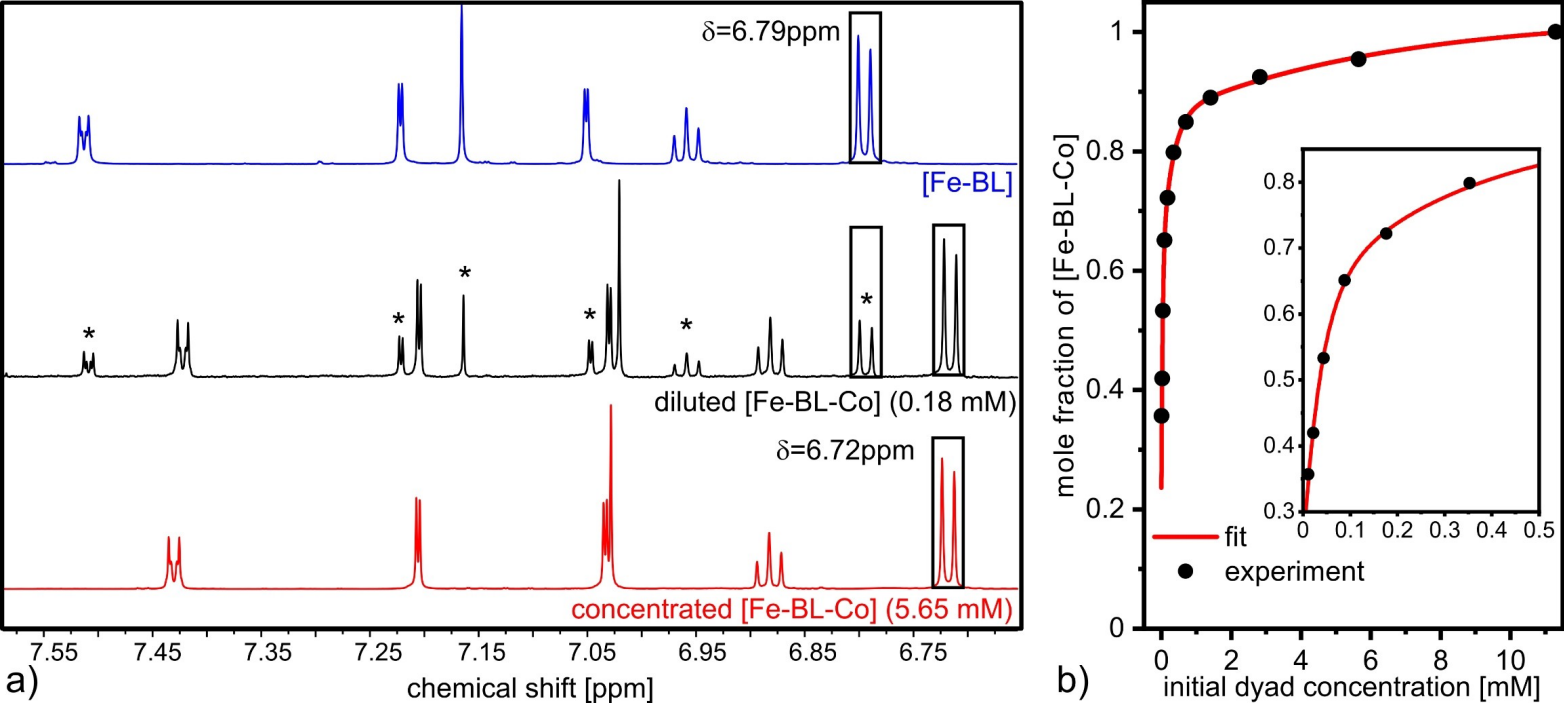
a) Insight into the aromatic region of the ^1^H NMR spectrum of dyad [Fe−BL−Co] (concentrated=red, diluted=black) and [Fe−BL] (blue) in CD_3_CN. Proton signals in diluted [Fe−BL−Co] solution originating from [Fe−BL] are marked with asterisks. b) Mole fraction of [Fe−BL−Co] calculated from the proton integral ratio (marked with squares in (a)) according to the equation: *f*_dyad_=*I*_dyad_/(*I*_dyad_+*I*_PS_). The total amount of species in solution is proportional to *I*_dyad_+*I*_PS_.

Figure 3a shows the relevant ^1^H aromatic region of 7.55‐6.65 ppm chemical shift for the dyad [Fe−BL−Co] in high (5.65 mM) and low concentration (0.18 mM) in comparison to a solution of [Fe−BL]. Complete ^1^H NMR spectra over the applied concentration range are shown in Figure S7. Integrals of the most intense signals of the protons in meta‐position of the DIPP‐phenyl ring (cf. Scheme 1) at *δ*(Fe−BL−Co)=6.72 ppm and *δ*(Fe−BL)=6.79 ppm are used as probes to investigate the dissociation process. In Figure 3b, the resulting dyad mole fraction as a function of the initial concentration of the dyad is shown. In concentrated solutions higher than 5 mM, the dyad [Fe−BL−Co] is intact to a degree of more than >95 %. Dilution of the solution leads to the appearance of proton signals corresponding to [Fe−BL], proving dissociation of the dyad [Fe−BL−Co] into [Fe−BL] and solvent‐coordinated cobalt dimethylglyoxime. In a solution with a concentration of 0.18 mM, 72 % of the dyad is still intact. [Fe−BL] is the dominant species in highly diluted solutions with concentrations lower than c=0.04 mM. In comparison to that, SAXS investigations on Ru^II^‐Co^II^ dyads by Mulfort and co‐workers yielded dyad stability ranges of 66 % to 90 % at comparable concentrations (∼2.5–5 mM) in acetonitrile.^[9g]^ The rather surprising observation of increased [Fe−BL−Co] dyad dissociation at lower concentrations observed here can currently only speculatively be assigned to increased competition of BL‐pyridine and acetonitrile for coordination at the Co center.

Equally, in‐situ dyad formation from a mixture of [Fe−BL] and [Co] in acetonitrile can also be studied in detail by ^1^H NMR. For this purpose, initially equimolar mixtures of [Fe−BL] and [Co] in [D_3_]acetonitrile were investigated in a concentration range from 0.02 to 5.6 mM. The corresponding ^1^H NMR spectra are shown in Figure S8, as well as the resulting correlation between the in‐situ formed dyad and the initial [Fe−BL] concentration (Figure S9). In fact, [Fe−BL−Co] is formed: 33 % of the dyad is present in highly concentrated solutions (5.6 mM), whereas only 17 % of the dyad is formed in the most diluted solution (0.02 mM). The mole fractions of the dyad formation from [Fe−BL] and [Co] and dyad dissociation of [Fe−BL−Co] do not correspond to each other. The apparent reason in the formation study is the pre‐coordination of Co^III^ by a pyridine ligand, which needs to be substituted by the pyridine function of [Fe−BL], for which the chemical driving force is rather low. However, the potential in‐situ formation of dyad has not yet been adressed for comparable systems containing pyridine‐functionalized photosensitizer and cobaloxime derivatives or has been prevented by methylation of terminal pyridine function in further experiments.^[21]^

With respect to the potential application of dyad assemblies in photocatalytic reactions, photostability is a crucial issue. To study it here, solutions of all relevant compounds in [D_3_]acetonitrile were irradiated with an AM 1.5 300 W xenon lamp^[10c]^ for 2.5 and 22 hours and analysed by ^1^H NMR spectroscopy. [Fe−BL] and [Co] are stable over the full irradiation time range (cf. Figures S12 and S13). In contrast, in the spectrum of [Fe−BL−Co], new signals appear after 2.5 hours, as shown in Figures S10 and S11, which are tentatively assigned to a destabilization of the pyridine‐cobalt bond or twist in the 4,4′‐bipyridine moiety caused by irradiation. Since all remaining signals are unchanged, a degradation of the dyad could be excluded. The proton NMR spectrum does not change with further irradiation for 22 hours, after which 79 % of [Fe−BL−Co] are still intact. Thus, photochemistry will be explained based on intact dyads below.

### Optical absorption spectroscopy

**Figure 4:**
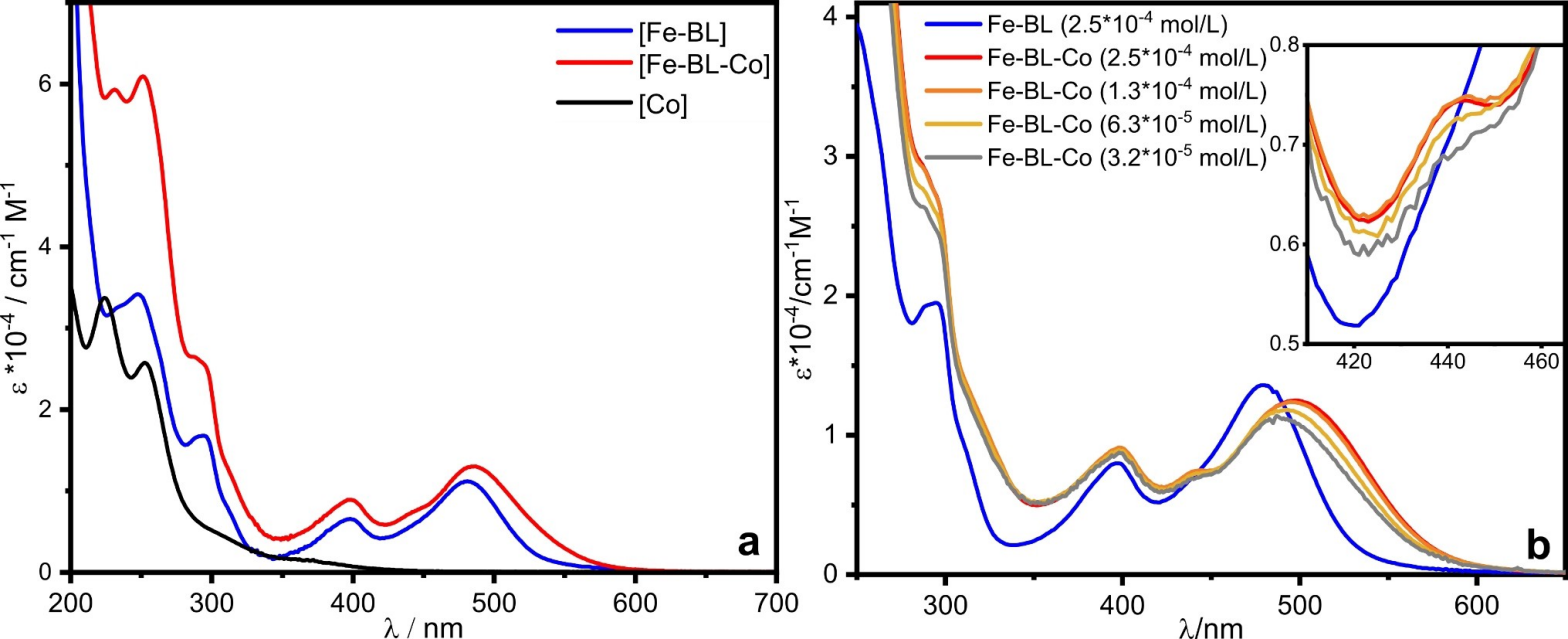
a) Steady‐state absorption spectra of [Fe−BL], [Fe−BL−Co] and [Co] in acetonitrile (1 ⋅ 10^−5^ mol/L). b) Steady‐state absorption spectra of [Fe−BL] (2.5 ⋅ 10^−4^ mol/L) and [Fe−BL−Co] (3.2 ⋅ 10^−5^–2.5 ⋅ 10^−4^ mol/L) in MeCN.

The optical absorption spectra of the investigated compounds in 1 ⋅ 10^−5^ mol/L acetonitrile solution are displayed in Figure 4a. The most important bands are listed in Table 2. In the UV region below 350 nm, typical π‐π*‐transitions dominate, and less intense bands between 350–600 nm can be assigned to MLCT bands.^[10c]^ In comparison to the homoleptic tetra‐NHC‐Fe^II^ reference complex [Fe−L2] (cf. Scheme 1),^[13a]^ the MLCT band of [Fe−BL] at 481 nm is bathochromic shifted by 21 nm (cf. Table 2), in agreement with the electron‐withdrawing character of the terminal pyridine ring.^[11a]^ In [Fe−BL−Co], the MLCT band is asymmetrically broadened to higher wavelengths compared to [Fe−BL] and a weak shoulder around 440 nm appears, which is absent in [Fe−BL] and [Co]. This shoulder is therefore assigned to an electronic interaction of iron and cobalt fragments in [Fe−BL−Co]. Concentration‐dependent UV/Vis measurements of the dyad were also conducted in the range from 3.2 ⋅ 10^−5^ to 2.5 ⋅ 10^−4^ mol/L, and the data is presented in Figure 4b in comparison to [Fe−BL] (2.5 ⋅ 10^‐4^ mol/L). The shoulder around 440 nm is more pronounced at higher concentrations, which is confirmed as the spectral signature of electronic structure alterations in the dyad compared to the photosensitizer. Additionally, the low‐energy feature at 486 nm in diluted solution is bathochromic shifted by 13 nm when the concentration is increased. This is in accordance with a larger mole fraction of the dyad at higher concentrations and concluded from the NMR results. When the respective photosensitizer fractions are subtracted from the spectra, the maxima of the lowest‐energy band shift around 510 nm for all concentrations (cf. Figure S15). The resulting difference spectra represent the UV/Vis properties of [Fe−BL−Co] without photosensitizer influence very well.

**Table 2:** Physicochemical properties and computed electrochemical data.

Compound	*λ*_abs_ [nm] (*ϵ* [10^3^ cm^−1^ ⋅ M^−1^])^[a]^	*E*_ox_^[b]^ [V] (computed Δ*E*_ox_ [eV])		*E*_red_^[b]^ [V]		Δ*E*p^[c]^ [V]
[Fe−BL]	294 (16.8), 398 (6.6), 481 (11.2)		0.47(r),(0.38)		−1.97(r)	2.45
[Fe−BL−Co]	292 (26.0), 398 (8.9), 486 (13.0)	0.77(r)	0.50(r),(0.47)	−0.91(ir), −1.46(qr)	−1.94(r)	2.44
[Fe−L2]^[d]^	287 (31.4), 393 (9.0), 460(15.9)		0.41(r)		−2.34 (ir)	2.75
[Co]^[e]^	224 (3.4), 253 (2.6)	0.73(r)		−1.04 (ir), −1.50 (qr)

[a] Measured at 25 °C and 1 ⋅ 10^−5^ mol/L in MeCN. [b] Measured at 25 °C in MeCN (1 ⋅ 10^−3^ mol/L) vs Fc/Fc^+^. [c] Δ*E*_p_=*E*_ox_(Fe)−*E*_red_(Fe_ligand_), band gap determined by cyclic voltammetry. [d] Taken from ref. [13a]. [e] Electrochemical data taken from ref. [11a].

### Cyclic voltammetry and spectro‐electrochemistry

**Figure 5:**
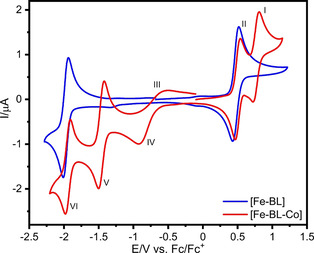
Cyclic voltammograms of [Fe−BL] and [Fe−BL−Co] in MeCN/[*n*Bu_4_N][PF_6_] (100 mV/s, 1 ⋅ 10^−3^ mol/L).

The cyclic voltammograms of [Fe−BL] and [Fe−BL−Co] in MeCN are shown in Figure 5. All potentials are referenced relative to the Fc/Fc^+^ couple. For [Fe−BL], the reversible oxidation at +0.47 V is assigned to the Fe^II^/Fe^III^ couple. A reversible reduction at −1.97 V is also detected. Although NHC‐ligand‐based reductions are usually irreversible, like in [Fe−L2] (−2.34 V, Table 2), we attribute this reduction to the presence of the bipyridine moiety in the BL ligand.^[22]^

The cyclic voltammogram of [Fe−BL−Co] reveals additional redox events that originate from the coordination of cobalt dimethylglyoxime. On the anodic site, there are two redox processes. At +0.77 V (I, Figure 5), dimethylglyoxime ligand oxidation occurs, which is more anodically shifted than in the reference complex [Co] (+0.73 V, cf. Table 2).^[11a]^ Thus, a lower electron density at the glyoxime moiety in [Fe−BL−Co] is found due to a lower Lewis basicity of [Fe−BL] in comparison to the pyridine ligand in [Co]. The Fe^II^/Fe^III^ transition (II, Figure 5) in the dyad occurs at +0.50 V, that is, a slightly higher value than in [Fe−BL]. It is attributed to the lowering of the Fe 3d localized dπ orbital energies due to cobalt coordination. On the cathodic site, upon reduction of the cobalt centre at −0.91 V, the chloride ligand is abstracted, which results in an irreversible Co^III^/Co^II^ wave (IV, Figure 5) with the corresponding anodic transition at −0.50 V (III, Figure 5).^[23]^ A quasi‐reversible Co^II^/Co^I^ reduction takes place at −1.46 V (V, Figure 5).^[7a]^ Additionally, the reversible BL‐based reduction is anodically shifted from −1.97 V in [Fe−BL] to −1.94 V (VI, Figure 5) in the dyad which confirms the improved π‐accepting capability of the ligand, induced by the cobalt moiety. In comparison to [Co] (−1.04 V and −1.50 V) both Co‐based reductions are anodically shifted. This indicates a stabilization of the Co 3d dπ* orbital energies of the cobalt centre in [Fe−BL−Co] with respect to cobaloxime. These CV results are summarized by a shift of electron density from the dimethylglyoxime fragment and iron centre to the cobalt centre and bridging ligand upon dyad formation.

**Figure 6:**
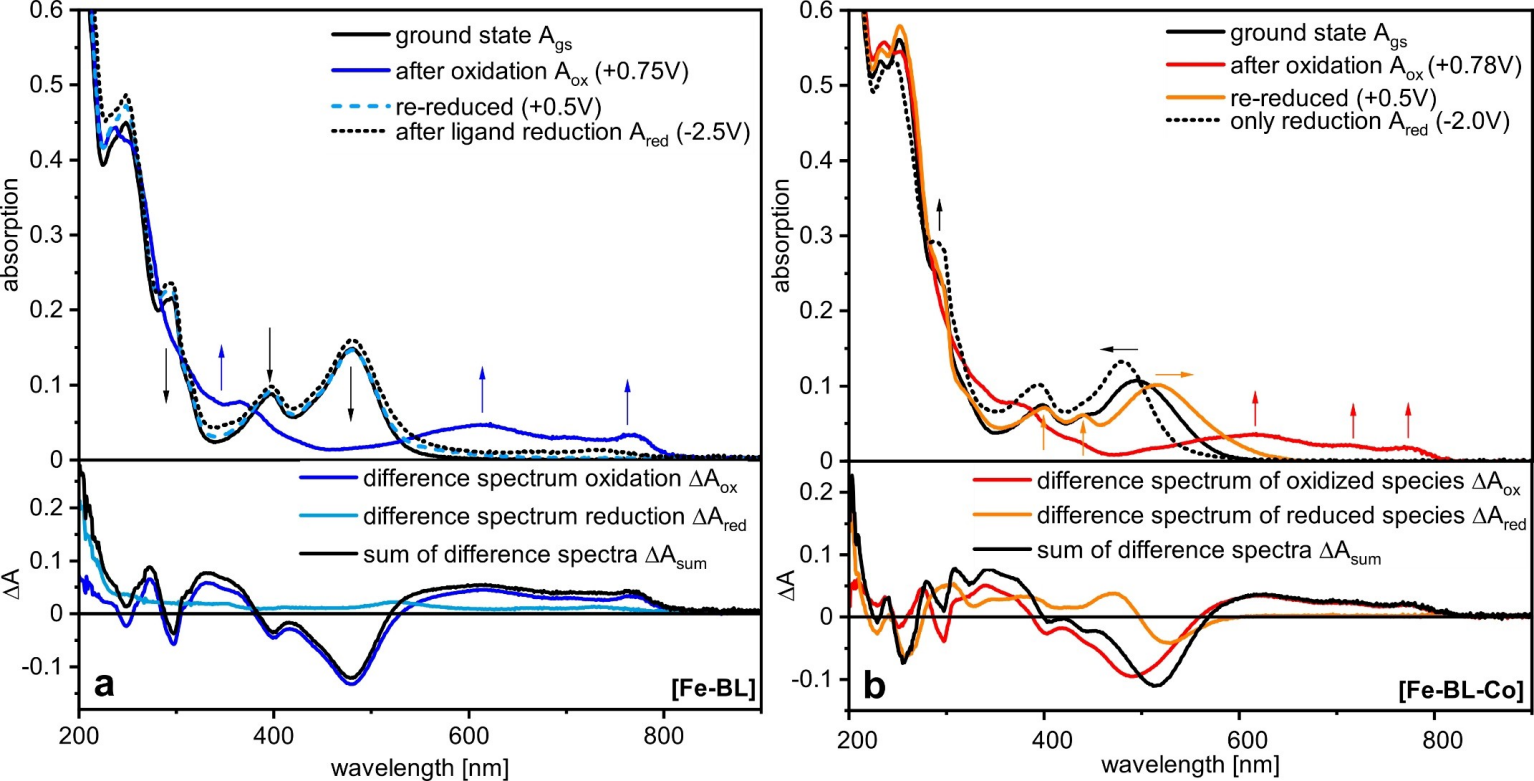
a) Top: UV/Vis spectra of [Fe−BL] (1 ⋅ 10^−4^ mol/L), after oxidation at +0.75 V, re‐reduction at +0.50 V, and ligand‐based reduction at −2.5 V. Bottom: Difference spectra of oxidized/reduced [Fe−BL] and linear combination. b) Top: UV/Vis spectra of [Fe−BL−Co] (1 ⋅ 10^−4^ mol/L), after oxidation at +0.78 V, re‐reduction at +0.50 V and ligand‐based reduction at −2.0 V. Bottom: Difference spectra of oxidized/reduced [Fe−BL−Co] and linear combination.

Spectro‐electrochemical experiments give further insights into the redox processes and additionally provide an important tool for the analysis of transient optical absorption data. The optical absorption behaviour of [Fe−BL] in the ground state *A*_gs_, after bulk oxidation *A*_ox_ and reduction *A*_red_ is shown in Figure 6a. Oxidation at +0.75 V reduces the intensity of the bands at 398 and 481 nm, and two broad bands appear in the range of 515–800 nm (cf. Figure 6a, *A*_ox_), which is in agreement with the values of LMCT bands in Fe^III^ complexes.^[24]^ Application of −2.5 V cathodic potential does not cause any considerable change to the absorption spectrum of [Fe−BL] (cf. Figure 6a, *A*_red_). The difference spectrum of oxidized species Δ*A*_ox_, resulting from *A*_ox_−*A*_gs_, emphasises the spectral changes upon metal oxidation and is used for spectral comparison with transient optical absorption spectroscopy below. The difference spectrum of reduced species Δ*A*_red_, resulting from *A*_red_−*A*_gs_, emphasises the spectral changes upon ligand reduction. The sum Δ*A*_sum_ of difference spectra of oxidized Δ*A*_ox_ and reduced species Δ*A*_red_ (Δ*A*_sum_=Δ*A*_ox_+Δ*A*_red_) shows the domination of oxidized spectral features (cf. Figure 6a, bottom). Slightly different behaviour is found for the dyad. As for [Fe−BL], oxidation at +0.78 V causes the disappearance of the MLCT bands, and LMCT bands of Fe^III^ can be observed (Figure 6b, *A*_ox_). Since the potential of +0.78 V is higher than both redox waves assigned to oxidation processes in cyclic voltammetry (0.50 V, 0.77 V), equivalents of more than one electron are transferred.

Accordingly, upon re‐reduction at +0.50 V, a new species is formed, which also has three bands between 350–600 nm as in the original ground state spectrum *A*_gs_ of [Fe−BL−Co]. Two bands have similar peak positions, 401 and 439 nm, whereas the band lowest in energy is bathochromic shifted to 515 nm compared to 495 nm in *A*_gs_ of [Fe−BL−Co]. At the concentration of 1 ⋅ 10^−4^ mol/L, we expect about 66 % of the dyad in solution deduced from the NMR study. The strong bathochromic shift of 20 nm indicates a more intact dyad than at the beginning of the experiment. This is also in agreement with the obtained [Fe−BL−Co] UV/Vis spectra after photosensitizer subtraction with a band maximum at 510 nm (cf. Figure S15). Therefore, we assign this species after one oxidation‐reduction cycle to a re‐assembled dyad that exceeds 66 % in solution. Ligand reduction is investigated with a fresh dyad solution applying a potential of ‐2 V. The resulting absorption spectrum *A*_red_ matches the spectrum of [Fe−BL] well, therefore this observation is assigned to dissociation of [Fe−BL−Co]. Both [Fe−BL] and [Fe−BL−Co] show thus the same behaviour when applying a cathodic potential. The resulting difference spectra Δ*A*_ox_ and Δ*A*_red_ of oxidized and reduced species highlight the spectral changes upon oxidation and reduction (cf. Figure 6b, bottom). The sum of them, Δ*A*_sum_, reveals the growth of positive absorption bands before 400 nm and after 580 nm due to the presence of LMCT bands. Negative bands from 400–580 nm clearly originate from the vanishing of MLTC bands.

### Computational analysis

**Figure 7:**
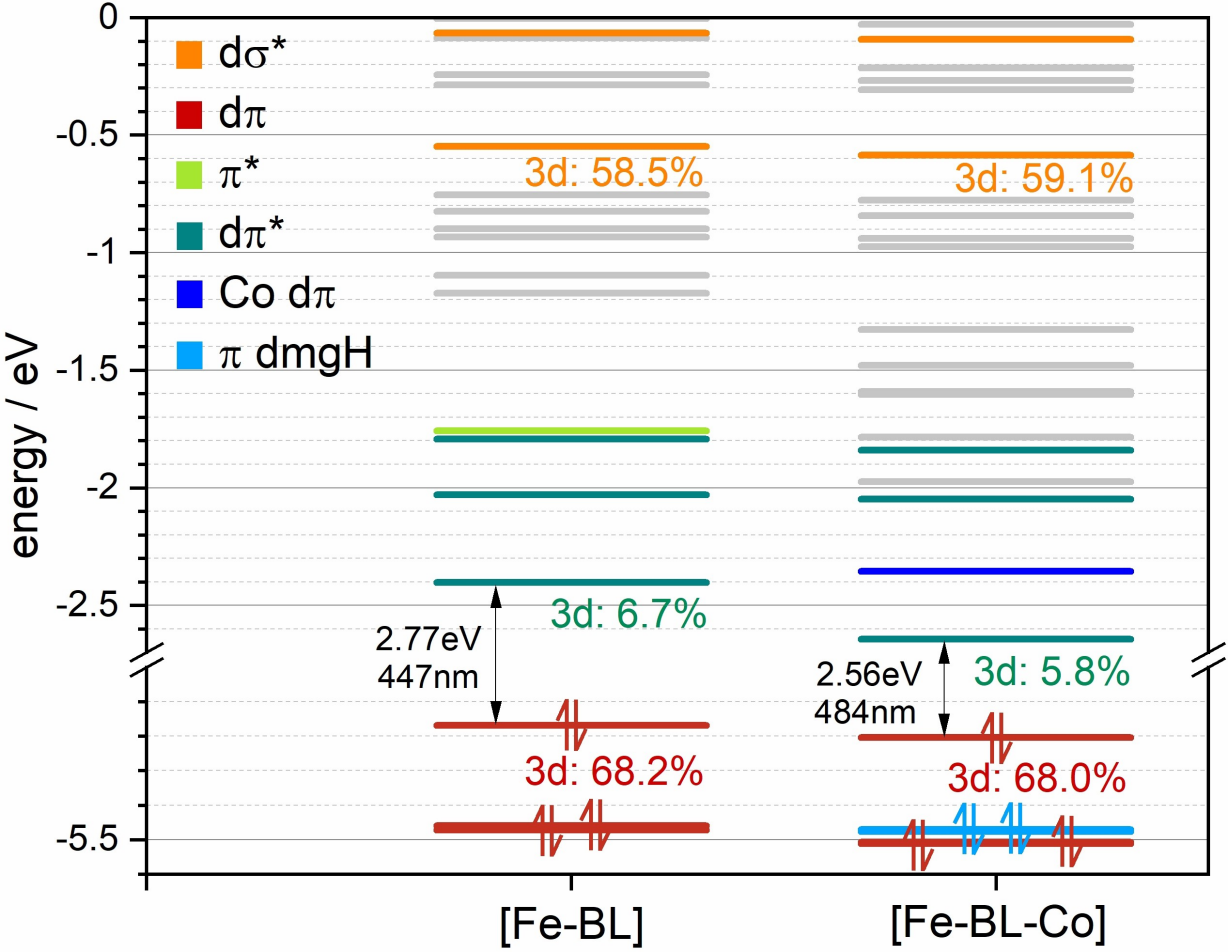
Molecular orbitals of [Fe−BL] and [Fe−BL−Co]; TPSSh‐D3, def2‐TZVPP, SMD(MeCN). Computed HOMO‐LUMO gaps are indicated. The average %Fe character is given for dπ, dπ*, and dσ* orbitals.

Electronic structure properties of [Fe−BL] and [Fe−BL−Co] are accessed by DFT calculations (see the Experimental Section for details). A good agreement between experimental and computational bond lengths and angles is achieved, as given in Table 1. Molecular orbital (MO) eigenvalues of [Fe−BL−Co] and [Fe−BL] are compared in Figure 7. Average iron‐3d contributions are given for dπ, dπ*, and dσ* orbitals. HOMO to HOMO‐2 reflect π‐bonding interactions (dπ) between iron t_2g_‐like and ligand π* orbitals in [Fe−BL], whereas in [Fe−BL−Co] ligand π‐type orbitals (HOMO‐1 and HOMO‐2) of the dmgH ligand are located in between the dπ levels (HOMO, HOMO‐3 and HOMO‐4), which is known for similar Fe−Co dyads with a Co(dmgH)_2_ motif.^[11a]^ LUMO to LUMO+2 of [Fe−BL] reflect the analogue π‐antibonding interactions (dπ*) between iron t_2g_‐like and ligand π* orbitals, in agreement with the typical description of a π‐acceptor ligand interacting with a pseudo‐octahedral Fe^II^ centre. In the case of [Fe−BL−Co], LUMO, LUMO+2, and LUMO+4 reflect dπ* orbitals instead, due to Co dπ and ligand localized levels in between. Since both complexes are coordinated in a distorted octahedron, dπ, dπ*, and dσ* orbitals are not degenerated. To be able to compare MO energies of both complexes, average MO eigenvalues and average %Fe character are therefore used in the following. The average dπ energy of −5.37 eV in [Fe−BL] is slightly destabilized compared to [Fe−BL−Co] with ‐5.41 eV, illustrating an increase of the ligands electron affinity or acceptor capability through linkage to a Co(dmgH)_2_ motif. The average %Fe character of the dπ orbitals is basically identical, with only a slight decrease in the dyad by 0.2 %. Based on this number, a slight increase in the Fe−BL ligand π‐acceptor interaction strength can be deduced, in agreement with a previous study.^[11a]^ Both interpretations are further substantiated by a decreasing HOMO‐LUMO gap from 2.77 eV in [Fe−BL] to 2.56 eV in [Fe−BL−Co], reflecting a MLCT stabilization (−0.21 eV, 37 nm) in the Franck‐Condon region of [Fe−BL−Co]. This agrees with the observation made in the concentrated UV/Vis spectra, where the MLCT feature in [Fe−BL−Co] is redshifted compared to [Fe−BL] by up to 29 nm (cf. Figure S15). The σ‐donor interaction strength in [Fe−BL−Co] is slightly increased since the average %Fe character of the dσ* is increased by 0.6 % compared to [Fe−BL]. Unfortunately, the average dσ* energy is decreasing in [Fe−BL−Co] compared to [Fe−BL], reflecting a weaker donor‐capability of the free ligand, which is compensating the increasing interaction strength in the dyad, leading to equal metal‐centred dπ‐dσ* gaps in [Fe−BL] and [Fe−BL−Co] of 4.62 eV and therefore comparable MC energies in the Franck‐Condon region.

To allow a more holistic view on the electronic structure, oxidation potentials (Δ*E*_ox_) of the Fe^II^/Fe^III^ couple in the photosensitizer and the dyad are simulated with the same series of calculations shown above (cf. Table 2). Δ*E*_ox_ are estimated by the energy difference between the Fe^II^ and Fe^III^ states in the solvated Fe^II^ ground state structure of the given iron complexes. All computed potentials were corrected by the computed ferrocene potential of 4.50 eV.[^11a^, ^25^] An oxidation potential +0.38 eV is computed for [Fe−BL]. Compared to the experimental redox potential *E*_ox_(Fe−BL)=0.47 V, a deviation of 0.09 V is observed, which is in the range of previous studies on tetra‐NHC iron complexes applying this approximation.^[25b]^ A better agreement is found for the dyad with a computed oxidation potential of 0.47 eV which compares well with the experimental value of 0.50 V. The increased oxidation potential of the Fe^II^/Fe^III^ couple (0.47 vs. 0.50 V) from [Fe−BL] to [Fe−BL−Co] is nevertheless in accordance with a stabilization of the Fe 3d dπ orbitals obtained by molecular orbital energy calculations (cf. Figure 7). Additionally, it indicates that the iron centre is more difficult to oxidize which is also reflected by a reduced %Fe character. Computed reduction potentials show less agreement with the experiment and therefore neglected in the discussion. However, anodic shift of BL‐based reduction from −1.97 V in [Fe−BL] to −1.94 V in [Fe−BL−Co] in CV measurements is supported by the stabilization of lowest ligand localized dπ* orbital (LUMO) in the dyad by −0.24 eV (cf. Figure 7).

### Excited‐state characterization

**Figure 8:**
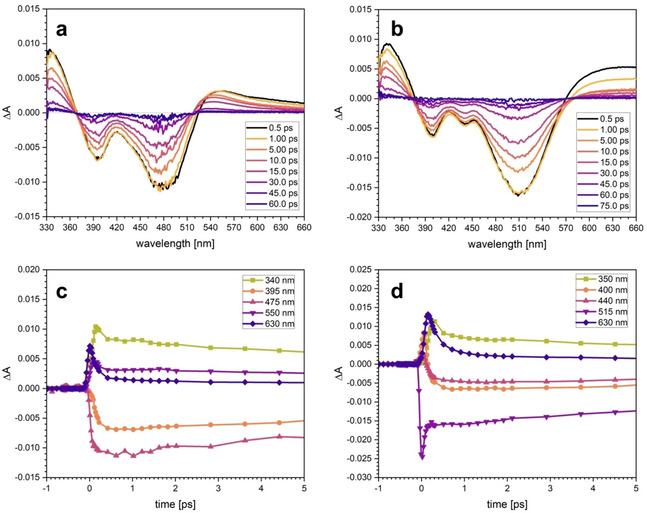
Optical TA spectra of a) [Fe−BL] and b) [Fe−BL−Co] excited at 515 nm. First 5 ps of the evolution of kinetics at selected wavelengths for c) [Fe−BL] and d) [Fe−BL−Co] ].

Excited state dynamics upon light irradiation was studied using ultrafast femtosecond optical transient absorption (TA) spectroscopy.^[26]^ To exclude dyad dissociation, measurements were conducted with 10 mM solutions in MeCN. For the following discussion, it is important to note that the steady‐state spectrum of [Co] (cf. Figure 4a) and TA spectra of [Co] (Figure S27) reveal that [Co] has no absorption at the excitation wavelength of 515 nm used here. Accordingly, [Co] has no spectral contribution to the excited band of [Fe−BL−Co]. The excited state behaviour of [Fe−BL] and [Fe−BL−Co] at representative time delays and the relevant kinetics at selected wavelengths after excitation at 515 nm are shown in Figure 8.

While both TA spectra of [Fe−BL] and [Fe−BL−Co] exhibit clear excited state absorption (ESA) signals in the blue spectral region (<370 nm) with nearly identical absorbance levels, the ESA signals observed in the red spectral region (>520 and >580 nm, respectively) substantially differ from each other. In the case of [Fe−BL] (Figure 8a), the spectral region between 370–520 nm is dominated by the ground state bleach (GSB) bands, and the maximum of the ESA band is observed around 545 nm. The time evolution of this band shows a gradual blue shift and band narrowing on a timescale of roughly 5 ps. The TA spectra of [Fe−BL−Co] show a pronounced redshift by 30–40 nm of the GSB corresponding to a depopulation of the MLCT band and the ESA signal. This corresponds to a change by around 0.17 eV, which is in very good agreement with the DFT‐predicted change in the HOMO‐LUMO bandgap between [Fe−BL] and [Fe−BL−Co]. In the case of [Fe−BL−Co], the different spectral evolution of the red ESA region is also reflected by significant differences in their kinetics. The amplitude of the red ESA of [Fe−BL−Co] is more intense and significantly broader than in [Fe−BL]. Another important difference between [Fe−BL] and [Fe−BL−Co] are the kinetics observed around 400 nm. While both of the bands appear to be GSB bands, the kinetic data of [Fe−BL−Co] at these wavelengths exhibit mixed features by gaining amplitudes above zero and then by decaying below zero (cf. Figure 8d).

To identify the excited species of [Fe−BL] and [Fe−BL−Co], the time constants of the decay process were obtained by fitting of selected kinetic traces (cf. Figure 8c and d). To have a consistent picture of the excited species appearing in each spectral region, the kinetics at the highest absorption point of each ESA and GSB band are globally fitted (cf. Figures S30 and S31). The fits are obtained using an exponential function with three time constants, an offset and a simultaneous convolution of the IRF function (assuming Gaussian temporal envelope of IRF). The time constants obtained from individual kinetic patterns are used as guess parameters for global analysis (GA) in a wide spectral range. This consist of a standard single value decomposition (SVD) method in the selected TA spectral range of 330–660 nm (see the Supporting Information for further details) followed by the extraction of decay associated spectra (DAS) for each time constant retained in the kinetic model.

Both [Fe−BL] and [Fe−BL−Co] exhibit very fast decaying components (*τ*_1_) that are smaller than the instrument response function (IRF=0.12 ps) and include artefacts close to time‐zero.^[27]^ As *τ*_1_ does not influence the results obtained in the GA, it is neglected in the discussion. All the kinetics analysed in the GSB, and the blue ESA regions are dominated by the presence of a short decay constant of 140 fs (*τ*_2_) for both [Fe−BL] and [Fe−BL−Co]. The longest time constants (*τ*_4_), corresponding to the recovery of the ground state signal, are found to be 17.1 ps for [Fe−BL], which is prolonged to 19.8 ps for [Fe−BL−Co], as summarized in Table 3 (see also Figure S28 and Tables S14 and S15).

**Table 3:** Time constants of DAS of optical transient absorption spectroscopy measured at 10 mM in MeCN.

Compound	DAS1, *τ*_1_ [ps]	DAS2, *τ*_2_ [ps]	DAS3, *τ*_3_ [ps]^[a]^	DAS4, *τ*_4_ [ps]
[Fe−BL]	<IRF	0.14	1.7	17.1
[Fe−BL−Co]	<IRF	0.14	1.0	19.8

[a] Obtained from global fitting. The uncertainty of the *τ*_3_ for both complexes was ±0.1 ps.

The DAS of [Fe−BL] and [Fe−BL−Co] are qualitatively analysed in comparison to the steady‐state UV/Vis absorption and the difference spectra of electrochemically oxidised species (cf. Figures S28 and S29). In the case of [Fe−BL], the difference spectrum of oxidised species ΔA_ox_ shares very common features with the evolution of transient signal in the 100–200 fs range, where the red ESA is most prominently present. In the case of [Fe−BL−Co], this similarity appears at a larger time delay at around 500 fs.

While the qualitative analysis of the TA spectra of [Fe−BL] and [Fe−BL−Co] indicate substantial differences between the ESA signals, similar time constants obtained from DAS suggest the same nature of the excited states. To further resolve the spectral features of [Fe−BL] and [Fe−BL−Co] and more reliably identify the excited state species, global fitting of the data within the full spectral range was performed according to a previously proposed analysis method (see the Supporting Information for further details).^[28]^ The most important outcome is the existence of an additional contribution to the decay cascade with a time constant *τ*_3_, which is absent in DAS constructed by SVD.

**Figure 9:**
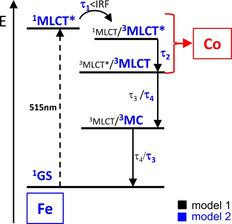
Proposed reaction scheme for the iron part of [Fe−BL] and [Fe−BL−Co]. Potential electron transfer from ^1/3^MLCT^(^*^)^ to the cobalt moiety is indicated with a red arrow. The relaxation pathway according to the more favourable model 2 is highlighted in blue; model 1 is in black.

It is a broadly recognised feature of TA kinetics that the decay components smaller than IRF, such as *τ*_1_, are an indication of a hot singlet state (^1^MLCT*).^[29]^ Starting from this point, there are two possible ways to explain the de‐excitation pattern observed in TA data (Figure 9):

(1) $${}^{1}\mathrm{MLCT}{}^{*}\;\overset{\tau_{1}}{\underset{}{\to }}\;{}^{1}\mathrm{MLCT}\;\overset{\tau_{2}}{\underset{}{\to }}\;{}^{3}\mathrm{MLCT}{}^{*}\;\overset{\tau_{3}}{\underset{}{\to }}\;{}^{3}\mathrm{MLCT}\;\overset{\tau_{4}}{\underset{}{\to }}\;\mathrm{GS}$$

(2) $${}^{1}\mathrm{MLCT}{}^{*}\;\overset{\tau_{1}}{\underset{}{\to }}\;{}^{3}\mathrm{MLCT}{}^{*}\;\overset{\tau_{2}}{\underset{}{\to }}\;{}^{3}\mathrm{MLCT}\;\overset{\tau_{4}}{\underset{}{\to }}\;{}^{3}\mathrm{MC}\;\overset{\tau_{3}}{\underset{}{\to }}\;\mathrm{GS}$$

According to the first option, *τ*_2_ with only positive and even higher amplitudes than *τ*_3_ in ESA regions is attributed to a relaxation process from the ^1^MLCT state to a hot ^3^MLCT*, which can further relax to the lowest ^3^MLCT state within 1–2 ps. Since intersystem crossing in vibrationally excited Franck‐Condon region can occur in tens of fs,^[30]^ more probable is that *τ*_2_, as indicated in option (2), represents a transition to and cooling within the triplet MLCT scaffold to the final ^3^MLCT on the bottom of the vibrational potential energy surface.[^29a^, ^29b^] This conclusion excludes also the possibility of a ^1^MLCT → ^3^MLCT transition. According to the reported transient absorption spectra of [Fe−L2] and other tetra‐NHC iron(II) complexes, in both models (1) and (2), we assign the longest time constants *τ*_4_ to the lifetime of the ^3^MLCT state.[^13a^, ^25^] By modifying the backbone of the bis‐NHC pyridine ligand with an additional pyridine ring, the ^3^MLCT lifetime is doubled from 8 ps in [Fe−L2] to 17.1 ps in [Fe−BL]. The increased aromatic system stabilizes the MLCT state, which agrees with the redshift of 21 nm in the UV/Vis spectra (cf. Table 2).^[31]^ Moreover, the ^3^MLCT lifetime is elongated by the attachment of a cobaloxime moiety, which is also supported by UV/Vis and DFT calculations. This effect has been proven by a similar iron(II) system bearing only two NHC functions.^[11a]^ There, the ^3^MLCT lifetime could be elongated by 0.3 ps. In this study, we could show that the stabilization of the ^3^MLCT state by a second metal is more pronounced with four NHC functions (2.7 ps). Thus, modification of the iron photosensitizer by four carbenes and cobalt coordination improves the excited state lifetime cooperatively. Model (1) assumes that the ^3^MLCT state is the final excited state before the transition to the ground state. This would exclude the presence of MC states and would be in contrast to several studies on NHC complexes of iron, where always MC states are involved in the excited state landscape.[^17^, ^32^] On the other hand, in the model (2), the long‐lived ^3^MLCT state decays further to a ^3^MC, characterized by the lifetime *τ*_3_. However, due to the apparent spectral overlapping of ^3^MC and ^3^MLCT signals and a lack of distinguishable characteristic features, it becomes challenging to disentangle the two deactivation pathways from TA data clearly. As the presence of excited ^3^MC states in NHC‐iron complexes has been discussed in many studies, model (2) is, however, more likely. Still, the final proof for the existence of such an MC state has to be proven by more sensitive methods like ultrafast X‐ray emission spectroscopy.^[33]^

Drawing a complete reaction scheme of [Fe−BL−Co] from the TA experiment is unfeasible since the [Co] moiety of [Fe−BL−Co] remains optically dark, although, from spectro‐electrochemical experiments, it is known that formed Co^II^ or Co^I^ species have different optical features.^[34]^ This means that an electron potentially transferred from iron moiety to cobalt moiety is not visible in the TA spectra of [Fe−BL−Co]. However, the increased lifetime of the ^3^MLCT state in [Fe−BL−Co] suggest a significant level of stabilization of internal charge‐transfer states in the dyad with simultaneous decrease of the bandgap size, which enhances the probability of the electron transfer to [Co] to occur. Further attempts by element‐specific X‐ray spectroscopy will shed light on the optically dark cobalt site in the future.^[35]^ Additionally, as it will be shown below, the photocatalytic proton reduction is increased in [Fe−BL−Co] compared to [Fe−BL]. Although this observation is surprising in spite of the observed lifetimes, the higher activity of the dyad is a non‐negligible indication of an intramolecular electron transfer.

### Photocatalytic proton reduction

**Figure 10:**
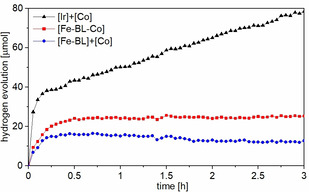
Photocatalytic proton reduction. Comparison of [Fe−BL−Co] (red) and the two‐component systems [Fe−BL]+[Co] (blue) and [Ir]+[Co] (black). Reaction conditions: 0.25 mM PS/cat/dyad in MeCN/water (1 : 1, 20 mL, 5 % triethanolamine) under argon. Irradiated by a Xe lamp (300 W) with an AM1.5 global filter. The negative slope of the blue curve originates from temperature correction in combination with the small volumes produced in this experiment.

The described systems were tested in a standardized proton reduction setup (see the Supporting Information for details).^[10c]^ The photocatalytic performance of the dyad [Fe−BL−Co] and the analogue two‐component systems [Fe−BL]+[Co] in comparison to [Ir(ppy)_2_(bpy)]PF_6_+[Co]^[9d]^ are shown in Figure 10, numeric values characterizing the activity are summarized in Table 4.

**Table 4:** Investigation of two‐component systems and dyad based on iron or iridium as photosensitizer and cobalt as the catalyst.

PS	cat	TON_PS_^[b]^	TON_cat_^[c]^	*V*(H_2_) [mL]	σ^[d]^ [mL]	*n*(H_2_)^[e]^ [μmol]
[Fe−BL−Co]		10	5	0.62	0.15	25
[Fe−BL]+[Co]		6	3	0.39	0.03	16 (0.75 h)
[Ir]+[Co]		31	16	1.92	−	78

[a] Reaction conditions: photosensitizer, catalyst or dyad (5 μmol) in MeCN/water (1 : 1, 20 mL) and 5 % TEOA, 25 °C, irradiation with Xe light (300 W) and AM1.5 global filter, 3 h. [Ir]=[Ir(ppy)_2_(bpy)]PF_6_. [b] TON_PS_=2*n*(H_2_)/*n*(PS). [c] TON_cat_=*n*(H_2_)/*n*(cat). [d] *σ*=standard deviation. [e] Moles of produced hydrogen *n*(H_2_) with *V*_m_=24 471 mL/mol.

[Fe−BL−Co] produces 25 μmol H_2_, which corresponds to a turnover number (TON) of 5 with respect to the catalyst, and 10 for the photosensitizer. The hydrogen evolution rate decreases significantly after 45 minutes. [Fe−BL]+[Co] produces 16 μmol H_2_ within 45 minutes, which corresponds to TONs of 3 and 6 for the catalyst and photosensitizer, respectively. The activity of the two‐component system is thus reduced by a factor of two compared to the dyad. Improvement of the photocatalytic performance compared to the bimolecular reaction is assigned to the conductive connection between the Fe^II^ and Co^III^ centre, which benefits from a directional effect of the bridging ligand.

The reference system based on a noble metal photosensitizer [Ir(ppy)_2_(bpy)]PF_6_ (termed hereafter [Ir]) and [Co] produces 78 μmol H_2_, which corresponds to TONs of 16 (cat) and 31 (PS). Nevertheless, it is obvious that the iron‐based systems, especially the dyad shows moderate activity when compared to the noble metal system. A related Ir−Co‐dyad investigated by Elias et al. showed a TON=224 after irradiation at 452 nm for 35 h. Compared to the dyad, the two‐component system also composed of [Ir] and [Co] only yielded a TON=22 for the photosensitizer under their conditions.^[9d]^ Surprisingly, it decomposed after 0.5 h, whereas in our experiment without an additional proton source in acetonitrile/water mixture [Ir]+[Co] remains active even after 3 h. In another experiment by Elias et al. with 523 nm irradiation [Ir]+[Co] yielded a TON of 16 for the photosensitizer.^[9e]^ Decomposition only took place after four hours. It underlines the difficulty to compare the activity in photocatalytic proton reduction experiments conducted with different set‐ups, irradiation wavelengths, and under different solvent conditions. However, trends within one study can be compared. The activity gain by factor 10 for iridium or factor 2 for iron when forming an assembly is much higher with a noble metal photosensitizer. This difference between Fe‐Co and Ir‐Co dyads is assigned to the lifetime of the relevant photocatalytically active excited state, which is 390 ns in the case of [Ir] and 17.1 ps for [Fe BL] applied here.^[2a]^ It is, therefore, an important aim to increase the lifetime of the used iron photosensitizers to achieve competitive activities. It is, on the other hand, encouraging that a catalytic activity could be observed with such a small lifetime of the excited state and proves the validity of the base metal dyad approach.

## Conclusion

A new base metal iron(II)‐cobalt(III) dyad [Fe−BL−Co] showed a catalytic activity increased by a factor of two in respect to the equivalent two‐component system [Fe−BL]+[Co]. Using concentration‐dependent NMR and UV/Vis measurements, we quantified the dissociation and formation dynamics of the assembly linked by monodentate coordination of a pyridine moiety to the cobaloxime centre in acetonitrile. [Fe−BL−Co] remains intact to more than 90 % for concentrations larger than 2 mM and is formed in situ in a [Fe−BL]+[Co] mixture. Such effects should always be investigated, as they can influence the outcome of spectroscopic and catalytic experiments and can be a source of severe misinterpretation. Of course, this depends on the selected solvent, and less coordinating options could circumvent dissociation.[^9b^, ^9c^, ^36^] The superior catalytic performance of [Fe−BL−Co] was fully explained by the electronic structure properties deduced from the characterization of the ground and excited states. XRD and EXAFS studies yield identical structural parameters at the Fe^II^ centre in both [Fe−BL] and [Fe−BL−Co]. The experimental +II oxidation state at the iron centre was confirmed by XANES. In contrast, the electronic structures of [Fe−BL] and [Fe−BL−Co] exhibit distinct differences. A shoulder at 440 nm in the optical absorption is a unique spectral feature of [Fe−BL−Co], denoting an electronic interaction between the iron and cobalt centres. An additional redshift of the Fe^II^ MLCT absorption bands in the dyad TA spectra, compared to the constituting iron photosensitizer, reflects a reduced HOMO‐LUMO gap due to MLCT state stabilization, which is also reproduced by theoretical calculations. This positive effect of the assembly is also reflected in the excited state behaviour. The photocatalytically active ^3^MLCT state lifetime of [Fe−BL−Co] is slightly increased by roughly 3 ps when compared to [Fe−BL]. Because the MLCT elongation itself would be too small to cause a detectable rise in the photocatalytic performance in a bimolecular reaction, the increased activity of [Fe−BL−Co] is assigned to a directional effect of the conductive linker connection between the Fe^II^ and Co^III^ centres. As such, it demonstrates the potential of designing active base metal assemblies for photocatalytic proton reduction reactions.

Overall, it remains mandatory to increase the relevant MLCT state lifetime while maintaining chemical robustness to further establish base metal assemblies for photocatalytic proton reduction based on iron photosensitizers. Therefore, new ligand types at the iron centre should be explored in dyads to achieve high activity competitive with noble metal systems.^[37]^

## Experimental Section

**Synthesis**. Standard Schlenk technique was applied to carry out the reactions under argon or to degas dry solvents before dried in an MBraun Solvent Purification System. All chemicals were purchased from TCI, Fisher Scientific, Abcr or Sigma–Aldrich. Bruker Ascent 700 or Avance 500 were used to record NMR spectra and a Waters Synapt G2 quadrupole – time‐of‐flight spectrometer to record mass spectra (ESI). The NMR spectra were referenced to the residual of the non‐deuterated solvent signal. L1 was synthesised according to Danopoulos et al.[^15^, ^38^] Synthesis of 4‐(Bpin)‐py‐Cl_2_ was described elsewhere.^[14]^ [Co] was gained by following a protocol by Schrauzer et al.^[16]^

*Synthesis of 4,4’‐bpy‐Cl_2_*. The literature protocol was changed to increase the yield (41 %).^[39]^ 4‐(Bpin)‐py‐Cl_2_ (11 mmol, 3.01 g), 4‐iodo‐pyridine (10 mmol, 2.05 g), K_2_CO_3_ (22 mmol, 3.04 g) were degassed. Degassed solvent mixture THF/H_2_O (95 : 5, 100 mL) was added. In a second flask Pd(OAc)_2_ (0.5 mmol, 112 mg) and SPhos (1 mmol, 411 mg) were mixed for 1 h in THF/H_2_O (30 mL). The catalyst solution was transferred to the reactants, and the resulting mixture was stirred at 60 °C over four days. THF was removed and the aqueous phase was extracted with EtOAc. Column chromatography on silica using cyclohexane/EtOAc (4 : 1) gave 4,4’‐bpy‐Cl_2_ in moderate yield (1.3 g, 58 %). ^1^H NMR (500 MHz, CDCl_3_): *δ*=8.77 (m, 2H), 7.49 (s, 2H), 7.48 (m, 2H) ppm. ^13^C NMR (126 MHz, CDCl_3_): *δ*=152.04, 151.53, 151.41, 143.56, 121.69, 121.22 ppm. ESI‐MS (*m*/*z*) 224.9984 [*M*+H]^+^ (calc. for (C_10_H_7_N_2_Cl_2_)^+^ 224.9986).

*Synthesis of BL‐Cl_2_*. 4,4’‐bpy‐Cl_2_ (5 mmol, 1.13 g) and *N*‐methylimidazole (25 mmol, 2 mL) were mixed at 150 °C for five days. The raw product was dissolved in MeOH, and this solution was added dropwise to acetone. The precipitation was washed with acetone and ether. 1.73 g of BL‐Cl_2_ were isolated (4.4 mmol, 88 %). ^1^H NMR (500 MHz, [D_6_]DMSO): *δ*=10.77 (s, 2H), 8.94‐8.92 (m, 4H), 8.75 (s, 2H), 8.16 (m, *J*=6.9,1.8 Hz, 2H), 8.09 (dd, 2H), 4.06 (s, 6H) ppm. ^13^C NMR (126 MHz, [D_6_]DMSO): *δ*=152.73, 150.96, 146.23, 141.83, 136.72, 125.01, 121.60, 119.31, 111.69, 36.59 ppm. ESI‐MS (*m*/*z*) 159.0796 [*M*]^2+^ (calcd. for (C_18_H_18_N_6_)^2+^ 318.1593/2=159.0797).

*Synthesis of [Fe−BL]*. L1 (0.5 mmol, 347 mg) was cooled to −70 °C in dry, degassed THF (25 mL). Fe(HMDS)_2_ (1 mmol, 188 mg) was dissolved in THF (5 mL) and added to L1. The mixture was warmed to room temperature overnight. Parallel, BL‐Cl_2_ (0.5 mmol, 195 mg) was deprotonated with LiHMDS (1.5 mmol, 1 M in THF, 1.5 mL) in THF (25 mL) at −20 °C and warmed to room temperature overnight. Both solutions were combined and stirred for 1 h. After removal of THF and dissolving in water the aqueous phase was filtrated. KPF_6_ (4 mmol, 736 mg) dissolved in water was added. An orange precipitation was collected, washed with water and dissolved in acetone. After alumina column chromatography with MeCN/Et_2_O (1 : 1) [Fe−BL] was gained as orange powder (296 mg, 50 %). ^1^H NMR (500 MHz, [D_6_]acetone): *δ*=8.83 (m, *J*=4.3, 1.7 Hz, 2H), 8.72 (d, *J*=2.2 Hz, 2H), 8.55 (t, 1H), 8.45 (d, *J*=2.1 Hz, 2H), 8.35 (d, *J*=8.2 Hz, 2H), 7.70 (s, 2H), 7.63 (dd, *J*=4.3, 1.7 Hz, 2H), 7.60 (d, *J*=2.2 Hz, 2H), 7.46 (d, *J*=2.1 Hz, 2H), 7.04 (t, *J*=7.8 Hz, 2H), 6.88 (d, *J*=7.8 Hz, 4H), 2.99 (s, 6H), 1.47 (hept, *J*=6.6 Hz, 4H), 1.01 (d, *J*=6.6 Hz, 12H), 0.78 (d, *J*=6.7 Hz, 12H) ppm. ^13^C NMR (126 MHz, [D_6_]acetone): *δ*=201.56, 201.15, 155.02, 154.77, 151.94, 149.10, 146.15, 145.00, 140.13, 134.97, 131.72, 130.80, 127.44, 124.38, 122.52, 120.06, 117.17, 107.51, 103.33, 35.73, 28.80, 27.14, 24.38 ppm. ESI‐MS (*m*/*z*) 451.7065 [*M*]^2+^ (calcd. for [C_53_H_57_N_11_Fe]^2+^ 903.4148/2=451.7072). elemental analysis calcd (%) for C_53_H_57_N_11_FeP_2_F_12_: C 53.32, H 4.81, N 12.91; found: C 52.93, H 4.94, N 12.57.

*Synthesis of [Fe−BL−Co]*. CoCl_2_ ⋅ 6 H_2_O (0.042 mmol, 10 mg) and dimethylglyoxime (0.088 mmol, 10.2 mg) were dissolved in ethanol (95 %, 5 mL) and heated to 70 °C. [Fe−BL] (0.04 mmol, 47.8 mg) dissolved in EtOH was added and the reaction mixture was cooled to room temperature. A stream of air was passed through the solution for 60 min causing a red solid to precipitate. Then Et_2_O (5 mL) was added and the raw product was isolated, washed with Et_2_O and EtOH. Drying gave [Fe−BL−Co] as red powder (46.2 mg, 76 %). ^1^H NMR (700 MHz, [D_6_]acetone): *δ*=8.74 (d, *J*=2.3 Hz, 2H), 8.58 (t, *J*=8.2 Hz, 1H), 8.42 (m, 2H), 8.39 (d, *J*=2.1 Hz, 2H), 8.36 (d, *J*=8.2 Hz, 2H), 7.67 (m, 2H), 7.62 (s, 1H), 7.59 (d, *J*=2.2 Hz, 2H), 7.47 (d, *J*=2.1 Hz, 2H), 6.97 (t, *J*=7.8 Hz, 2H), 6.85 (d, *J*=7.8 Hz, 4H), 3.00 (s, 6H), 2.43 (s, 12H), 1.46 – 1.39 (m, 4H), 0.97 (d, *J*=6.6 Hz, 12H), 0.75 (d, *J*=6.6 Hz, 12H) ppm. ^13^C NMR (176 MHz, [D_6_]acetone): *δ*=200.98, 200.87, 154.91, 154.81, 153.38, 152.60, 148.65, 146.26, 146.08, 140.40, 134.85, 131.68, 130.69, 127.54, 124.97, 124.37, 120.09, 117.27, 107.62, 103.34, 35.75, 28.73, 27.11, 24.44, 13.04 ppm. ESI‐MS (*m*/*z*) 613.7103 [*M*]^2+^ (calcd. for [C_61_H_71_N_15_FeCoClO_4_]^2+^ 1227.4183/2=613.7092), 1372.3896 [*M*+PF_6_]^+^ (calcd. for [C_61_H_71_N_15_FeCoClO_4_PF_6_]^+^ 1372.3825). elemental analysis calcd (%) for C_61_H_71_N_15_FeCoO_4_ClP_2_F_12_: C 48.25, H 4.71, N 13.84; found: C 47.73, H 4.97, N 13.66.

**X‐ray crystallography**. X‐ray single‐crystal data of [BL‐Cl_2_] and [Fe−BL] were recorded using a Bruker Venture D8 diffractometer applied with a Mo_Kα_ μ‐source (*λ*=0.71073 Å) and a Photon III area detector at 120 K. Single crystal data of [Fe−BL−Co] was collected using a Bruker Smart Apex II Quasar with an Incoatec Mo IμS Source (*λ*=0.71073 Å) and an Apex II area detector at 100 K. The data were integrated with SAINT. A multi‐scan absorption correction was applied using SADABS.^[40]^ Structure solution was achieved by direct methods in SHELXT^[41]^, and structure refinement was conducted using full‐matrix least‐squares refinement based on *F* ^2^.^[42]^ All non‐hydrogen‐atoms were refined anisotropically, and the hydrogen atom positions were refined at idealized positions riding on the carbon atoms with isotropic displacement parameters *U*_iso_(H)=1.2 *U*_eq_(C) or 1.5 *U*_eq_(‐CH_3_) and C−H bond lengths of 0.93‐0.96 Å. The torsion angles of the methyl groups were refined. Hydrogen atoms connected to oxygen atoms were refined freely with a distance restraint. In the case of [BL‐Cl_2_] one methanol molecule, in [Fe−BL] two acetonitrile molecules, and in [Fe−BL−Co], several ethanol molecules could not be modelled due to significant disorders and therefore were treated using SQUEEZE from the Platon software package.^[43]^

Deposition numbers 2053791 (for [BL‐Cl_2_]), 2053792 (for [Fe−BL]) and 2049533 (for [Fe−BL−Co]) contain the supplementary crystallographic data for this paper. These data are provided free of charge by the joint Cambridge Crystallographic Data Centre and Fachinformationszentrum Karlsruhe Access Structures service.

**DFT calculations**. The ORCA 4.0.1 quantum chemistry package was applied for all calculations.^[44]^ The PBEh‐3c method was used for all unconstrained DFT optimisations of all investigated complexes.^[45]^ All single‐point calculations were performed using TPSSh^[46]^ hybrid functional together with the Alrichs def2‐TZVPP^[47]^ basis set on all atoms with the inclusion of MeCN solvation via SMD.^[48]^ Correction for dispersion interaction was done by DFT‐D3 with Becke‐Johnson damping scheme (D3BJ).^[49]^ The frontier orbitals of both investigated complexes are depicted in Figures S24 and S25.

**UV/Vis spectroscopy**. UV/Vis spectra with 1 ⋅ 10^−5^ mol/L were recorded on a Lambda 45 double‐beam UV spectrophotometer from PerkinElmer using 1 cm Quartz cuvettes. Solutions up to 2.5 ⋅ 10^−4^ mol/L were measured on a Lambda 465 spectrophotometer in a 0.1 cm quartz cuvette. Spectroscopy grade acetonitrile was used for all measurements.

**Electrochemistry**. Spectro‐electrochemical experiments were performed in an optically transparent electrochemical cell (*d*=1 mm, MeCN/0.1 M (*n*Bu)_4_NPF_6_ as supporting electrolyte) with a Pt gauze working electrode at room temperature. Spectral changes upon oxidation or reduction were recorded on a Varian Cary 50 spectrophotometer covering the range of 200‐1100 nm. All measurements were carried out under argon, with absolute and deoxygenated acetonitrile. Cyclic and square‐wave voltammograms at room temperature were performed with the PAR101 potentiostat from Metrohm or the Compactstat from Ivium in MeCN/0.1 M (*n*Bu)_4_NPF_6_ (*c*(analyt)=0.001 mol/L) with the following three‐electrode arrangement: Pt working electrode (1 mm diameter) or glassy carbon working electrode (2 mm diameter), Ag/0.01 M AgNO_3_, 0.1 M (*n*Bu)_4_NPF_6_ in MeCN as the reference electrode. Pt wire was used as the counter electrode. As an internal standard, ferrocene was used after the measurements, and all potentials are referenced with respect to the Fc/Fc^+^ couple. The cyclic and square‐wave voltammograms were analysed with the software NOVA version 2.1.3 from Metrohm based on the diagnostic criteria proposed by Nicholson^[50]^ and the Randles–Sevcik equation.^[51]^

**Optical transient absorption**: The details of the experimental setup used in this study were described in detail elsewhere.^[52]^ Ultrafast excited state dynamics of [Fe−BL] and [Fe−BL−Co] were recorded by a commercial femtosecond transient absorbance setup (Helios spectrometer, Ultrafast Systems) having instrument response function (IRF) of 120 fs. Unlike the original commercial setup, a dichroic optical filter (placed before the sample flow cell) was used, which masks the residual 800 nm white light continuum. The setup also included a continuously moving CaF_2_ crystal required for white light continuum generation in 330–720 nm spectral range. Femtosecond transient absorption spectra were recorded under 515 nm excitation in 450 ps timescale using 2 μJ pump energy. Due to a high absorbance of the chromophores in 10 mM or 5 mM solutions in MeCN, a 0.12 μm flow cell with CaF_2_ windows was used to ensure optimised signal transmission. The flow cell was attached to a micro annular gear pump providing ∼1 mL/s flow to guarantee the excitation of fresh solution for each laser pulse and minimum sample degradation. To eliminate solvent contribution to the TA data, subtraction of solvent response from each data set was carried out.

**Associated content**: NMR spectra, computational studies, X‐ray, UV/Vis und pump‐probe measurements, cyclic voltammograms as well as photocatalytic proton reduction experiments are provided in the Supporting Information.

## Conflict of interest

The authors declare no conflict of interest.

## Supporting information

As a service to our authors and readers, this journal provides supporting information supplied by the authors. Such materials are peer reviewed and may be re‐organized for online delivery, but are not copy‐edited or typeset. Technical support issues arising from supporting information (other than missing files) should be addressed to the authors.

Supplementary
