# Supplementary material for: Fundamental Characterization, Photophysics and Photocatalysis of a Base Metal Iron(II)‐Cobalt(III) Dyad
Source: Chemistry. 2021 Jun 1;27(38):9905–18. doi: 10.1002/chem.202100766 (PMC8362051; doi:10.1002/chem.202100766)
Supplement: Supplementary file 1 — Supplementary [file CHEM-27-9905-s001.pdf]

Chemistry–A European Journal

Supporting Information

Fundamental Characterization, Photophysics and Photocatalysis of a Base Metal Iron(II)-Cobalt(III) Dyad

Marina Huber-Gedert, Michał Nowakowski, Ahmet Kertmen, Lukas Burkhardt, Natalia Lindner, Roland Schoch, Regine Herbst-Irmer, Adam Neuba, Lennart Schmitz, Tae-Kyu Choi, Jacek Kubicki, Wojciech Gawelda, and Matthias Bauer*

Table of Contents

1. Details of single crystal structure analysis 2
2. XAS spectroscopy 6
3. NMR spectroscopy 10
   3.1 Dyad dissociation study 10
   3.2 Dyad association study 11
   3.3 Photostability study 13
4. UV-Vis spectroscopy 15
5. Electrochemistry 16
6. Quantum chemical calculations 21
7. Optical transient absorption spectroscopy 28
8. Photocatalytic proton reduction 33
9. Spectra 35

References 41

1. Details of single crystal structure analysis

[BL-Cl_2]

($C_{18}H_{18}N_6$) 2Cl, M_r = 389.28 Da, blue needle, size: 0.28 x 0.10 x 0.08 mm³, monoclinic space group C2/c with Z = 4, a = 20.2666(7) Å, b = 15.6822(5) Å, c = 7.2421(3) Å, β = 107.2860(10)°, V = 2197.76(14) Å³, D_c = 1.177 mg/m³, μ = 0.308 mm^{-1}, F(000) = 808, 2.105° ≤ θ ≤ 26.395°, reflections collected: 26438, independent reflections: 2248, R_{int} = 0.0421, refinement converged at R1 = 0.0306 [I>2σ(I)], wR2 = 0.0873 [all data], min./max. ΔF: -0.25 eÅ^{-3} (1.01 Å from N2) / 0.23 eÅ^{-3} (0.65 Å from C5), **CCDC-No.: 2053791**

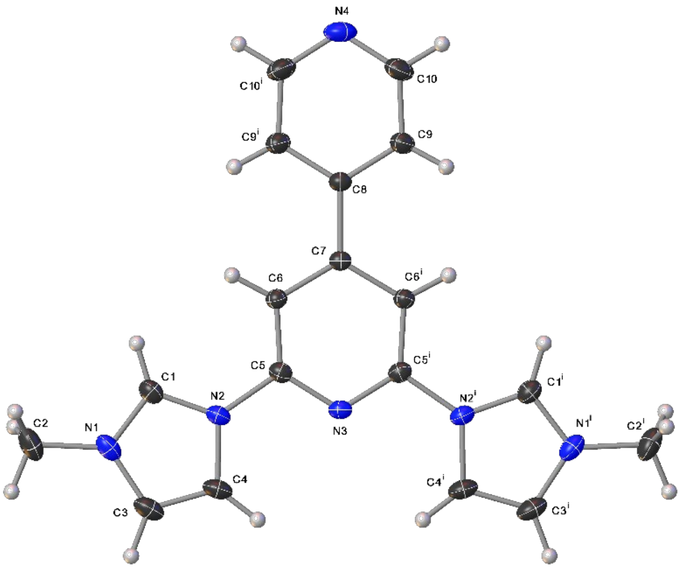

Figure S1: Molecular structure of BL-Cl_2.Anions and co-crystalized solvent molecules are omitted for clarity. Anisotropic displacement ellipsoids are drawn at the 50% probability level.

Table S1. Crystal data and structure refinement for [BL-Cl_2].

CCDC code	2053791	
Empirical formula	C18 H18 Cl2 N6	
Formula weight	389.28	
Temperature	120(2) K	
Wavelength	0.71073 Å	
Crystal system	Monoclinic	
Space group	C2/c	
Unit cell dimensions	a = 20.2666(7) Å	α= 90°.
	b = 15.6822(5) Å	β= 107.2860(10)°.
	c = 7.2421(3) Å	γ = 90°.
Volume	2197.76(14) Å^3	
Z	4	
Density (calculated)	1.177 Mg/m^3	
Absorption coefficient	0.308 mm^{-1}	
F(000)	808	
Crystal size	0.280 x 0.100 x 0.080 mm^3	
Theta range for data collection	2.105 to 26.395°.	
Index ranges	-25<=h<=25, -19<=k<=19, -9<=l<=7	
Reflections collected	26438	

Independent reflections	2248 [R(int) = 0.0421]
Completeness to theta = 25.242°	99.7 %
Absorption correction	Semi-empirical from equivalents
Refinement method	Full-matrix least-squares on F^2
Data / restraints / parameters	2248 / 0 / 121
Goodness-of-fit on F^2	1.056
Final R indices [I>2sigma(I)]	R1 = 0.0306, wR2 = 0.0840
R indices (all data)	R1 = 0.0346, wR2 = 0.0872
Extinction coefficient	n/a
Largest diff. peak and hole	0.229 and -0.252 e.Å^{-3}

[Fe-BL]

$(C_{55}H_{60}N_{12}Fe)$ 2(PF_6), M_r = 1234.94 Da, red block, size: 0.274 x 0.239 x 0.196 mm³, monoclinic space group C2/c with Z = 8, a = 41.299(11) Å, b = 15.151(3) Å, c = 22.208(7) Å, β = 121.966(9)°, V = 11789(5) Å³, D_c = 1.392 mg/m³, μ = 0.396 mm^{-1}, F(000) = 5104, 2.325° ≤ θ ≤ 27.433°, reflections collected: 74175, independent reflections: 13445, R_{int} = 0.0348, refinement converged at R1 = 0.0382 [I>2σ(I)], wR2 = 0.1040 [all data], min./max. ΔF: -0.66 eÅ^{-3} (0.54 Å from P2) / 0.57 eÅ^{-3} (0.91 Å from F14), **CCDC-No.: 2053792**

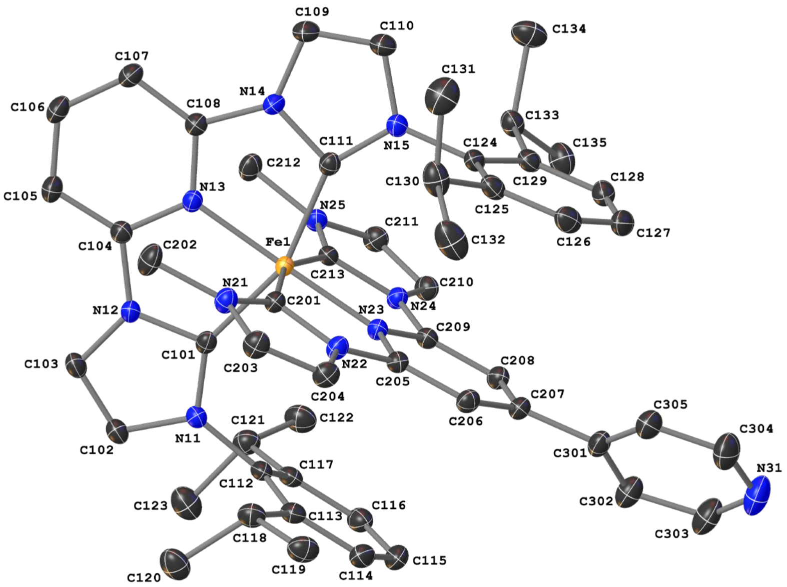

Figure S2: Molecular structure of Fe-BL. Anions and hydrogen atoms are omitted for clarity. Anisotropic displacement ellipsoids are drawn at the 50% probability level.

Table S2: Crystal data and structure refinement for [Fe-BL].

CCDC code	2053792	
Empirical formula	C55 H60 F12 Fe N12 P2	
Formula weight	1234.94	
Temperature	100(2) K	
Wavelength	0.71073 Å	
Crystal system	Monoclinic	
Space group	C2/c	
Unit cell dimensions	a = 41.299(11) Å	α= 90°.
	b = 15.151(3) Å	β= 121.966(9)°.
	c = 22.208(7) Å	γ = 90°.

Volume	11789(5) Å^3
Z	8
Density (calculated)	1.392 Mg/m^3
Absorption coefficient	0.396 mm^{-1}
F(000)	5104
Crystal size	0.274 x 0.239 x 0.196 mm^3
Theta range for data collection	2.325 to 27.433°.
Index ranges	-53<=h<=53, -19<=k<=19, -28<=l<=28
Reflections collected	74175
Independent reflections	13445 [R(int) = 0.0348]
Completeness to theta = 25.242°	99.9 %
Absorption correction	Semi-empirical from equivalents
Refinement method	Full-matrix least-squares on F^2
Data / restraints / parameters	13445 / 0 / 750
Goodness-of-fit on F^2	1.020
Final R indices [I>2sigma(I)]	R1 = 0.0382, wR2 = 0.0973
R indices (all data)	R1 = 0.0468, wR2 = 0.1040
Extinction coefficient	n/a
Largest diff. peak and hole	0.570 and -0.657 e.Å^{-3}

[Fe-BL-Co]

($C_{61}H_{71}N_{15}O_4ClCoFe$) 2($PF_6$), M_r = 1518.49 Da, orange block, size: 0.724 x 0.334 x 0.316 mm³, orthorhombic space group $P2_12_12_1$ with Z = 4, a = 14.673(2) Å, b = 19.008(3) Å, c = 29.325(3) Å, V = 8178.9(19) Å³, D_c = 1.233 Mg/m³, μ = 0.527 mm^{-1}, F(000) = 3128, 1.277° ≤ θ ≤ 27.918°, reflections collected: 300927, independent reflections: 19528, R_{int} = 0.0331, refinement converged at R1 = 0.0345 [I>2σ(I)], wR2 = 0.0897 [all data], min./max. ΔF: -0.53 eÅ^{-3} (0.74 Å from Cl1) / 0.42 eÅ^{-3} (0.73 Å from Cl1), **CCDC-No.:** 2049533

Table S3. Crystal data and structure refinement for [Fe-BL-Co].

CCDC code	2049533
Empirical formula	C61 H71 Cl Co F12 Fe N15 O4 P2
Formula weight	1518.49
Temperature	100(2) K
Wavelength	0.71073 Å
Crystal system	Orthorhombic
Space group	$P2_12_12_1$

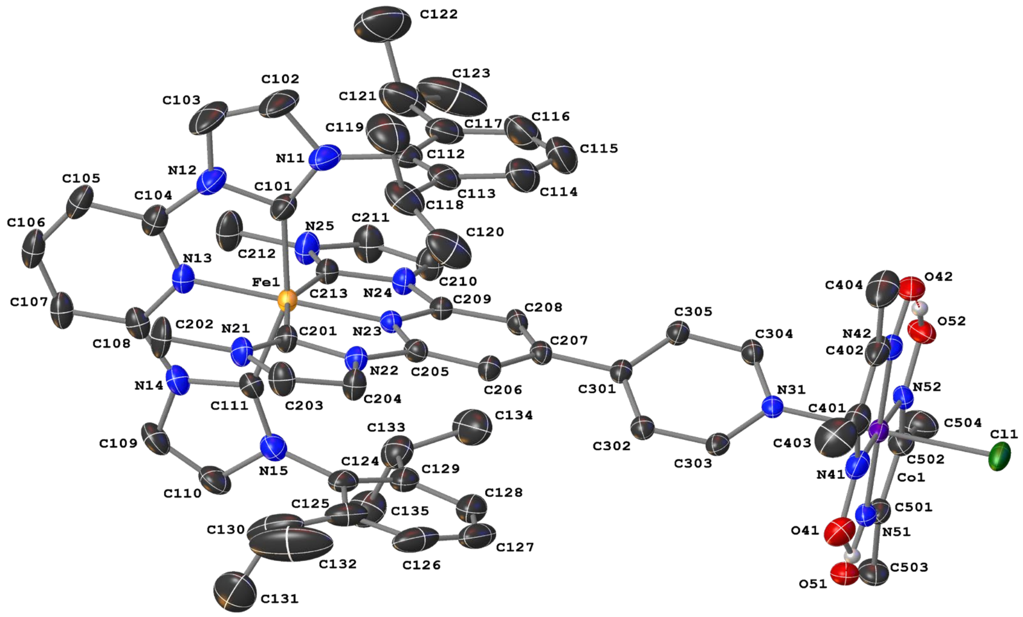

Figure S3: Molecular structure of Fe-BL-Co. Anions and hydrogen atoms (except in glyoxime moiety) are omitted for clarity. Anisotropic displacement ellipsoids are drawn at the 50% probability level.

Unit cell dimensions	a = 14.673(2) Å	α= 90°.
	b = 19.008(3) Å	β= 90°.
	c = 29.325(3) Å	γ = 90°.
Volume	8178.9(19) Å^3	
Z	4	
Density (calculated)	1.233 Mg/m^3	
Absorption coefficient	0.527 mm^{-1}	
F(000)	3128	
Crystal size	0.724 x 0.334 x 0.316 mm^3	
Theta range for data collection	1.277 to 27.918°.	
Index ranges	-19<=h<=19, -24<=k<=24, -38<=l<=38	
Reflections collected	300927	
Independent reflections	19528 [R(int) = 0.0331]	
Completeness to theta = 25.242°	100.0 %	
Absorption correction	Semi-empirical from equivalents	
Max. and min. transmission	0.4308 and 0.3525	
Refinement method	Full-matrix least-squares on F^2	
Data / restraints / parameters	19528 / 508 / 960	
Goodness-of-fit on F^2	1.031	
Final R indices [I>2sigma(I)]	R1 = 0.0345, wR2 = 0.0872	
R indices (all data)	R1 = 0.0381, wR2 = 0.0897	
Absolute structure parameter	0.0153(19)	
Extinction coefficient	n/a	
Largest diff. peak and hole	0.528 and -0.419 e.Å^{-3}	

2. XAS spectroscopy

XANES and EXAFS measurements. The EXAFS measurements were conducted at the P65 beamline, DESY, Hamburg. Energy selection was done with the use of the Si(111) Double Crystal Monochromator (DCM) and photon flux on the sample was approximately 10^{11} ph/s. The experiment was performed in Total Fluorescence Mode with the use of the PIPS detector in 45° geometry. Energy calibration was set to the first inflection point in Fe foil XANES spectrum with an energy of 7112.1 eV. Samples were in form of powder pressed into boron nitride pellet and data collection was performed at room temperature. Data reduction, normalization, and EXAFS fitting were performed in the Demeter package.[1] Prior to the main analysis an EXAFS analysis of the Fe foil was conducted in order to obtain SO2 value which is listed in Table S5. The RBKG parameter for all compounds was set to 1.1. Background reduction and Fourier Transform parameters were set in the range of 2.5-11.5 $Å^{-1}$ and 1.1-4.0 Å. The SO2 parameter of 0.93(1) was obtained by EXAFS analysis of reference Fe foil and further was kept fixed on this value. The analysis was conducted for k range of 2.5-11.5 $Å^{-1}$ because in [Fe-BL-Co] at an energy of about 7650 eV a pre-edge feature of Co K-edge started to emerge. No significant correlations were observed within the fitted parameters and all obtained values were statistically consistent.

The XANES experiment was conducted at P64 beamline. Energy selection was done with the use of the Si(111) Double Crystal Monochromator (DCM) and photon flux on the sample was approximately 10^{12} ph/s. The experiment was performed in Total Fluorescence Mode with the use of the PIPS detector in 45° geometry. Energy calibration was set to the first inflection point in Fe foil XANES spectrum with the energy of 7112.0 eV. To avoid radiation damage measurements were taken in a liquid jet at room temperature. Spectra background reduction and normalization were conducted in the Demeter package, while *ab-initio* calculations were conducted in FEFF software.[2,3] The unoccupied contributions to DOS functions were extracted with the use of the arctangent step function.[4,5]

XANES analysis and FEFF calculations

Fe K-edge XANES spectra of [Fe-BL] and [Fe-BL-Co] with respect to Fe^0 are presented in Figure S4a. The spectra of [Fe-BL] and [Fe-BL-Co] are very similar with only a slight redshift of the unsymmetrical first white line feature by around 1 eV. According to comparison with well-known references with highly defined oxidation states, the iron centers in [Fe-BL] and [Fe-BL-Co] are numerically determined to be in a +1.7 oxidation state in agreement with Fe^{II} (Figure S4c). This conclusion is supported by the single pre-peak feature around 7114 eV, together with a

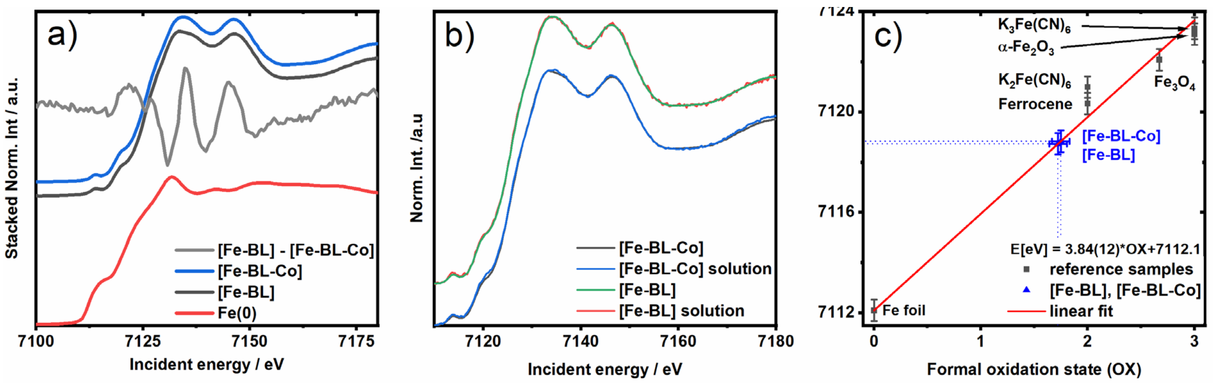

Figure S4: a) XANES spectra of [Fe-BL] (dark grey) and [Fe-BL-Co] (blue) compared to Fe^0 (red). Light grey line shows differential spectrum of [Fe-BL] - [Fe-BL-Co]. b) Comparison between solid and solution XAS spectra for [Fe-BL] and [Fe-BL-Co] c) Formal oxidation state of [Fe-BL] and [Fe-BL-Co] derived from absorption edge position.

well-pronounced near-edge shoulder around 7120 eV that are well-known features in Fe^{II} tetra-NHC complexes.[6] Both complexes show a well-pronounced double-peak white-line feature, which is known for similar complexes coordinated by a 4,4'-bipyridine bridging ligand motif.[7] The identical energy position and intensity of the pre-edge and near-edge is indicating only negligible changes in the electronic structure around the iron atom and ligand. FEFF calculations conducted for crystal structures of [Fe-BL] and [Fe-BL-Co] show, that the 7114 eV pre-edge peak arises due to quadrupole-like transitions allowed by unoccupied Fe 3d-states mixing with a very small fraction of N p-states.[8,9] Contrary to that, a pre-peak at 6-7 eV is an overlap of pre-edge peaks which raised due to strong mixing of Fe dDOS with p-states both from C and N (Figures S5). According to a standard electronic energy level distribution of a distorted octahedral symmetry of the Fe^{II} center (formally $3d^6$) the presence of two dDOS peaks in the pre-edge region indicates not fully occupied t_{2g} states and mostly empty e_g states. This effect can be induced by changes in a degeneration scheme of the d-states by the significantly distorted ligand field. Moreover, the e_g states are involved in bond creation as they strongly overlap with ligand p-states. There is also no significant difference when compared XAS spectra of [Fe-BL] and [Fe-BL-Co] in solid and solution which indicates marginal solvation effects in MeCN (*cf.* Figure S4b).

In [Fe-BL] 3d DOS there is a peak at -1 eV – it is from residual occupied 3d states. Its relative position to the first pre-edge peak is ~2.7 eV and can be interpreted as a value of bandgap of the compound.[10] Analogically for the [Fe-BL-Co], the bandgap was of 2.4 eV, as the residual occupied 3d peak is at Fermi level, while the centre of mass for the first pre peak is at 2.4 eV. The small differences in white line shape can be explained by a slightly different contribution of ligand p-states to Fe pDOS function as well as a subsequent change of empty Fe p-states.

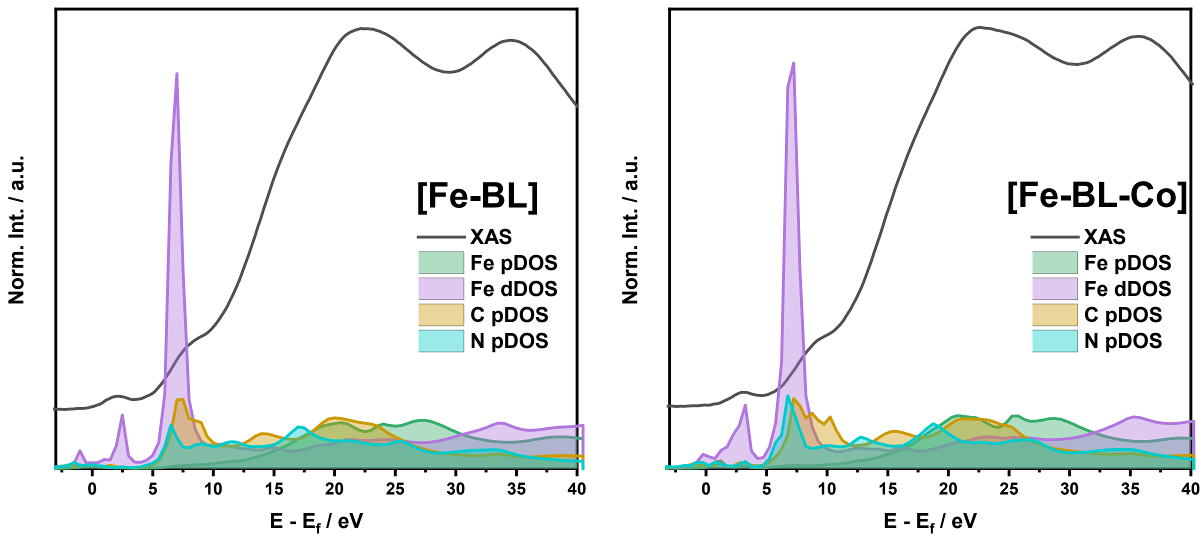

Figure S5: DOS functions calculated using Full Multiple Scattering theory and crystal structures compared to the XANES data for: left) [Fe-BL], right) [Fe-BL-Co]. Energy scale is relative to the Fermi level.

EXAFS analysis

An Extended X-Ray Absorption Spectroscopy (EXAFS) analysis based on the XRD structures as the initial model was conducted at the iron edge of both [Fe-BL] and [Fe-BL-Co]. Fits of the EXAFS spectra are presented in Figure S6 and the extracted structural parameters are given in Table S4. Corresponding bond lengths are included in Table 1. Crystal structure analysis showed that both [Fe-BL] and [Fe-BL-Co] are characterized by similar Fe-ligand bond length and angles in the solid. The spatial resolution of EXAFS is limited to approximately 0.01 Å,[11] therefore it serves as an indicator of significant geometric differences between both studied structures. All observed values (coordination number and atomic positions) are equal in the range of uncertainties for both complexes, thus no significant difference is observed in the coordination sphere of both Fe centres. It is worth to mention, that all fitted values, which include atomic distances, are averaged over 4 C and 2 N atoms (*cf*. Table 1). EXAFS-derived values of Fe-N bond lengths are however different from values obtained in the XRD experiment by a factor of 0.1 Å. On the other hand, the difference between EXAFS Fe-C distance and XRD values is by a factor of 0.01 Å. These discrepancies can be accounted to the differences between the crystal structure environment and powder environment as well as to the lower EXAFS spatial resolution.

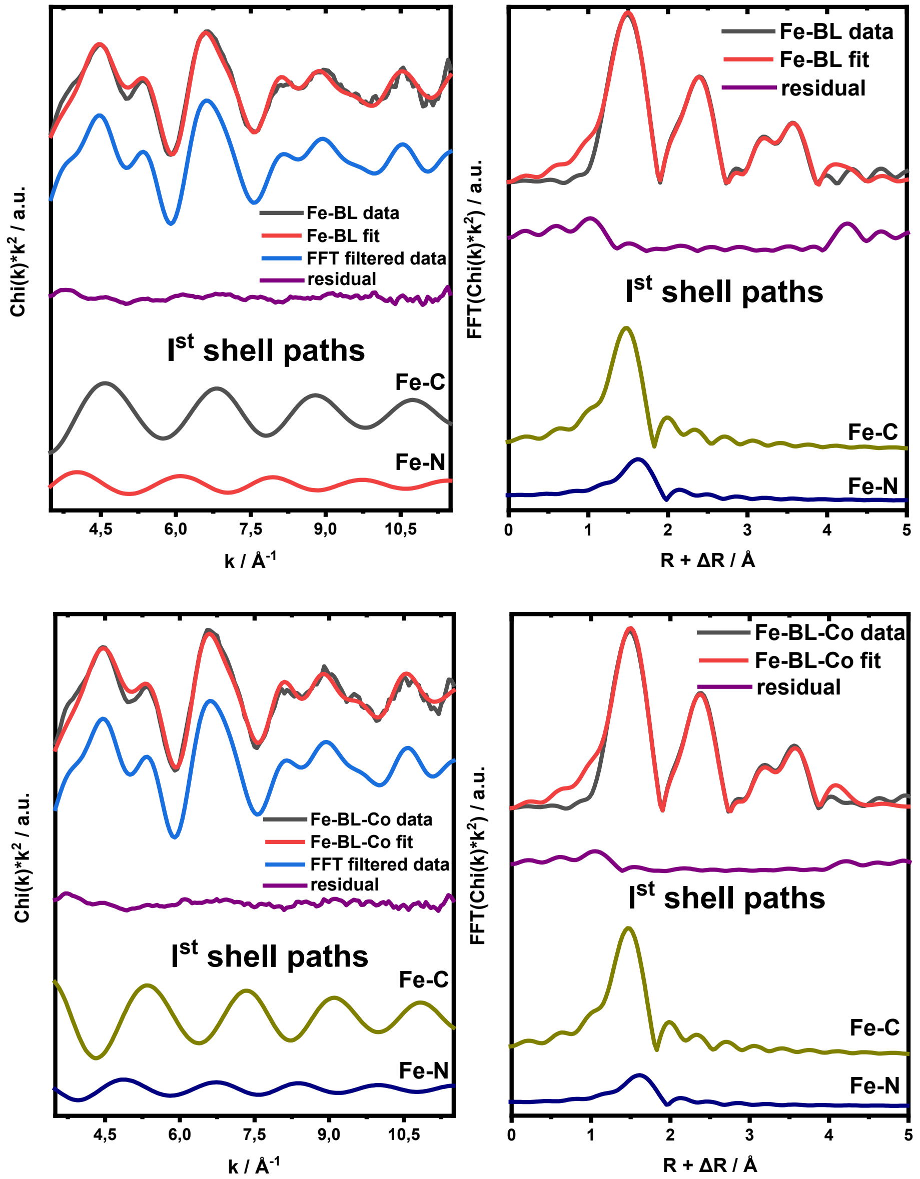

Figure S6: EXAFS fitting results: top) Chi(k)*k^2 and Fourier transform of Chi(k)*k^2 for [Fe-BL]; bottom) Chi(k)*k^2 and Fourier transform of Chi(k)*k^2 for [Fe-BL-Co].

Table S4: EXAFS fitting results.

Compound	Scattering paths	N	R + ΔR [Å]	σ^2 [$Å^2$]
[Fe-BL]	Fe-C	4.6(3)	1.945(8)	0.0013(2)
	Fe-N	1.9(5)	2.112(24)	0.0019(4)
	Fe-N	2.3(3)	2.363(7)	0.0010(4)
	Fe-C	4.8(6)	2.879(11)	0.0009(3)
	Fe-N-C	8.7(1.7)	3.076(21)	0.0019(6)
	Fe-N-C	55.7(8.5)	3.602(15)	0.0029(8)
	Fe-N	3.4(1.2)	3.670(1)	0.0010(4)
	Fe-C	18.0(6.0)	3.975(11)	0.0027(6)
	Fe-C-C	20.5(9.2)	4.118(9)	0.0037(8)
	Fe-C	14.3(2.3)	4.382(27)	0.0027(6)
[Fe-BL-Co]	Fe-C	4.5(3)	1.942(11)	0.0010(3)
	Fe-N	1.8(3)	2.099(51)	0.0015(4)
	Fe-N	2.0(3)	2.375(17)	0.0008(5)
	Fe-C	5.2(6)	2.868(15)	0.0007(4)
	Fe-N-C	7.8(1.2)	3.067(25)	0.0016(7)
	Fe-N-C	60.5(9.5)	3.584(24)	0.0023(9)
	Fe-N	3.3(1.3)	3.637(56)	0.0008(5)
	Fe-C	15.8(4.7)	3.951(44)	0.0022(6)
	Fe-C-C	15.4(5.6)	4.078(64)	0.0030(8)
	Fe-C	15.3(2.7)	4.355(34)	0.0022(6)

a) Adapted from ref.[12]

Table S5: Other EXAFS fitting parameters.

Compound	SO2	red-χ^2	R	ΔE_0 [eV]
[Fe-BL]	0.93(3)	46.78	0.008	2.72(1.11)
[Fe-BL-Co]	0.93(1)	55.28	0.010	2.08(1.23)

a) Adapted from ref.[12]

3. NMR spectroscopy

3.1 Dyad dissociation study

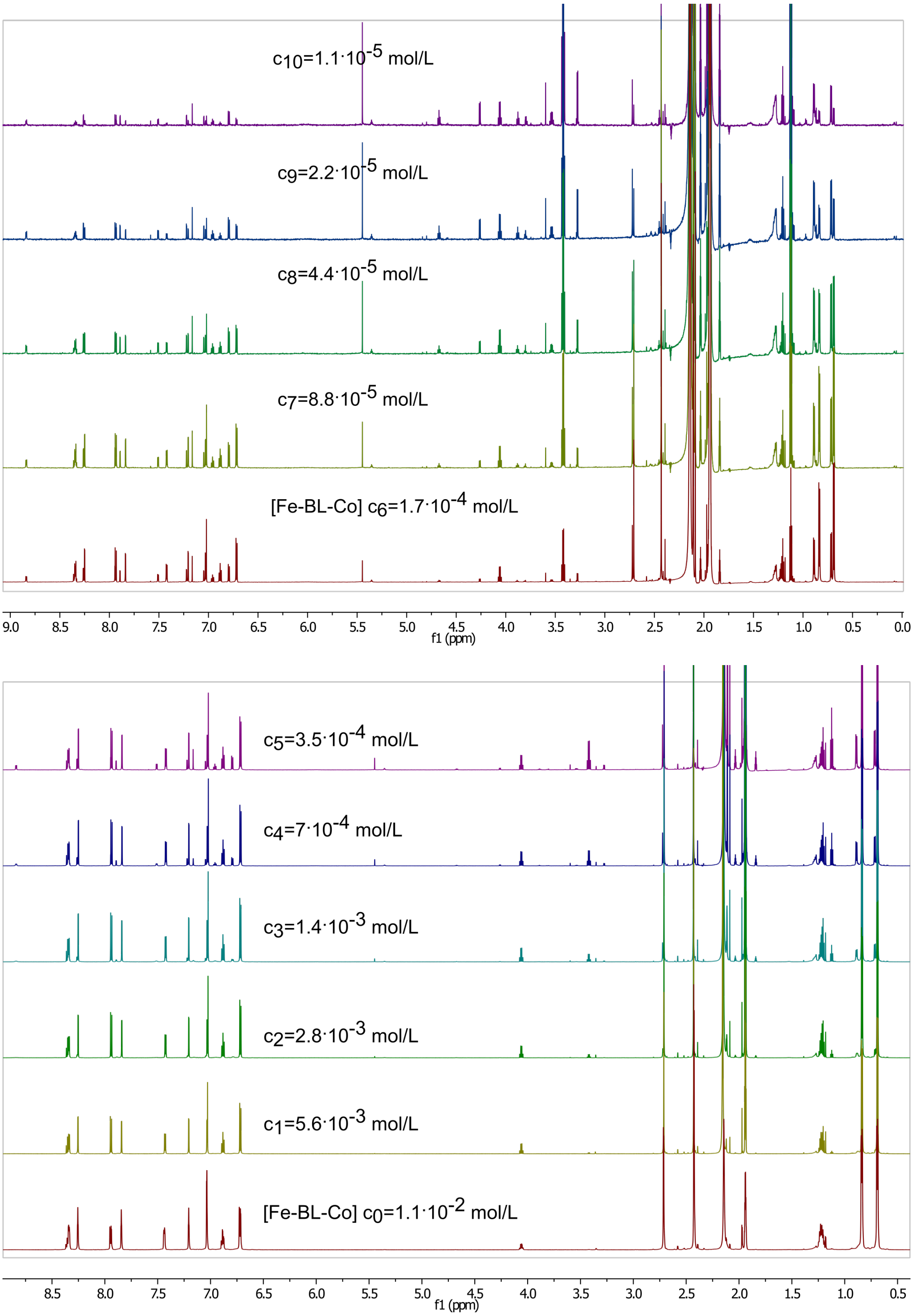

Figure S7: ^{1}H-NMR spectra of [Fe-BL-Co] in acetonitrile-d$_3$; $c_0=1.1\cdot10^{-2}$ mol/L to $c_{10}=1.1\cdot10^{-5}$ mol/L (700 MHz, 298 K).

3.2 Dyad association study

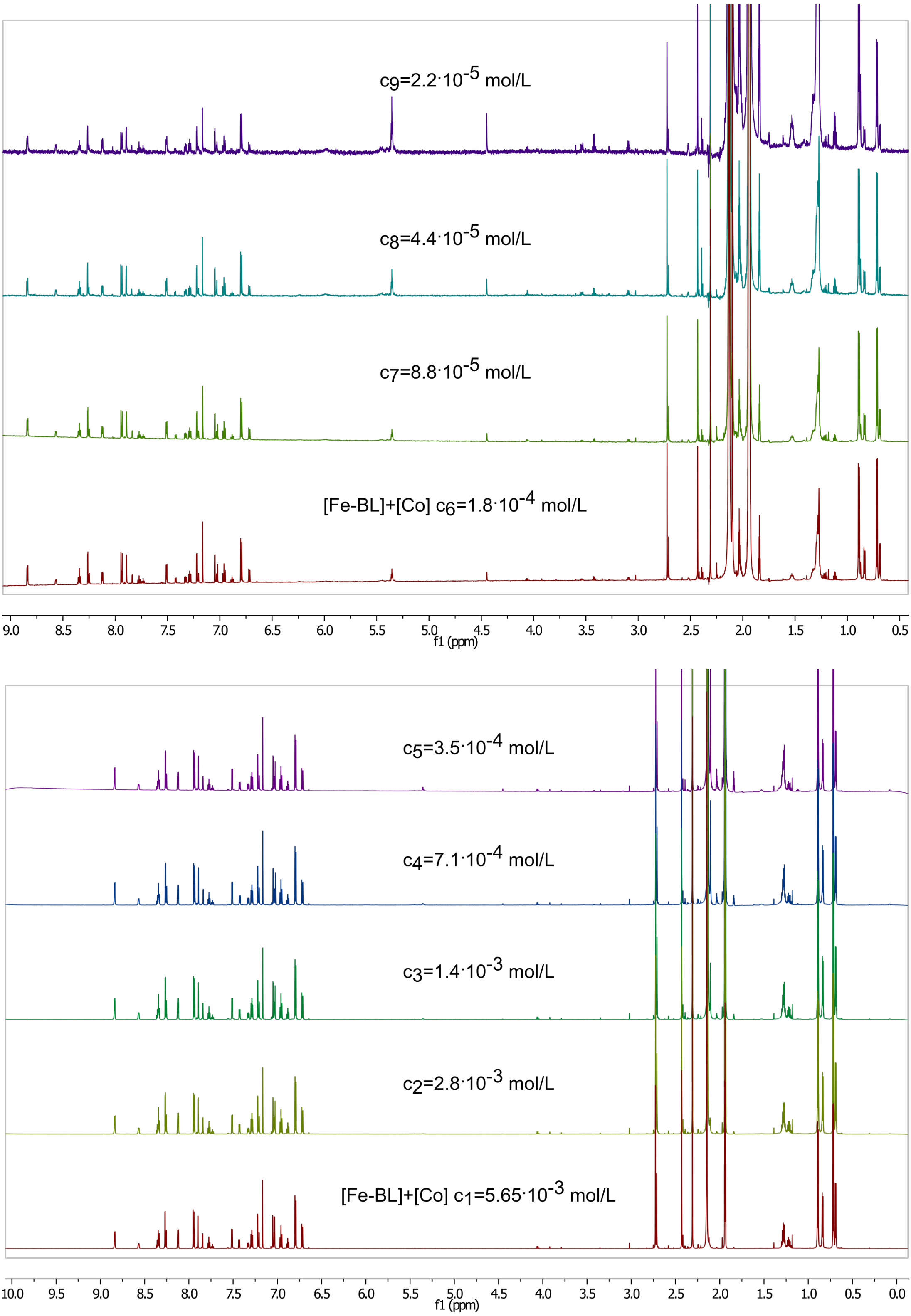

Figure S8: ^{1}H-NMR spectra of 1:1 mixtures of [Fe-BL]+[Co] in acetonitrile-d_3; $c_1=5.65\cdot10^{-3}$ mol/L to $c_9=2.2\cdot10^{-5}$ mol/L (700 MHz, 298 K).

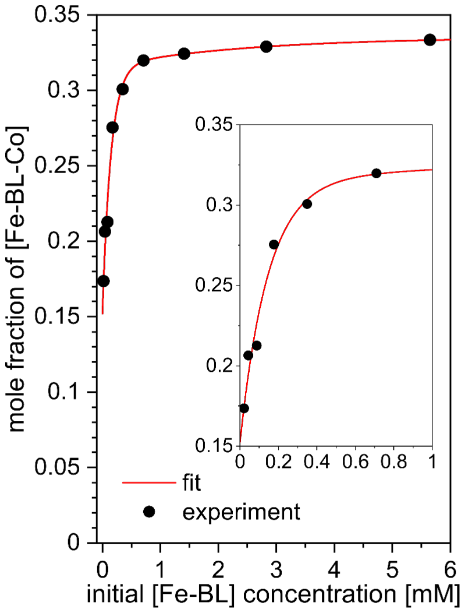

Figure S9: Mole fraction of [Fe-BL-Co] vs. initial [Fe-BL] concentration from 1:1 mixtures of [Fe-BL] and [Co]. Integrals of in-situ formed [Fe-BL-Co] and remaining [Fe-BL] were extracted from the spectra and plotted as a function of the photosensitizer concentration. Mole fraction was calculated from proton integral ratio following equation: $f_{dyad}= I_{dyad}/(I_{dyad}+I_{PS})$. $I_{dyad}+I_{PS}$ is proportional to the total amount of species in solution.

3.3 Photostability study

Solutions of [Fe-BL], [Fe-BL-Co] and [Co] with $2.5 \cdot 10^{-3}$ mol/L concentration were irradiated by a 300 W Xe lamp in a NMR tube. Before irradiation, after 2.5 and 22 hours a ^{1}H NMR spectrum was recorded. Proton signals at δ = 8.22 ppm and δ = 7.52 ppm appear (*cf*. Figure S10, A and B) which can be assigned to a terminal pyridine ring due to its characteristic coupling pattern and are comparable to H_A and H_B in [Fe-BL-Co]. Furthermore, an additional singlet proton signal is detected at δ = 7.09 ppm similar to H_C which has the only singlet proton signal in the aromatic region of the iron part. Most likely, the crucial pyridine-cobalt bond is destabilized or a twist in the 4,4'-bipyridine moiety occurs by irradiation causing shifts of characteristic proton signals H_A-H_C to A-C.

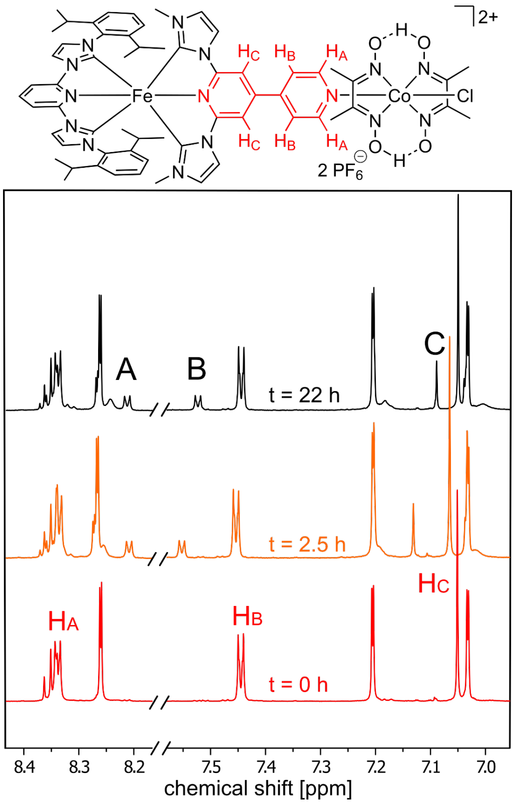

Figure S10: Selected aromatic regions of ^{1}H NMR spectra of [Fe-BL-Co] before irradiation with a 300 W xenon lamp (red), after 2.5 h (orange) and after 22 h irradiation (black) in acetonitrile-d_3 at 2.5mM (700 MHz, 298 K). A-C are assigned to new proton peaks which are similar to H_A-H_C.

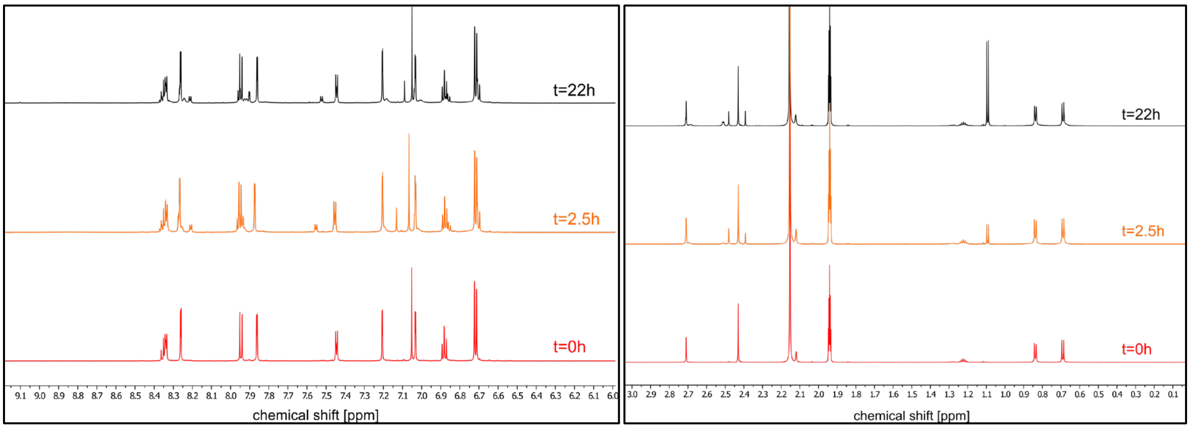

Figure S11: Aromatic (left) and aliphatic (right) region of ^{1}H NMR spectra of [Fe-BL-Co] before irradiation with a 300 W xenon lamp (red), after 2.5 h (orange) and after 22 h irradiation (black) in acetonitrile-d_3 at 2.5mM (700 MHz, 298 K).

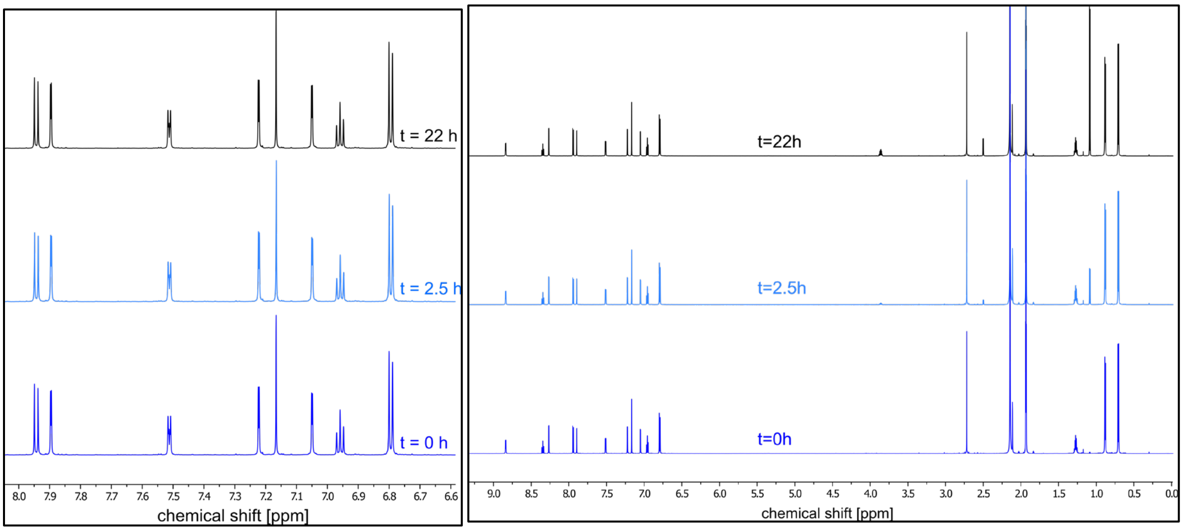

Figure S12: Left) Aromatic region of ^{1}H NMR spectra of [Fe-BL] before irradiation with a 300 W xenon lamp (blue), after 2.5 h (light blue) and after 22 h irradiation (black). Right) Full ^{1}H-NMR spectrum of [Fe-BL] in acetonitrile-d_3 at 2.5mM (700 MHz, 298 K).

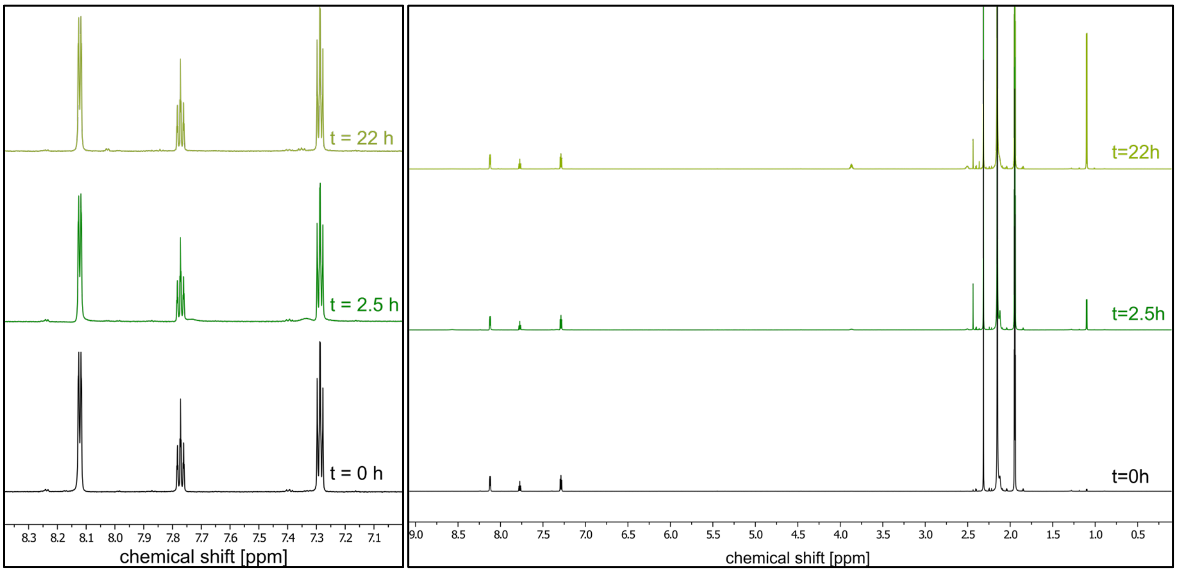

Figure S13: Left) Aromatic region of ^{1}H NMR spectra of [Co] before irradiation with a 300 W xenon lamp (black), after 2.5 h (green) and after 22 h irradiation (light green). Right) Full ^{1}H NMR spectra of [Co] in acetonitrile-d_3 at 2.5mM (700 MHz, 298 K).

4. UV-Vis spectroscopy

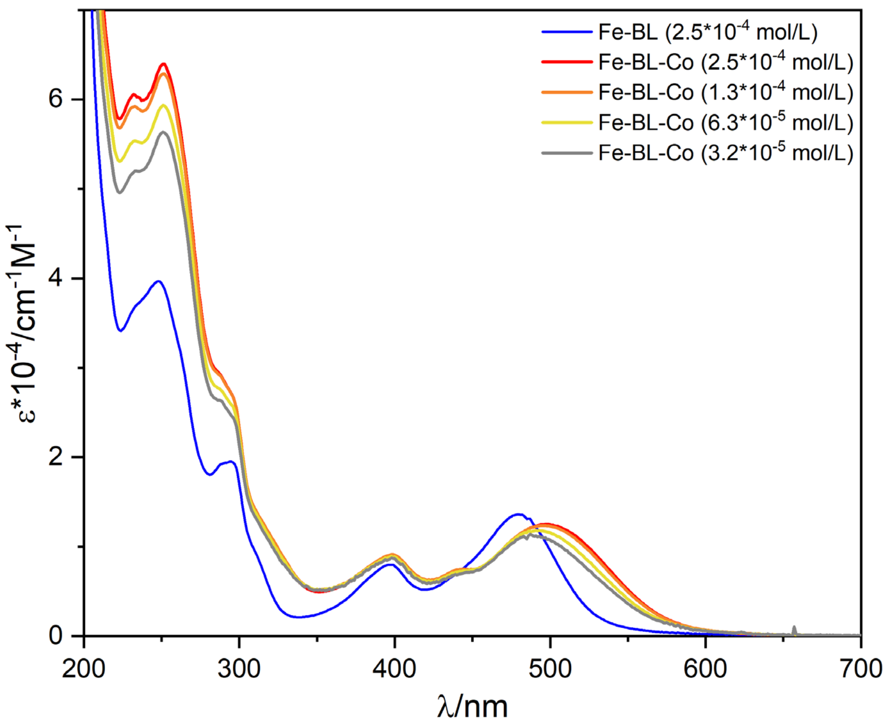

Figure S14: Concentration-dependent UV-Vis spectra of [Fe-BL] ($2.5\cdot10^{-3}$ mol/L) and [Fe-BL-Co] ($2.5\cdot10^{-3}$, $1.3\cdot10^{-3}$, $6.3\cdot10^{-4}$, $3.2\cdot10^{-4}$ mol/L) in acetonitrile. Band maximum lowest in energy varies from 486 to 498 nm for [Fe-BL-Co].

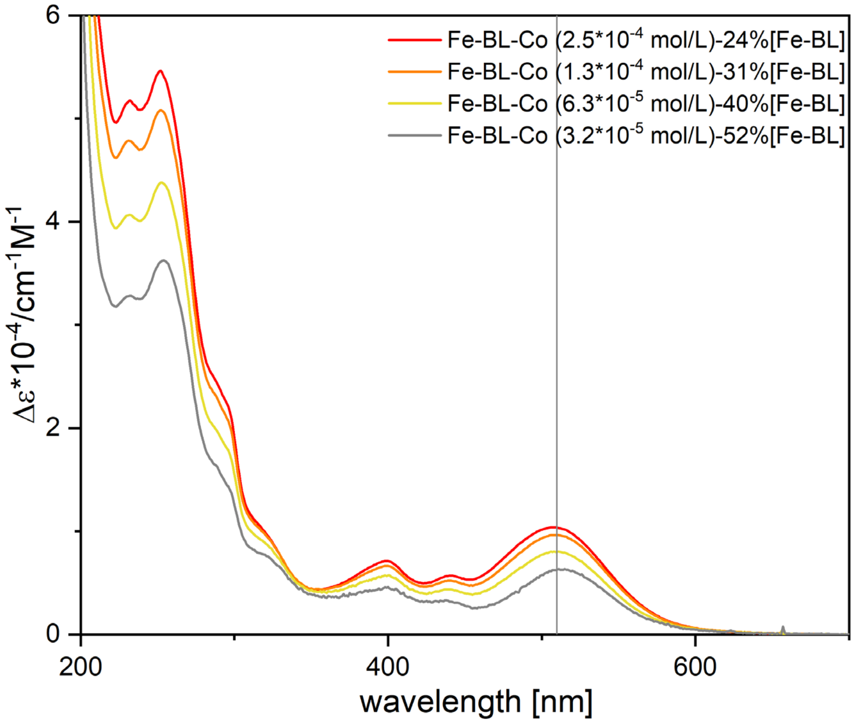

Figure S15: Photosensitizer corrected UV-Vis spectra of [Fe-BL-Co] ($2.5\cdot10^{-3}$, $1.3\cdot10^{-3}$, $6.3\cdot10^{-4}$, $3.2\cdot10^{-4}$ mol/L) in acetonitrile. Photosensitizer fraction at a certain concentration was deduced from NMR dissociation study. Grey, vertical reference line at 510 nm helps to see that after photosensitizer subtraction the band maximum is around 510 nm for [Fe-BL-Co] instead of 486 nm for each concentration.

5. Electrochemistry

Analysis of [Fe-BL]

A glassy carbon electrode (2mm diameter) was used to measure cyclic and square-wave voltammograms.

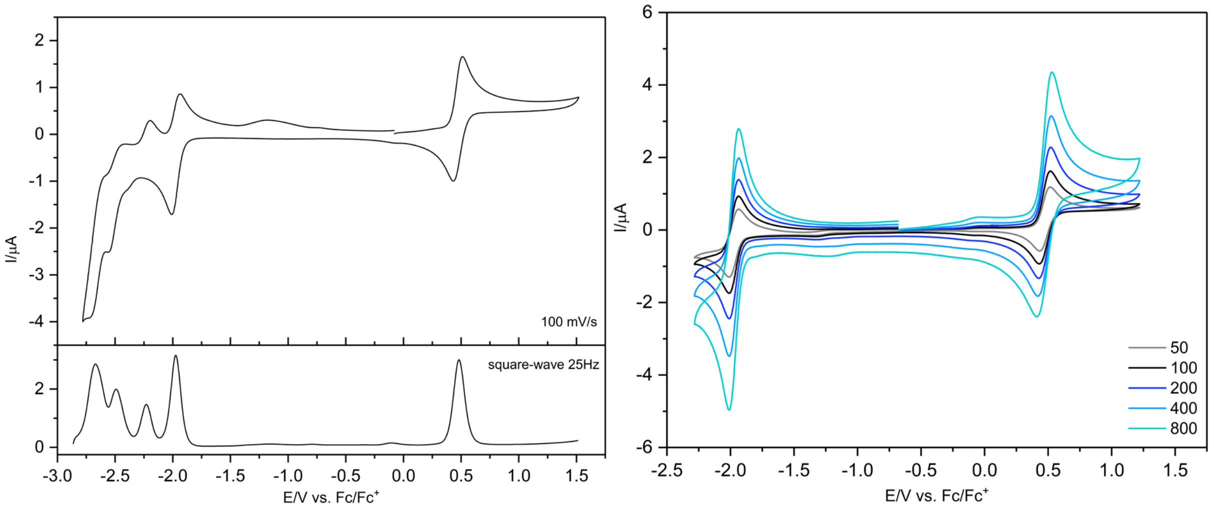

Figure S16: Left) Cyclic and square-wave voltammograms of [Fe-BL] vs. Fc/Fc^+ in MeCN with 100mV/s scan rate and 25 Hz frequency, respectively. Right) Cyclic voltammograms of [Fe-BL] with scan rate ranges from 50-800mV/s.

Table S6: Electrochemical data for reversible oxidation at $E^0_{1/2}$ = 0.47 V vs. Fc/Fc^+ in MeCN.

v [mV/s]	50	100	200	400	800
E_{pa} [V]	0.51	0.52	0.52	0.52	0.53
E_{pc} [V]	0.44	0.43	0.43	0.42	0.41
$E_{1/2}$ [V]	0.475	0.473	0.471	0.471	0.470
ΔE [mV]	76	85	93	98	115
I_{pa} [10^{-6} A]	0.99	1.39	1.92	2.63	3.56
I_{pc} [10^{-6} A]	-0.91	-1.34	-1.82	-2.14	-3.05
I_{pa}/I_{pc}	1.09	1.03	1.06	1.23	1.17
$I_{pa}/\sqrt{v}$	0.14	0.14	0.14	0.13	0.13
$I_{pc}/\sqrt{v}$	-0.13	-0.13	-0.13	-0.11	-0.11

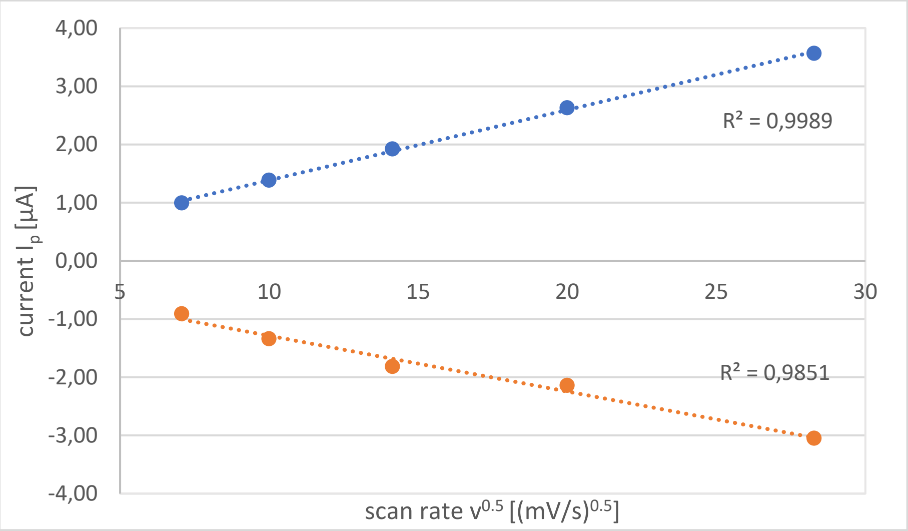

Figure S17: Linear dependence of peak current I_{pa}(blue) and I_{pc} (orange) versus square-root of scan rate v for reversible redox processes at $E^0_{1/2}$ = 0.47 V (linear fits in corresponding color).

Table S7: Electrochemical data for reversible reduction at $E^0_{1/2}$ = -1.97 V vs. Fc/Fc^+ in MeCN.

v [mV/s]	50	100	200	400	800
E_{pc} [V]	-2.01	-2.01	-2.01	-2.01	-2.01
E_{pa} [V]	-1.94	-1.94	-1.94	-1.94	-1.94
$E_{1/2}$ [V]	-1.97	-1.97	-1.97	-1.97	-1.97
ΔE [mV]	66	68	66	68	68
I_{pc} [10^{-6} A]	-0.89	-1.26	-1.78	-2.51	-3.52
I_{pa} [10^{-6} A]	1.03	1.49	2.17	3.01	4.31
I_{pa}/I_{pc}	1.15	1.18	1.22	1.20	1.22
$I_{pc}/\sqrt{v}$	-0.13	-0.13	-0.13	-0.13	-0.12
$I_{pa}/\sqrt{v}$	0.15	0.15	0.15	0.15	0.15

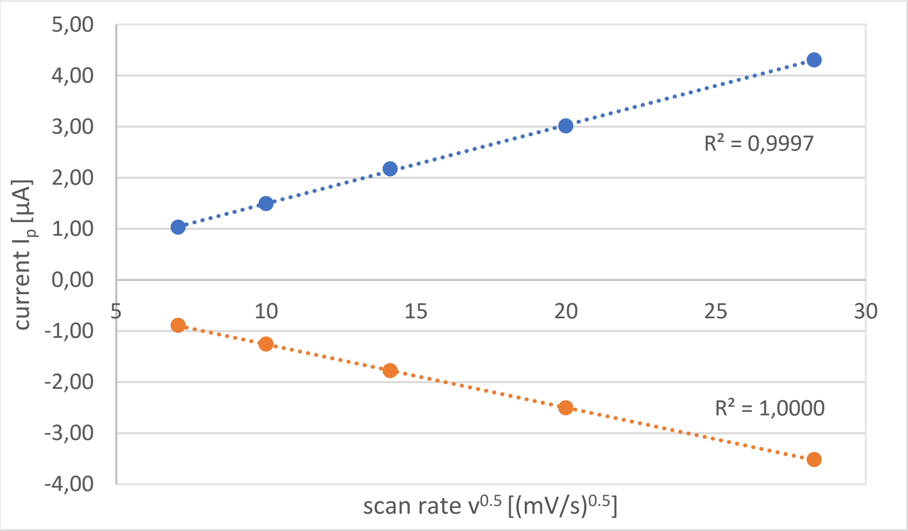

Figure S18: Linear dependence of peak current I_{pa}(blue) and I_{pc} (orange) versus square-root of scan rate v for reversible redox processes at $E^0_{1/2}$ = -1.97 V (linear fits in corresponding color).

Analysis of [Fe-BL-Co]

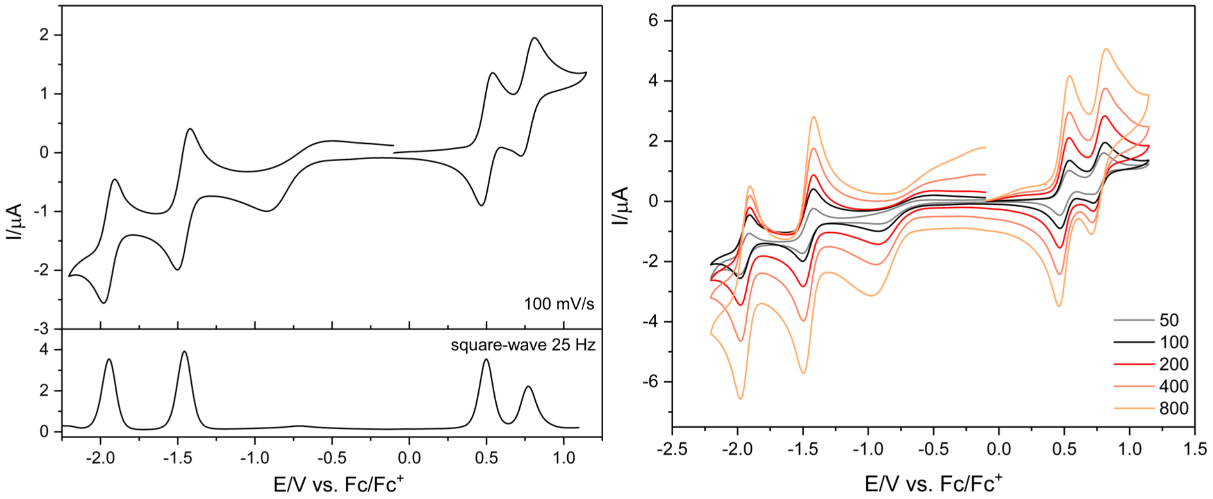

Figure S19: Left) Cyclic and square-wave voltammograms of [Fe-BL-Co] vs. Fc/Fc^+ in MeCN with 100mV/s scan rate and 25 Hz frequency, respectively. Right) Cyclic voltammograms of [Fe-BL-Co] with scan rate ranges from 50-800mV/s.

Table S8: Electrochemical data for reversible oxidation at $E^0_{1/2}$ = 0.77 V vs. Fc/Fc^+ in MeCN.

v [mV/s]	50	100	200	400	800
E_{pa} [V]	0.80	0.81	0.81	0.81	0.80
E_{pc} [V]	0.74	0.74	0.73	0.73	0.72
$E_{1/2}$ [V]	0.768	0.771	0.769	0.769	0.761
ΔE [mV]	59	71	76	85	79
I_{pa} [10^{-6} A]	0.68	0.86	1.27	1.60	1.99
I_{pc} [10^{-6} A]	-0.45	-0.59	-0.87	-1.15	-1.48
I_{pa}/I_{pc}	1.52	1.44	1.45	1.39	1.34
$I_{pa}/\sqrt{v}$	0.10	0.09	0.09	0.08	0.07
$I_{pc}/\sqrt{v}$	-0.06	-0.06	-0.06	-0.06	-0.05

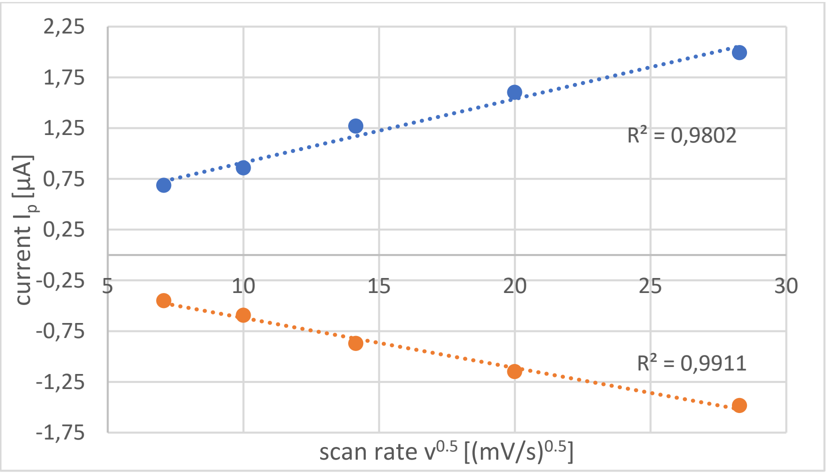

Figure S20: Linear dependence of peak current I_{pa}(blue) and I_{pc} (orange) versus square-root of scan rate v for reversible redox processes at $E^0_{1/2}$ = 0.77 V (linear fits in corresponding color).

Table S9: Electrochemical data for reversible oxidation at $E^0_{1/2}$ = 0.50 V vs. Fc/Fc^+ in MeCN.

v [mV/s]	50	100	200	400	800
E_{pa} [V]	0.53	0.53	0.53	0.53	0.53
E_{pc} [V]	0.46	0.46	0.46	0.46	0.46
$E_{1/2}$ [V]	0.497	0.498	0.494	0.495	0.493
ΔE [mV]	63	66	63	66	71
I_{pa} [10^{-6} A]	0.58	0.82	1.25	1.76	2.43
I_{pc} [10^{-6} A]	-0.71	-0.96	-1.42	-2.05	-2.86
I_{pa}/I_{pc}	0.81	0.85	0.88	0.86	0.85
$I_{pa}/\sqrt{v}$	0.08	0.08	0.09	0.09	0.09
$I_{pc}/\sqrt{v}$	-0.10	-0.10	-0.10	-0.10	-0.10

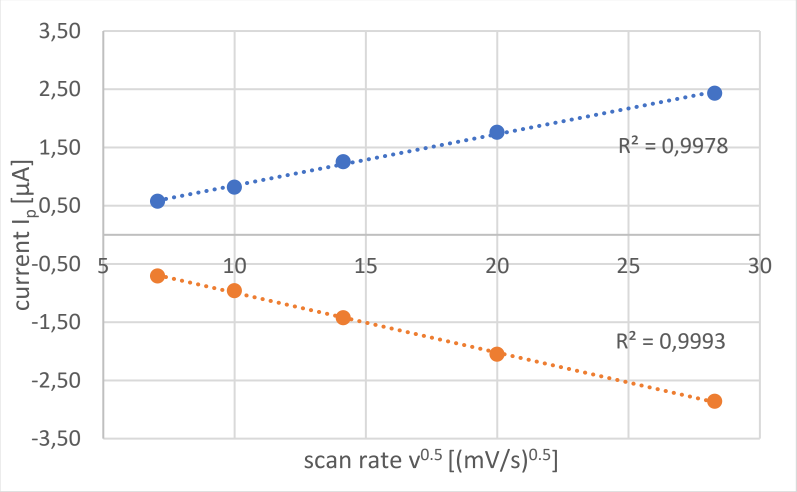

Figure S21: Linear dependence of peak current I_{pa}(blue) and I_{pc} (orange) versus square-root of scan rate v for reversible redox processes at $E^0_{1/2}$ = 0.50 V (linear fits in corresponding color).

Table S10: Electrochemical data for quasi-reversible reduction at $E^0_{1/2}$ = -1.46 V vs. Fc/Fc^+ in MeCN.

v [mV/s]	50	100	200	400	800
E_{pc} [V]	-1.50	-1.50	-1.50	-1.49	-1.49
E_{pa} [V]	-1.43	-1.42	-1.42	-1.42	-1.42
$E_{1/2}$ [V]	-1.46	-1.46	-1.46	-1.46	-1.46
ΔE [mV]	71	76	71	71	73
I_{pc} [10^{-6} A]	-0.72	-1.02	-1.53	-2.17	-3.00
I_{pa} [10^{-6} A]	0.82	1.24	1.76	2.57	3.72
I_{pa}/I_{pc}	1.13	1.22	1.16	1.18	1.24
$I_{pc}/\sqrt{v}$	-0.10	-0.10	-0.11	-0.11	-0.11
$I_{pa}/\sqrt{v}$	0.12	0.12	0.12	0.13	0.13

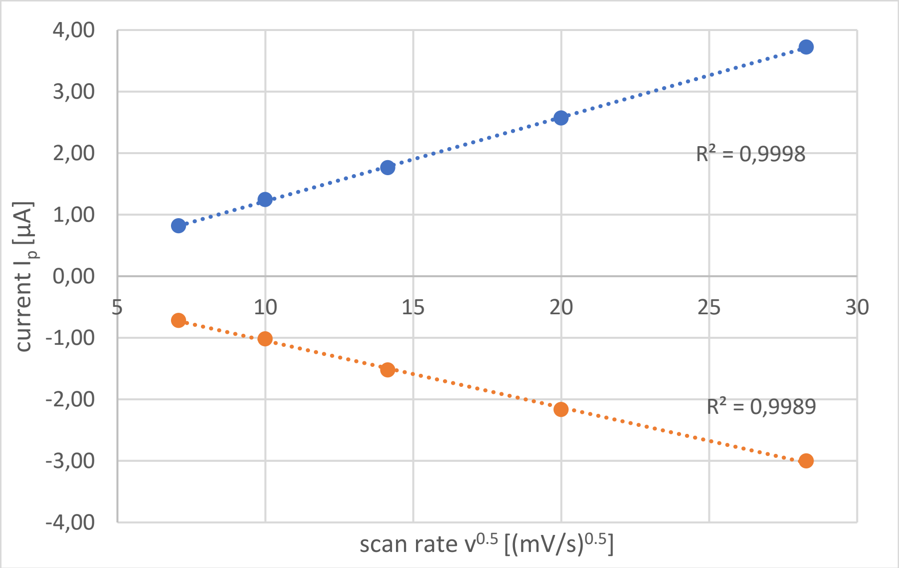

Figure S22: Linear dependence of peak current I_{pa}(blue) and I_{pc} (orange) versus square-root of scan rate v for quasi-reversible redox processes at $E^0_{1/2}$ = -1.46 V (linear fits in corresponding color).

Table S11: Electrochemical data for quasi-reversible reduction at $E^0_{1/2}$ = -1.94 V vs. Fc/Fc^+ in MeCN.

v [mV/s]	50	100	200	400	800
E_{pc} [V]	-1.97	-1.98	-1.97	-1.97	-1.97
E_{pa} [V]	-1.92	-1.92	-1.91	-1.91	-1.92
$E_{1/2}$ [V]	-1.94	-1.95	-1.94	-1.94	-1.94
ΔE [mV]	59	61	66	61	59
I_{pc} [10^{-6} A]	-0.56	-0.87	-1.31	-1.94	-2.77
I_{pa} [10^{-6} A]	0.68	1.05	1.59	2.25	3.21
I_{pa}/I_{pc}	1.21	1.21	1.22	1.16	1.16
$I_{pc}/\sqrt{v}$	-0.08	-0.09	-0.09	-0.10	-0.10
$I_{pa}/\sqrt{v}$	0.10	0.11	0.11	0.11	0.11

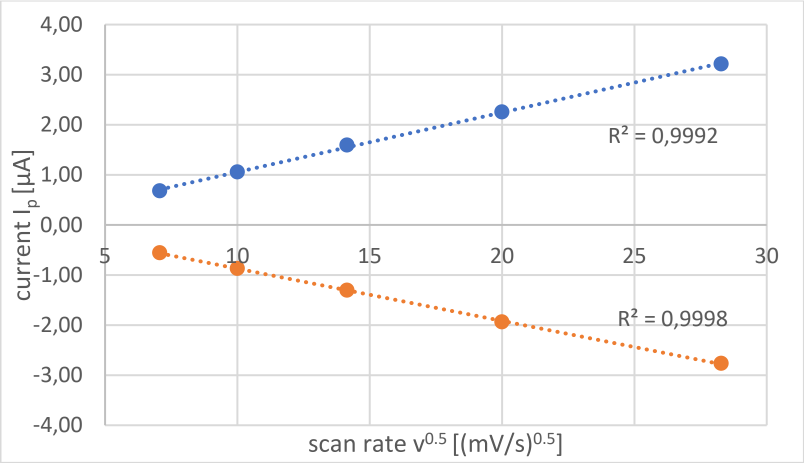

Figure S23: Linear dependence of peak current I_{pa}(blue) and I_{pc} (orange) versus square-root of scan rate v for reversible redox processes at $E^0_{1/2}$ = -1.94 V (linear fits in corresponding color).

6. Quantum chemical calculations

Table S12: XYZ files of all optimized structures.

Optimized gas phase structure of [Fe-BL] in cartesian coordinates (Angstroem):

122

Fe	1.919978	10.163521	18.555682
N	4.790617	11.065640	17.519545
N	3.977320	11.912957	19.294874
N	1.924088	11.417878	20.023131
N	-0.129644	10.626571	20.407147
N	-0.949536	9.005150	19.298028
C	2.746974	8.596513	19.414405
N	1.915593	8.911636	17.089198
C	1.093127	11.260593	17.146312
N	1.933631	4.385667	11.762265
C	3.694697	11.018918	18.291695
C	5.738986	11.940562	18.034556
H	6.691101	12.097131	17.559591
C	5.230827	12.480729	19.151229
H	5.650387	13.198735	19.833602
C	4.955836	10.403565	16.254020
C	4.602366	11.105161	15.092387
C	4.775373	10.451794	13.876170
H	4.522794	10.957210	12.954439
C	5.288784	9.169991	13.819306
H	5.424597	8.683598	12.862233
C	5.665922	8.519159	14.979075
H	6.095359	7.530358	14.905110
C	5.525498	9.124555	16.223720
C	6.068433	8.439542	17.463148
H	5.417602	8.675250	18.310728
C	7.474441	8.956476	17.790773
H	7.493398	10.019373	18.021380
H	8.152176	8.786476	16.953705
H	7.880277	8.432808	18.656992
C	6.134848	6.919636	17.326233
H	5.209570	6.492304	16.939104
H	6.340865	6.467557	18.295500
H	6.940192	6.609607	16.659485
C	4.134675	12.548757	15.104690
H	3.562074	12.725039	16.021079
C	3.235468	12.900123	13.919342
H	2.458140	12.159462	13.746984
H	3.807075	13.005771	12.996650
H	2.747760	13.858811	14.094529
C	5.331173	13.508379	15.087136
H	5.980323	13.405603	15.952838
H	4.987396	14.543013	15.063843

H	5.940220	13.342815	14.197888
C	2.994905	12.168265	20.238959
C	3.052958	13.080997	21.277249
H	3.923690	13.694599	21.455872
C	1.930108	13.184150	22.088212
C	0.804256	12.402066	21.864575
C	0.856250	11.517623	20.801853
C	0.147476	9.774019	19.367105
C	-1.380650	10.397078	20.951795
H	-1.795847	10.959729	21.769236
C	-1.892917	9.378291	20.246999
H	-2.844735	8.885225	20.333353
C	-1.113465	7.855021	18.450907
C	-0.766675	6.600087	18.971348
C	-0.926191	5.498165	18.136875
H	-0.677424	4.510204	18.498859
C	-1.421530	5.638227	16.854388
H	-1.546391	4.767071	16.224676
C	-1.796836	6.883382	16.385027
H	-2.214087	6.961101	15.391293
C	-1.669841	8.020357	17.176417
C	-2.214906	9.348386	16.687530
H	-1.566131	10.151301	17.050558
C	-3.621913	9.589231	17.247630
C	-2.280797	9.445715	15.164616
C	-0.323852	6.391672	20.407484
H	0.225773	7.278481	20.739381
C	0.595641	5.183396	20.584187
H	1.391551	5.152118	19.844270
H	0.045043	4.244023	20.524006
H	1.058856	5.209496	21.570348
C	-1.538272	6.198776	21.324201
H	-2.213227	7.050815	21.332388
H	-1.215802	6.032142	22.352486
H	-2.115166	5.327107	21.013463
N	3.318717	8.163596	20.541453
C	3.779197	6.862869	20.413147
C	3.476829	6.454751	19.170121
N	2.840331	7.523108	18.571845
C	2.372457	7.683638	17.271934
C	2.378806	6.744968	16.262967
C	1.920426	7.140289	15.005774
C	1.458987	8.443493	14.816733
H	1.087771	8.758389	13.852022
C	1.460845	9.288477	15.905393
N	0.994142	10.597878	15.953913
C	0.362332	11.361369	14.993478
C	0.069073	12.526977	15.592142
N	0.529307	12.445534	16.896778

C	2.341200	5.633898	11.598469
C	2.352898	6.573508	12.618959
C	1.924554	6.187609	13.884338
C	1.500881	4.874637	14.057976
H	1.131032	4.524992	15.013479
C	1.521601	4.020023	12.965401
H	1.186798	2.994406	13.068572
C	0.379091	13.504416	17.874210
C	3.486451	8.964839	21.736839
H	3.657121	5.512426	18.684545
H	4.268897	6.337441	21.215033
H	2.548554	9.441662	22.007190
H	4.253037	9.723363	21.590839
H	3.789839	8.325030	22.560801
H	-0.362127	13.237273	18.624696
H	0.049333	14.411553	17.375637
H	1.329847	13.711091	18.357902
H	0.176626	11.030917	13.987272
H	-0.415802	13.404492	15.200045
H	-0.063957	12.485735	22.501604
H	1.932481	13.886997	22.909863
H	2.753221	5.743483	16.419409
H	2.679825	5.903328	10.604801
H	2.719614	7.574612	12.431021
H	-4.028836	10.525760	16.864810
H	-3.642363	9.651015	18.333532
H	-4.297494	8.787502	16.948167
H	-1.353988	9.132455	14.683404
H	-2.490508	10.472134	14.866609
H	-3.083515	8.831296	14.755386

Optimized gas phase structure of [Fe-BL-Co] in cartesian coordinates (Angstroem):

154

Fe	1.917260	10.163939	18.554758
Co	2.027347	3.092616	10.242935
Cl	2.082287	1.669433	8.579891
O	1.629263	1.015706	12.070907
O	4.548675	4.127063	9.300465
O	2.423371	5.234406	8.491156
O	-0.489371	1.920359	11.022969
N	4.786294	11.065006	17.513424
N	3.975438	11.913810	19.289310
N	1.923476	11.418642	20.021714
N	-0.129027	10.625602	20.409795
N	-0.949568	9.003432	19.302067
C	2.743929	8.598176	19.415232
N	1.911537	8.912175	17.089547
C	1.090074	11.261686	17.146929
N	1.979512	4.397343	11.769337
N	2.514280	1.683299	11.433191

N	3.917474	3.265461	10.094636
N	1.541658	4.471403	9.017290
N	0.139899	2.858444	10.319041
C	3.691670	11.019031	18.287430
C	5.735146	11.940943	18.026122
H	6.686576	12.097109	17.549593
C	5.228568	12.481778	19.143192
H	5.648974	13.200576	19.824238
C	4.952073	10.400622	16.248996
C	4.602610	11.101089	15.085307
C	4.781407	10.447240	13.870017
H	4.534279	10.952661	12.946789
C	5.296839	9.166056	13.815659
H	5.441494	8.681485	12.858782
C	5.667873	8.515505	14.977724
H	6.100495	7.527859	14.906115
C	5.521637	9.121372	16.221557
C	6.059853	8.436996	17.463410
H	5.407789	8.675867	18.309161
C	7.466381	8.951085	17.793326
H	7.487157	10.014384	18.021858
H	8.145704	8.777982	16.958201
H	7.868915	8.428235	18.661556
C	6.122617	6.916658	17.329270
H	5.197066	6.490875	16.940822
H	6.325286	6.465605	18.299698
H	6.928735	6.603506	16.664936
C	4.135179	12.544805	15.093893
H	3.561507	12.723253	16.009237
C	3.237821	12.893495	13.906254
H	2.461303	12.152044	13.733345
H	3.810876	12.998065	12.984381
H	2.749189	13.852154	14.078866
C	5.331871	13.504263	15.075683
H	5.979669	13.403806	15.942642
H	4.988103	14.538780	15.049076
H	5.942245	13.336400	14.187797
C	2.994586	12.169228	20.235359
C	3.054372	13.081819	21.273581
H	3.925197	13.695708	21.450831
C	1.933094	13.184285	22.086894
C	0.807006	12.401773	21.865554
C	0.857152	11.517575	20.802636
C	0.146360	9.773852	19.368975
C	-1.377941	10.393507	20.958198
H	-1.791707	10.955102	21.777113
C	-1.890884	9.374599	20.253992
H	-2.841822	8.880137	20.342382
C	-1.116403	7.854851	18.453222
C	-0.768744	6.598475	18.969886
C	-0.935973	5.497671	18.135274
H	-0.688114	4.508612	18.494911
C	-1.440078	5.639759	16.856349

H	-1.574377	4.768610	16.228334
C	-1.814624	6.886569	16.390413
H	-2.240198	6.966120	15.400313
C	-1.679507	8.022647	17.181928
C	-2.225595	9.352000	16.697728
H	-1.574426	10.153813	17.058919
C	-3.629589	9.593336	17.265140
C	-2.298606	9.451695	15.175240
C	-0.316953	6.386955	20.402796
H	0.235388	7.272843	20.732781
C	0.602566	5.177326	20.570847
H	1.392805	5.145677	19.824806
H	0.050465	4.238690	20.513645
H	1.073322	5.201499	21.553451
C	-1.525459	6.193076	21.327120
H	-2.199220	7.045958	21.342642
H	-1.196004	6.023087	22.352607
H	-2.105446	5.322935	21.017951
N	3.315386	8.165337	20.541998
C	3.773810	6.863579	20.414693
C	3.470670	6.454360	19.172422
N	2.835146	7.523401	18.573275
C	2.367961	7.684282	17.274466
C	2.374213	6.745119	16.265095
C	1.920935	7.145078	15.008741
C	1.460900	8.446954	14.815510
H	1.096371	8.763521	13.848756
C	1.458408	9.291133	15.905680
N	0.991825	10.599309	15.953641
C	0.361423	11.364108	14.992676
C	0.068036	12.528984	15.592246
N	0.526725	12.446441	16.897587
C	2.409600	5.648171	11.604971
C	2.400480	6.572119	12.633121
C	1.935413	6.194112	13.885530
C	1.489462	4.888827	14.043538
H	1.090240	4.538973	14.986154
C	1.524393	4.024403	12.965328
H	1.186322	3.001816	13.075394
C	4.375126	0.381748	12.365209
H	5.071550	0.765276	13.113183
H	4.924963	-0.324334	11.740450
H	3.590802	-0.168716	12.877015
C	3.788093	1.475006	11.542273
C	4.596788	2.386579	10.734724
C	6.075905	2.273634	10.608434
H	6.332664	1.897275	9.616316
H	6.492088	1.591541	11.344495
H	6.561466	3.242694	10.715159
C	-0.309317	5.534746	7.805782
H	-1.055611	6.181169	8.271093
H	-0.799734	5.010829	6.983385
H	0.470850	6.160546	7.381735

C	0.272296	4.570138	8.779494
C	-0.534282	3.603560	9.522847
C	-2.002686	3.444599	9.335741
H	-2.211257	2.488960	8.851300
H	-2.418110	4.234493	8.716510
H	-2.527937	3.436794	10.290235
H	3.792562	4.623516	8.828112
H	0.266680	1.399907	11.467750
C	0.375317	13.505168	17.875391
C	3.484812	8.966900	21.737263
H	3.649382	5.510956	18.688345
H	4.262844	6.338157	21.217028
H	2.547336	9.443950	22.008558
H	4.251646	9.724907	21.590235
H	3.788831	8.326973	22.560872
H	-0.365338	13.236669	18.625898
H	0.044057	14.411794	17.376958
H	1.325914	13.713054	18.358818
H	0.176334	11.035019	13.985899
H	-0.416072	13.406989	15.200223
H	-0.059738	12.484967	22.504658
H	1.936781	13.887211	22.908486
H	2.744949	5.742336	16.422149
H	2.765095	5.929806	10.621882
H	2.784068	7.567015	12.450752
H	-4.037420	10.530949	16.885997
H	-3.644382	9.653270	18.351237
H	-4.307519	8.792816	16.967805
H	-1.374493	9.137882	14.688982
H	-2.508212	10.478804	14.879634
H	-3.104233	8.839090	14.769040

Table S13: Löwdin %Fe 3d character of dπ, dπ* and dσ* orbitals of investigated complexes.

orbital	[Fe-BL] /%	[Fe-BL-Co] /%
HOMO-2/4	73.90	73.10
HOMO-1/3	72.40	72.40
HOMO	58.40	58.50
average dπ	68.23	68.00
LUMO	5.60	4.90
LUMO+1/2	7.30	7.00
LUMO+2/4	7.10	5.60
average dπ*	6.67	5.83
LUMO+10/14	62.30	63.10
LUMO+14/18	54.60	55.10
average dσ*	58.45	59.10
HOMO-LUMO gap [eV] (nm)	2.77 (447)	2.56 (484)
dπ-dσ* gap (ΔO) [eV] (nm)	4.62 (269)	4.62 (269)

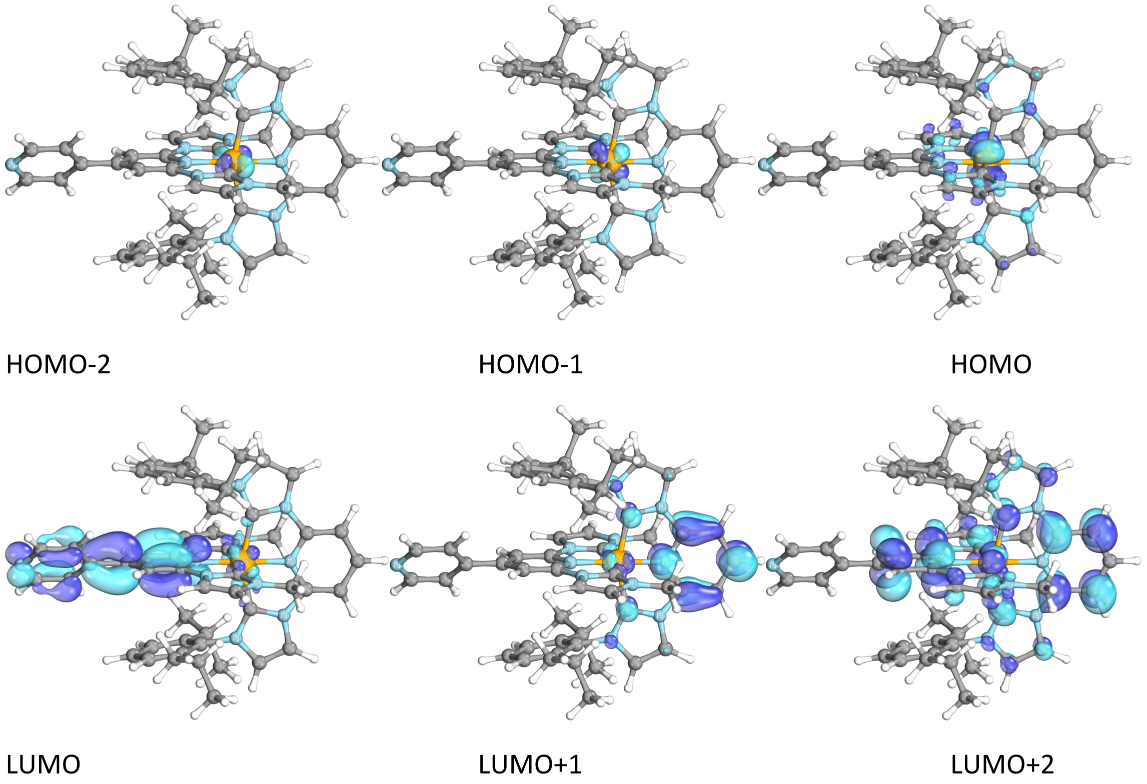

Figure S24: Frontier orbitals of [Fe-BL]; TPSSh, D3BJ, def2-TZVPP, SMD(acetonitrile). The dπ (HOMOs) and dπ* (LUMOs) orbitals are depicted.

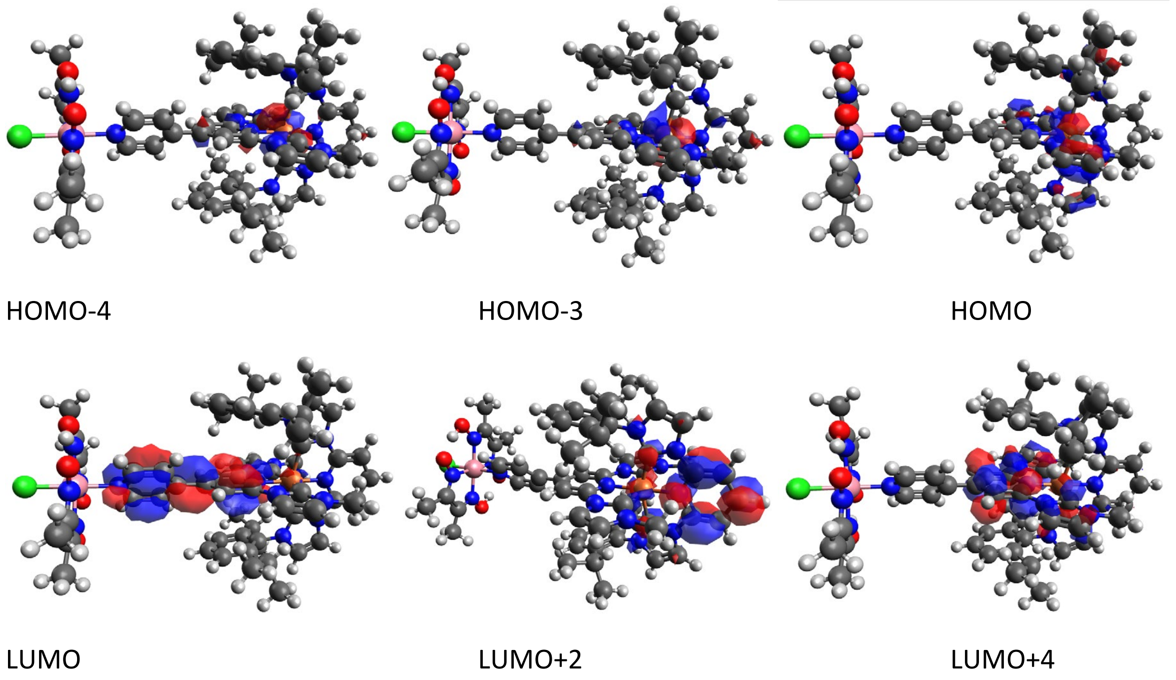

Figure S25: Frontier orbitals of [Fe-BL-Co]; TPSSh, D3BJ, def2-TZVPP, SMD(acetonitrile). The dπ (HOMOs) and dπ* (LUMOs) orbitals are depicted.

7. Optical transient absorption spectroscopy

Difference spectra of oxidized species ΔA_{ox} are used for comparison with DAS of transient optical absorption spectroscopy. Due to lower concentrations in the spectroelectrochemical measurements (c = 0.1 mM) dyad dissociation was taken into (*cf.* NMR dissociation study). This led to modified difference spectrum of oxidized [Fe-BL-Co] suitable for TA discussion (Figure S26, red curve).

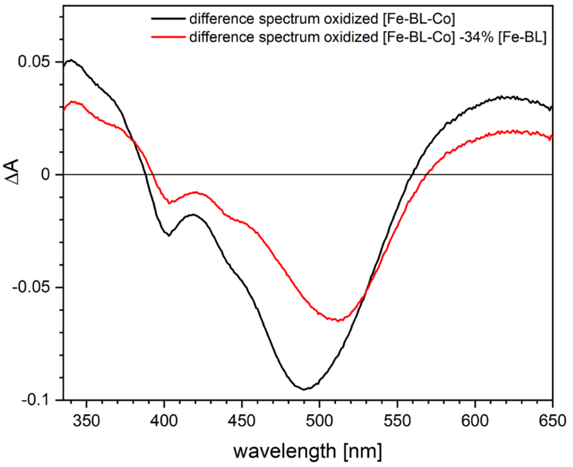

Figure S26: Difference spectrum of oxidized [Fe-BL-Co] and modified difference spectrum of oxidized [Fe-BL-Co] with 34% [Fe-BL] subtraction due to dyad dissociation in spectroelectrochemical experiment at 0.1 mM.

Standard fitting procedure with the convolution of the instrument response function (IRF) was applied using the multi-exponential function given below:

$$\Delta A = \left(A_1\, exp\left(-\frac{t}{\tau_1}\right) + A_2\, exp\left(-\frac{t}{\tau_2}\right) + A_3\, exp\left(-\frac{t}{\tau_3}\right) + offset\right) \otimes IRF.$$

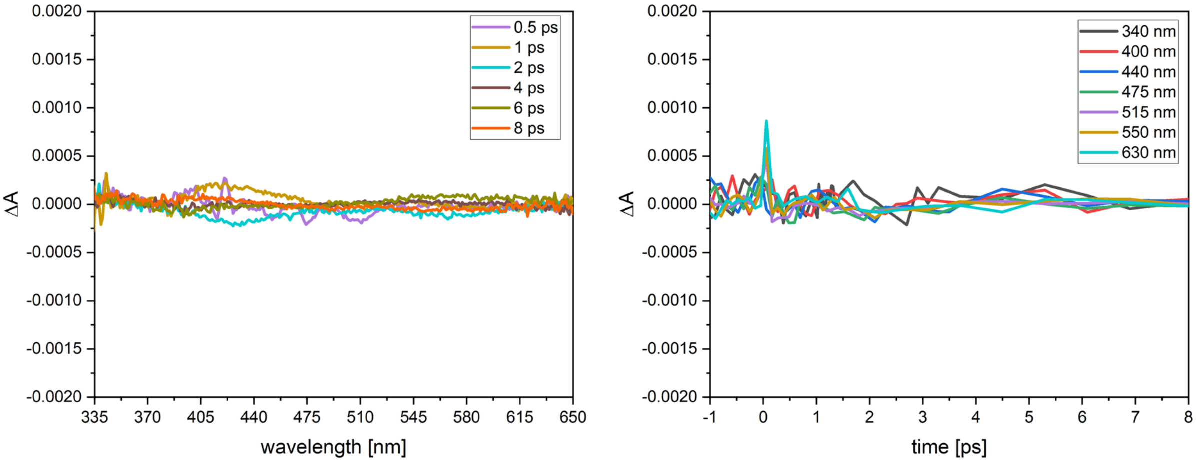

Figure S27: Optical transient absorption of 5mM [Co] in acetonitrile (λ_{exc} = 515 nm, E = 2 µJ). TA spectra (left) and TA kinetics (right).

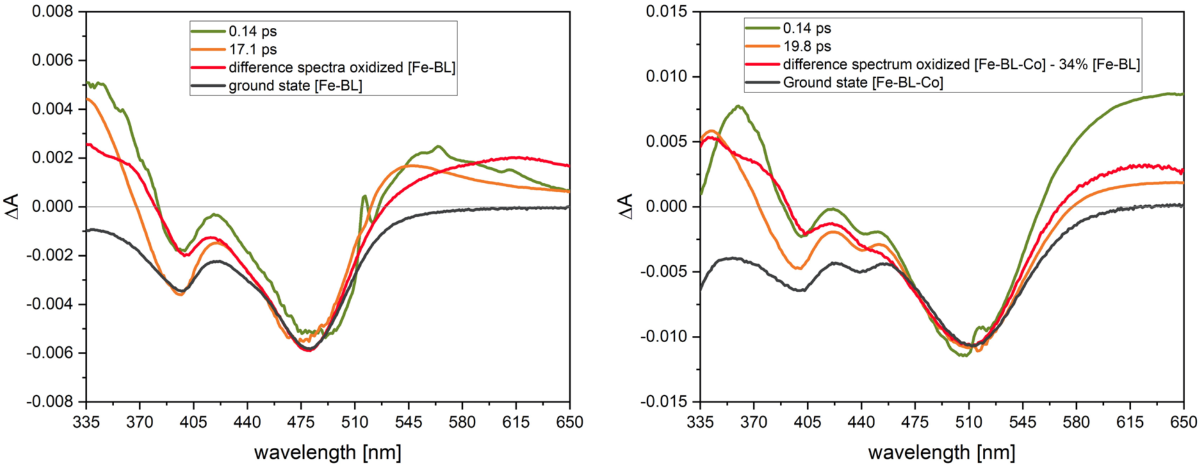

Figure S28: DAS of [Fe-BL] (left) and [Fe-BL-Co] (right) plotted together with ground state spectra and with differential spectroelectrochemical spectra (see difference spectrum of oxidized [Fe-BL-Co]-34% [Fe-BL] presented on Figure S26).

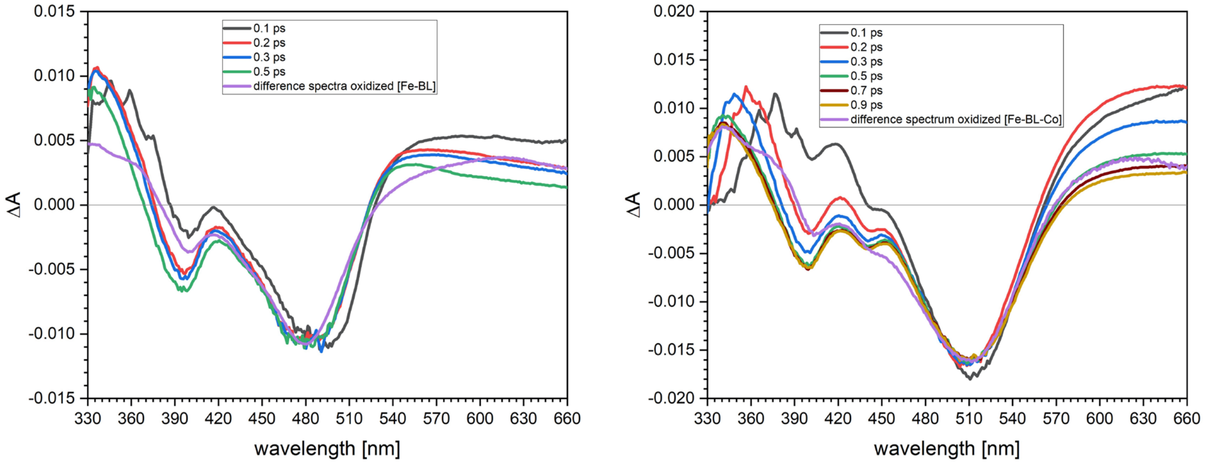

Figure S29: Fast spectral evolution of [Fe-BL] (left) and [Fe-BL-Co] (right) plotted together with differential spectroelectrochemical spectra (see difference spectrum of oxidized [Fe-BL-Co]-34% [Fe-BL] presented on Figure S26).

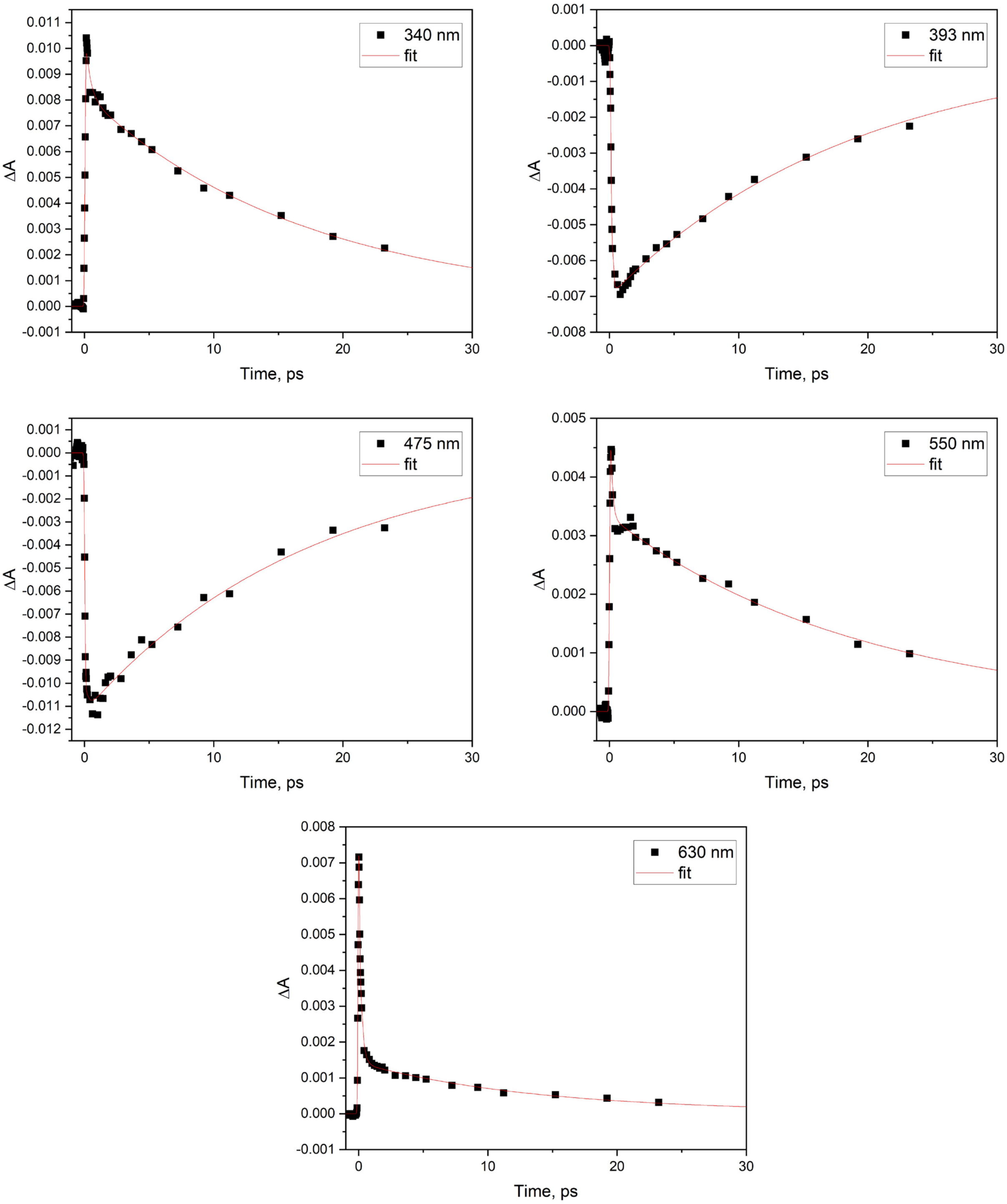

Figure S30: [Fe-BL] selected individual kinetics and their fits (only 1-30 ps region is shown).

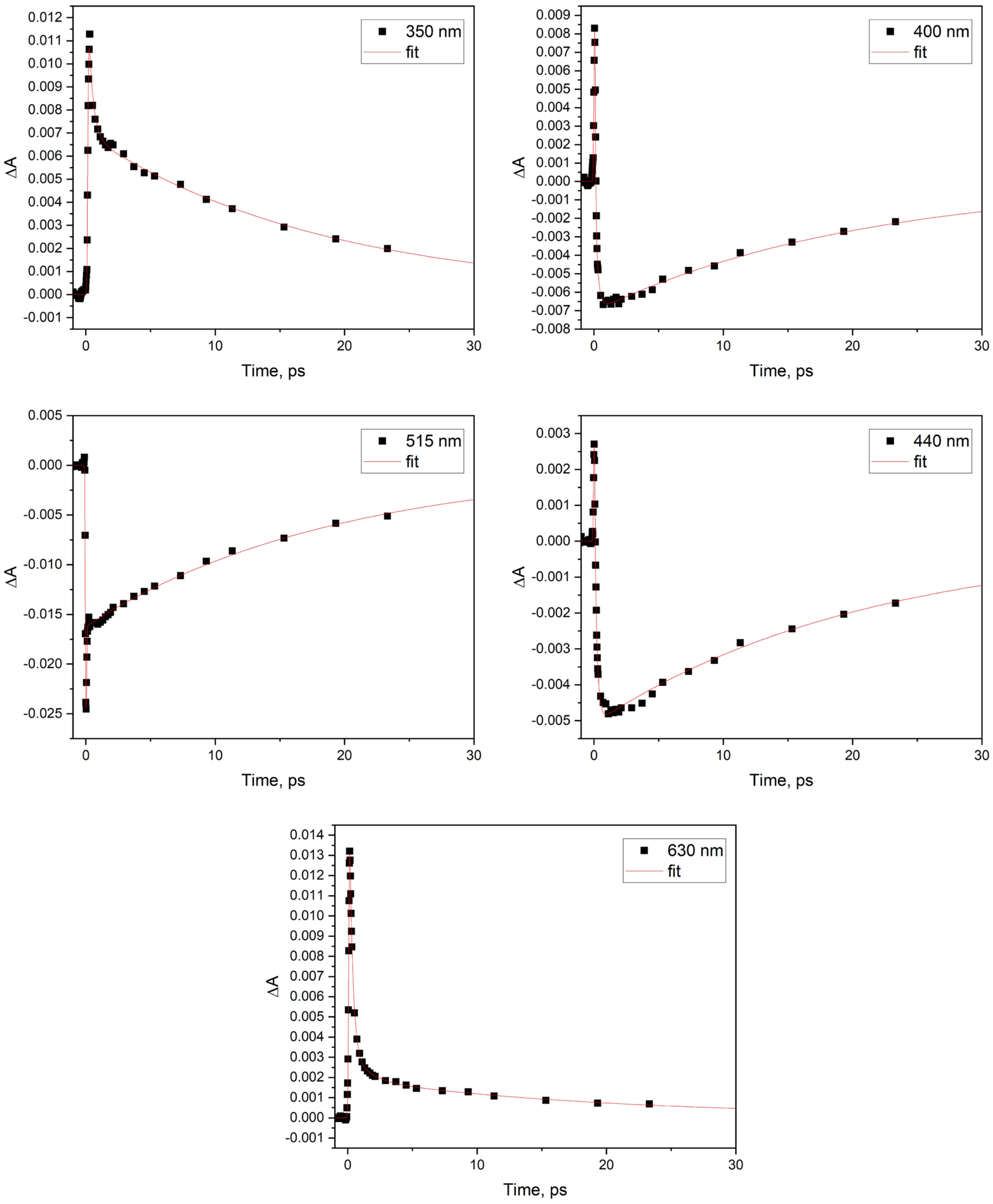

Figure S31: [Fe-BL-Co] selected individual kinetics and their fits (only 1-30 ps region is shown).

Table S14: Fit parameters of [Fe-BL] at selected individual kinetics used as initial guess for DAS construction. Time constants τ are given in picoseconds.

	340 nm	393 nm	475 nm	550 nm	630 nm
A_1	-	-	-	-	0.046 ± 0.005
τ_1	-	-	-	-	0.010 ± 0.001
A_2	0.002 ± 0.0003	0.007 ± 0.212	0.0010 ± 0.0007	0.0020 ± 0.0003	0.0050 ± 0.0003
τ_2	0.39 ± 0.12	0.09 ± 0.01	0.23 ± 0.21	0.15 ± 0.03	0.20 ± 0.01
A_3	0.0080 ± 0.0002	-0.007 ± 0.001	-0.011 ± 0.0002	0.00300 ± 0.00005	0.00100 ± 0.00004
τ_3	17.1 ± 0.8	19.0 ± 0.6	17.5 ± 0.9	19.2 ± 0.8	13.7 ± 1.0
offset	9.9E-5 ± 5.8E-5	-2.2E-5 ± 3.4E-5	9.9E-5 ± 8.8E-5	4.8E-6 ± 2.4E-5	4.2E-5 ± 1.4E-5

Table S15: Fit parameters of [Fe-BL-Co] at selected individual kinetics used as initial guess for DAS construction. Time constants τ are given in picoseconds.

	350 nm	400 nm	440 nm	515 nm	630 nm
A_1	-	0.024 ± 0.024	0.032 ± 0.002	-0.251 ± 0.062	0.0020 ± 0.0007*
τ_1	-	0.011 ± 0.011	0.050 ± 0.008	0.007 ± 0.002	1.33 ± 0.75*
A_2	0.0050 ± 0.0003	0.0080 ± 0.0006	0.006 ± 0.002	-	0.0150 ± 0.0007
τ_2	0.348 ± 0.043	0.168 ± 0.009	0.241 ± 0.067	-	0.238 ± 0.016
A_3	0.0070 ± 0.0002	-0.0050 ± 0.0001	-0.007 ± 0.001	-0.0160 ± 0.0002	0.0020 ± 0.0002
τ_3	18.2 ± 1.0	21.1 ± 0.7	20.5 ± 0.9	19.1 ± 0.7	19.8 ± 3.3
offset	3.3E-5 ± 5.5E-5	-6.9E-6 ± 2.6E-5	2.8E-6 ± 4.9E-5	-8.6E-5 ± 9.9E-5	4.8E-5 ± 3.5E-5

*This τ_1 component has a different nature than τ_1 component obtained on shorter wavelengths.

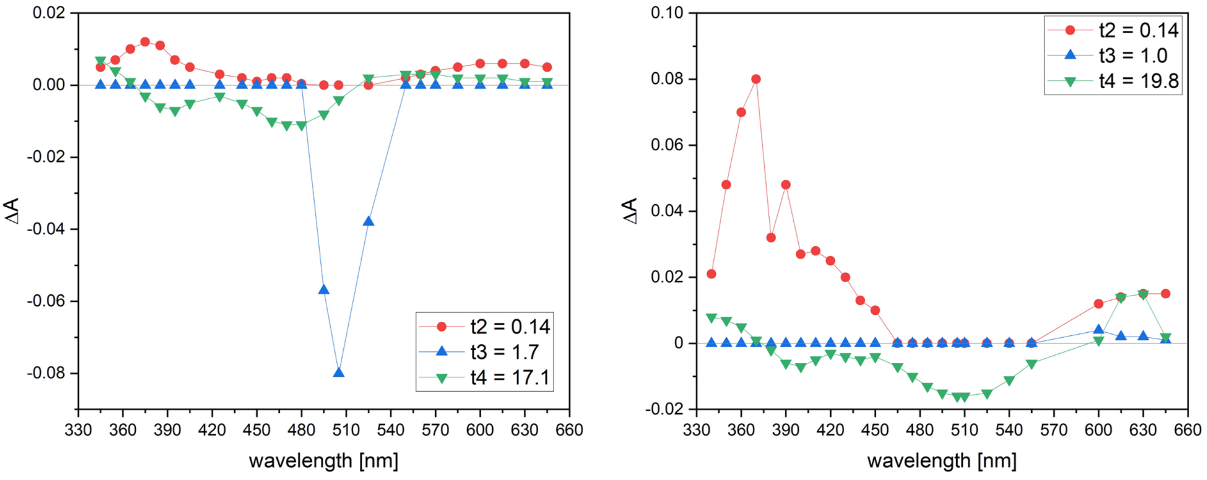

Figure S32: Fit amplitudes t2-t4 obtained from global analysis for [Fe-BL] (left) and [Fe-BL-Co] (right).

8. Photocatalytic proton reduction

The dyad [Fe-BL-Co] and the two-component systems [Fe-BL]+[Co] and [Ir(ppy)$_2$(bpy)]PF$_6$+[Co] as references were irradiated under isobaric conditions at 25 °C in a MeCN/water mixture (1:1) with 5 % TEOA as a sacrificial electron donor. In the two-component experiment the ratio photosensitizer: catalyst was chosen to 1:1 to match the ratio in the dyad.

Aqueous TEOA solution (10 %) was adjusted with concentrated HCl to pH = 7 and mixed 1:1 with MeCN. Solvent mixture was freeze-pumped to remove all gases. The reactor was prepared by evacuating and filling with argon for five times. All solid compounds were degassed in one sealed vial with argon. In this vial 20 ml of solvent mixture were added and the complete solution was transferred to the reactor. The solution was stirred for 5 minutes to allow the solution to equilibrate. While the light source was turned on the measurement was started simultaneously. Volume and temperature changes over time were recorded by a PC unit. [Fe-BL-Co] as well as [Fe-BL]+[Co] and [Ir(ppy)$_2$(bpy)]PF$_6$+[Co] were irradiated for three hours, using a AM 1.5 300 W Xenon lamp, covering the whole solar spectrum. All measurements were performed twice except for [Ir(ppy)$_2$(bpy)]PF$_6$+[Co]. Solvent mixture measurements were used as blind curves to exclude any solvent effects and subtracted from each measurement. Then any influence of temperature changes during the experiment was removed by a temperature correction approach.[13] Finally, the measurements were averaged.

Proof of hydrogen was conducted by headspace analysis via gas chromatography (micro-GC, *cf.* Figure S33 and S34). First broad peak belongs to a pressure burst caused by the GC apparatus. Second signal is assigned to hydrogen, third signal to oxygen and fourth to nitrogen. Oxygen and nitrogen originated from the experimental setup. Connection to the GC has to be switched between two proton reduction apparatus leading to O_2 and N_2 from the air. Produced hydrogen was quantified by the automatic gas burette connected to the proton reduction apparatus.[13]

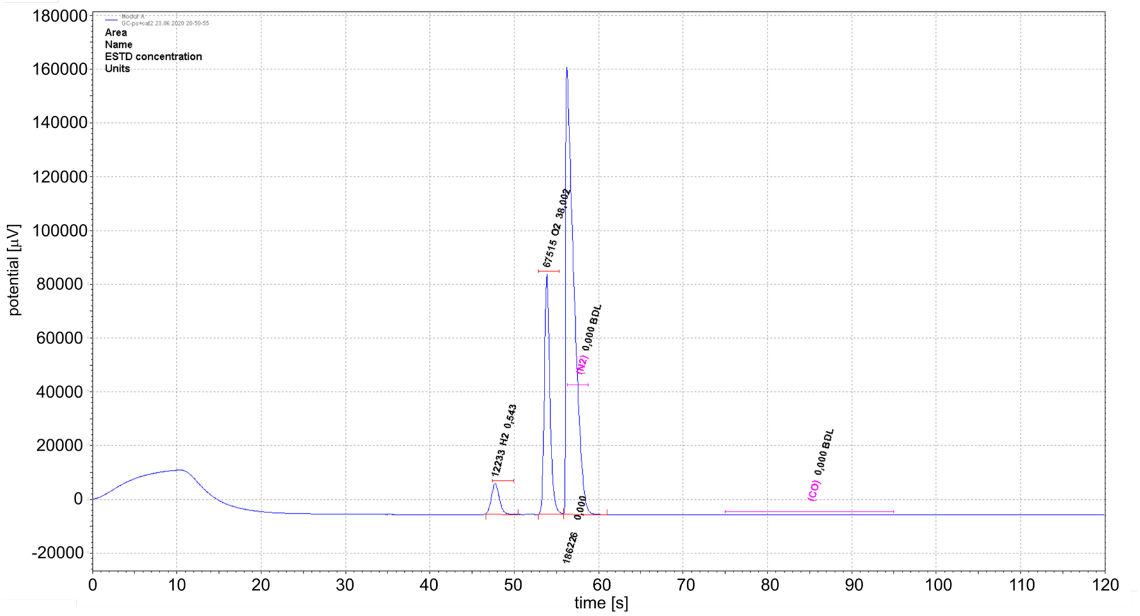

Figure S33: Hydrogen detection of [Fe-BL]+[Co] measurement.

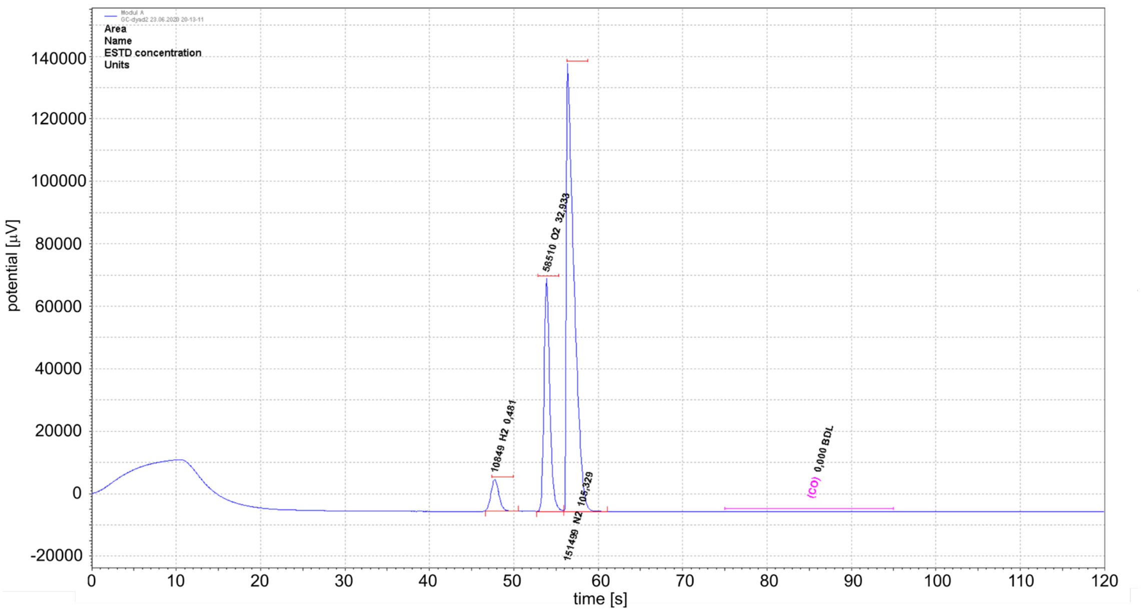

Figure S34: Hydrogen detection of [Fe-BL-Co] measurement.

9. Spectra

4,4'-bpy-Cl$_2$

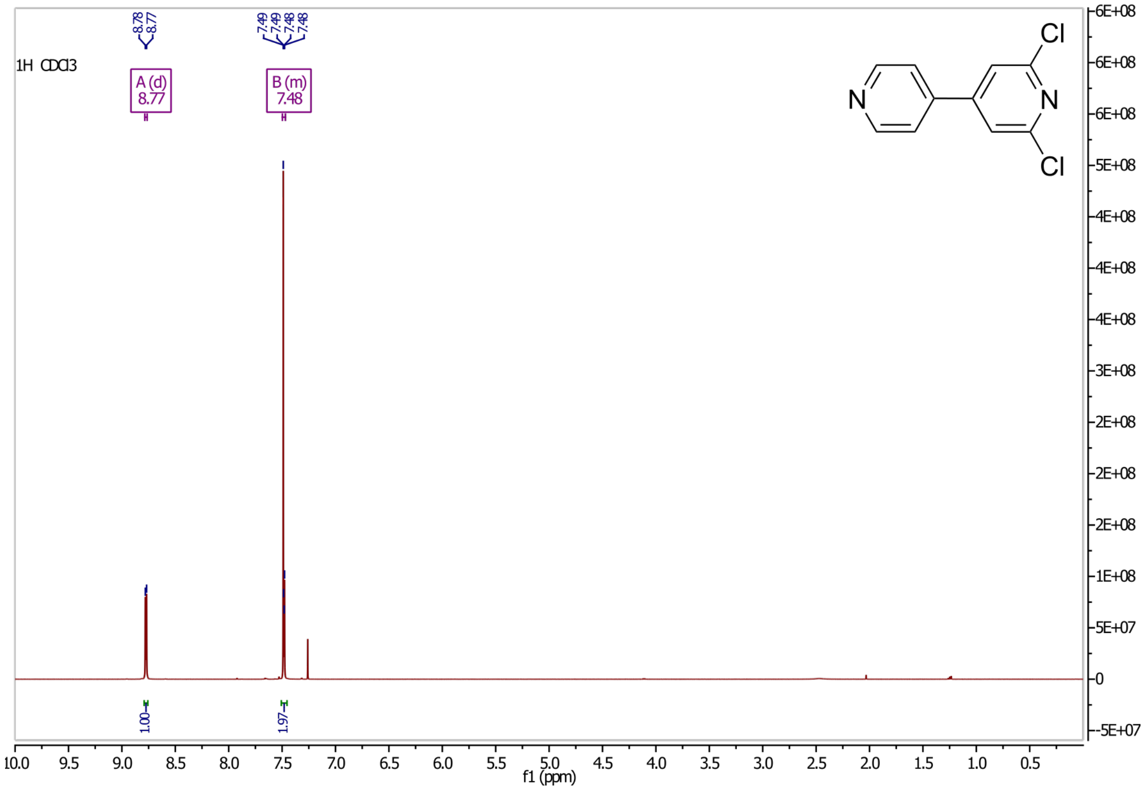

Figure S35: ^{1}H-NMR of 4,4'-bpy-Cl$_2$ in CDCl$_3$ (500 MHz, 300 K).

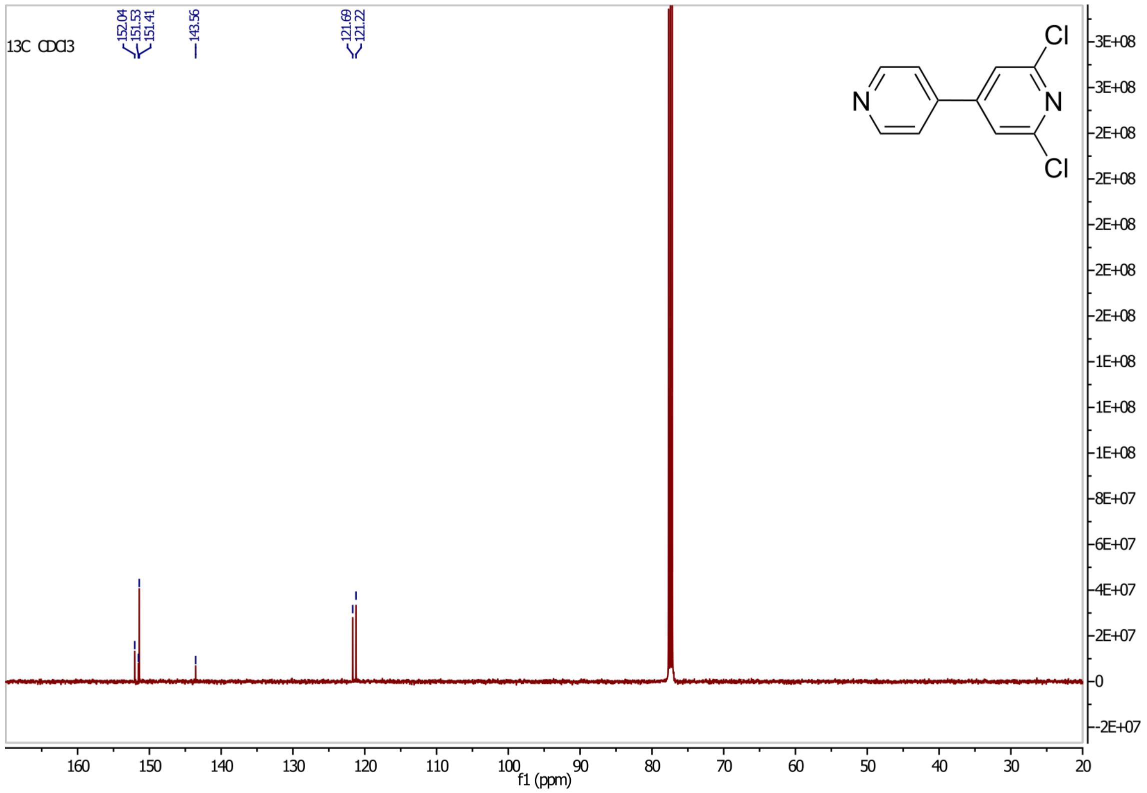

Figure S36: ^{13}C-NMR of 4,4'-bpy-Cl$_2$ in CDCl$_3$ (126 Hz, 300 K).

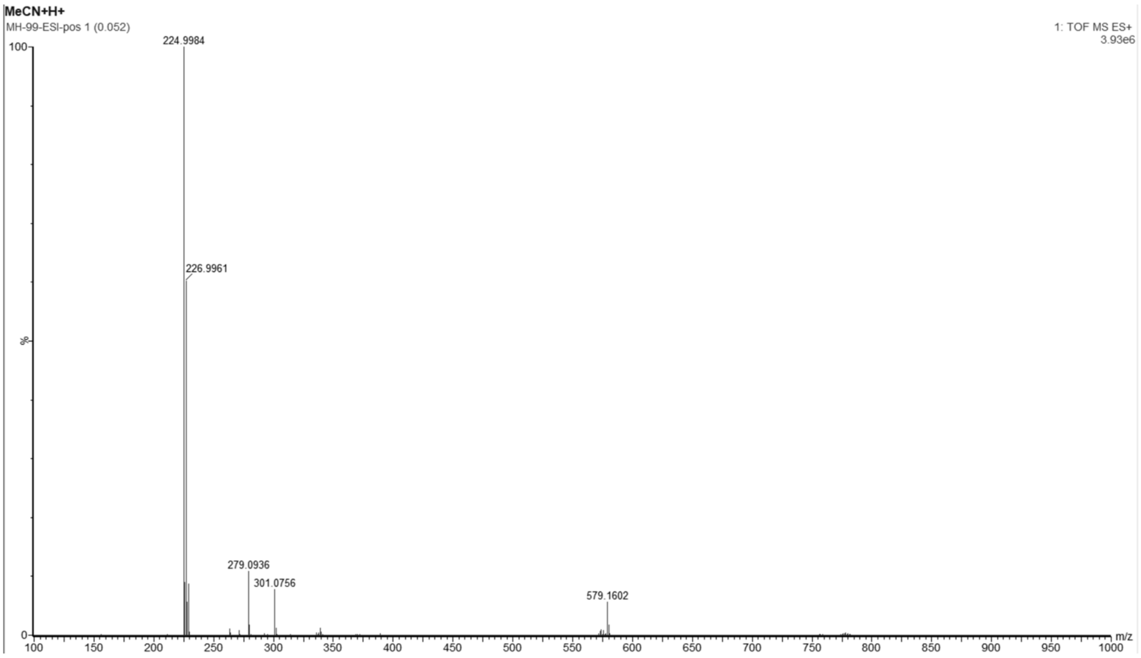

Figure S37: ESI-MS of 4,4'-bpy-Cl_2.

BL-Cl_2

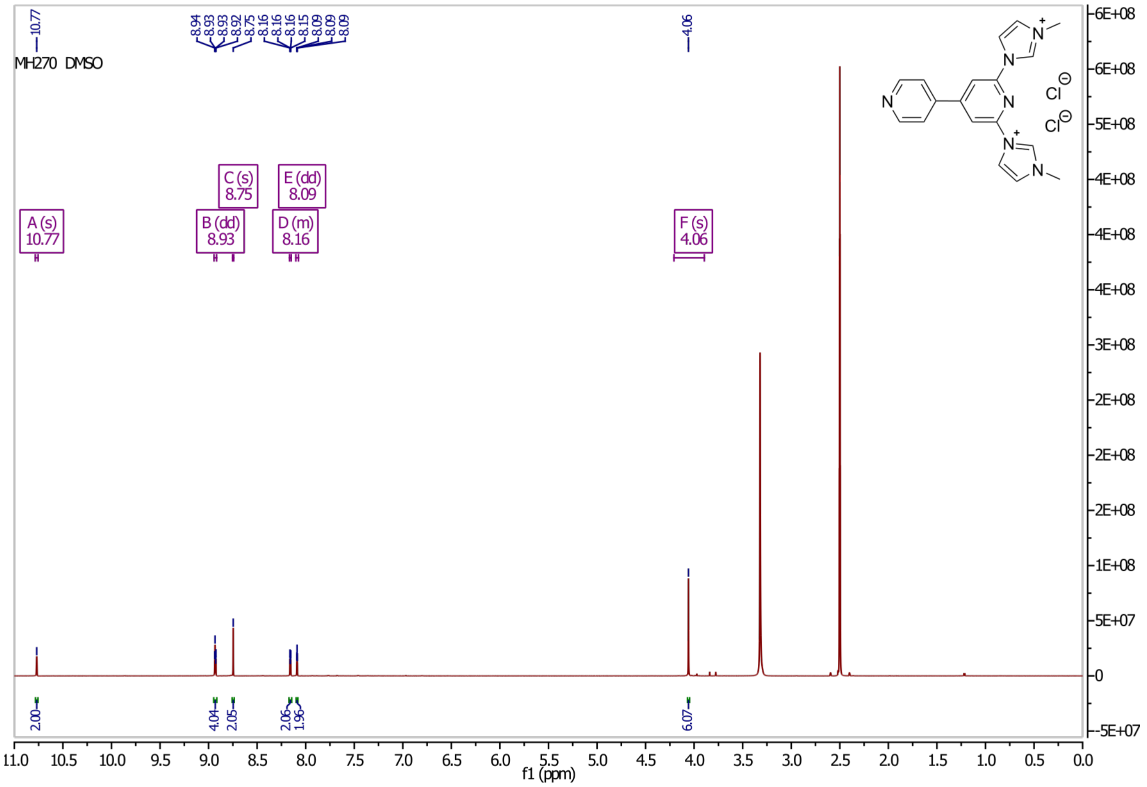

Figure S38: ^{1}H-NMR of BL-Cl_2 in DMSO-d_6 (500 MHz, 300 K).

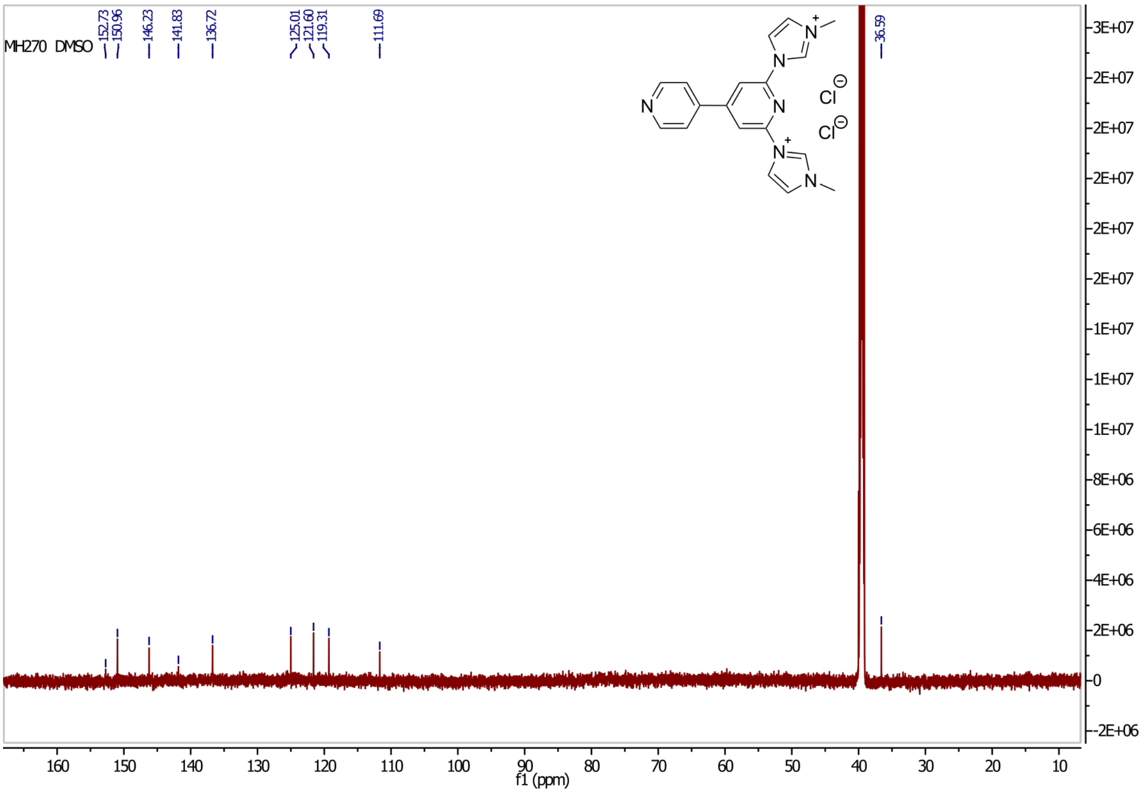

Figure S39: ^{13}C-NMR of BL-Cl_2 in DMSO-d_6 (126 MHz, 300 K).

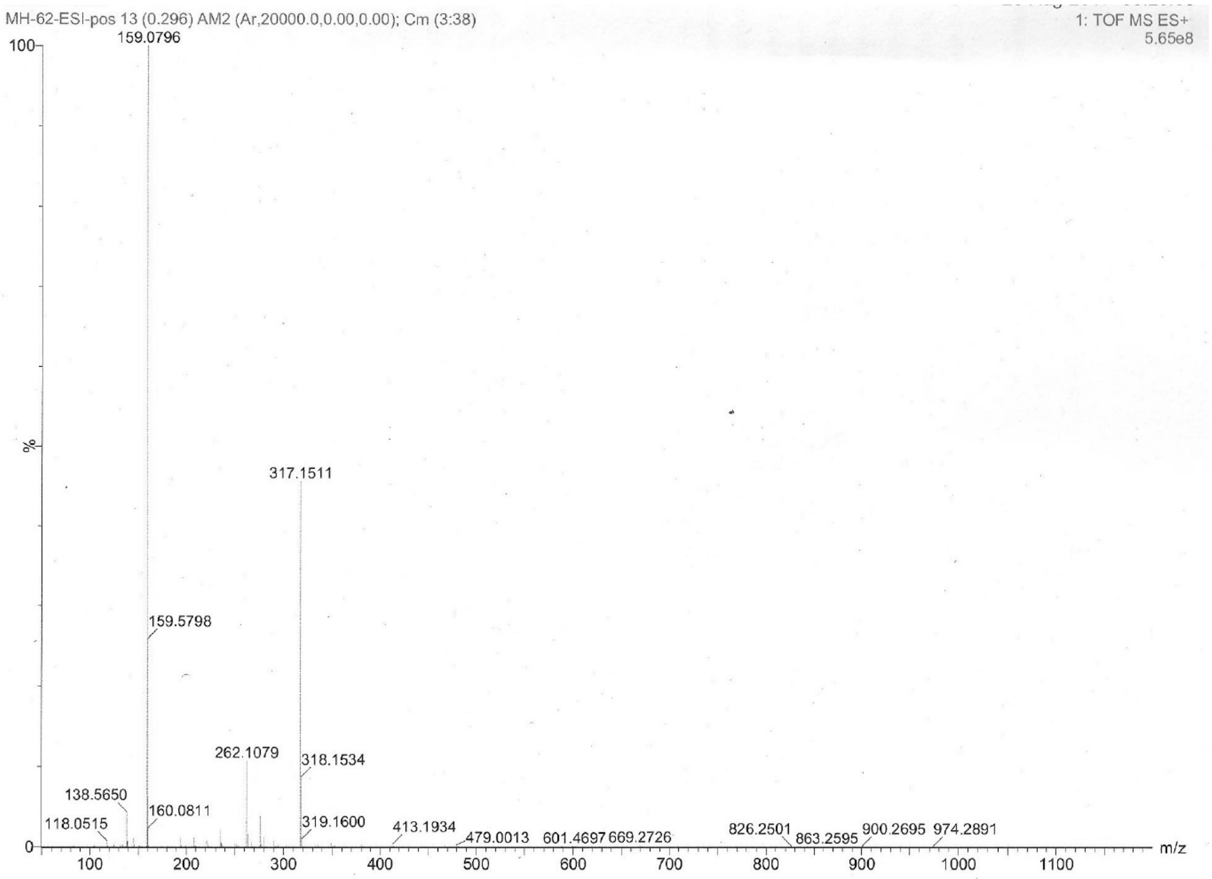

Figure S40: ESI-MS of BL-Cl_2.

[Fe-BL]

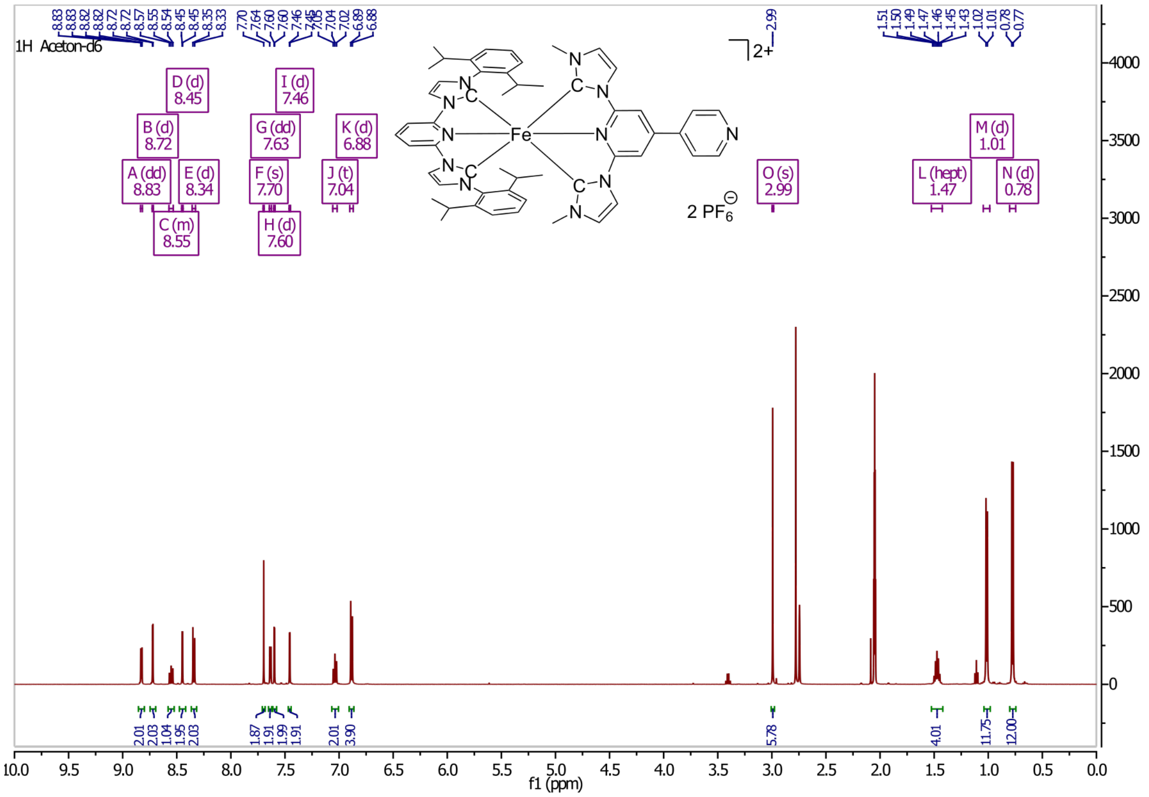

Figure S41: ^{1}H-NMR of [Fe-BL] in acetone-d_6 (500 MHz, 300 K).

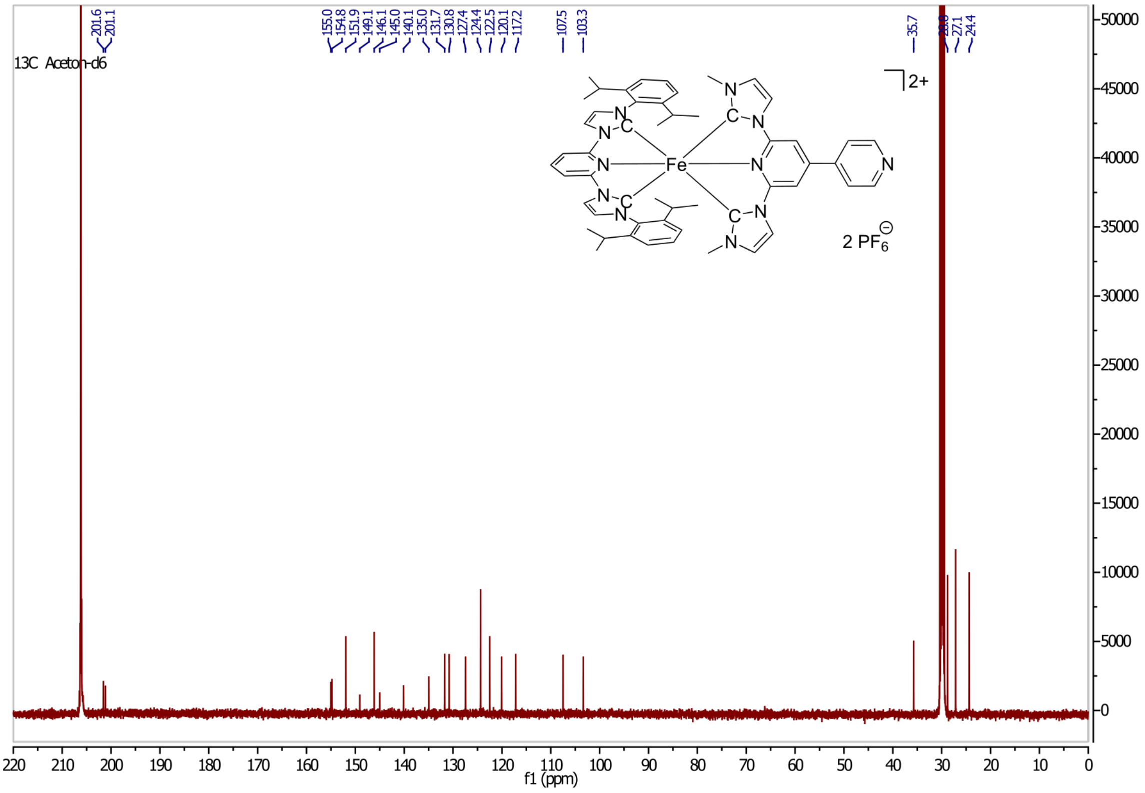

Figure S42: ^{13}C-NMR of [Fe-BL] in acetone-d_6 (126 MHz, 300 K).

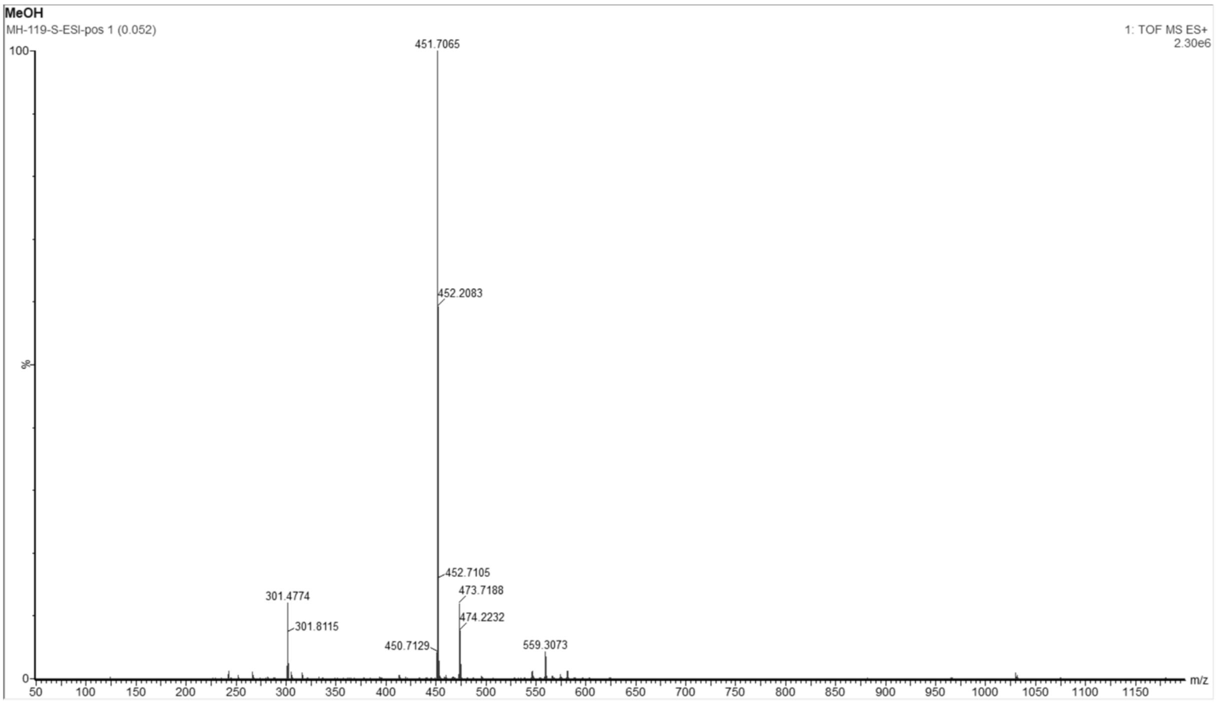

Figure S43: ESI-MS of [Fe-BL].

[Fe-BL-Co]

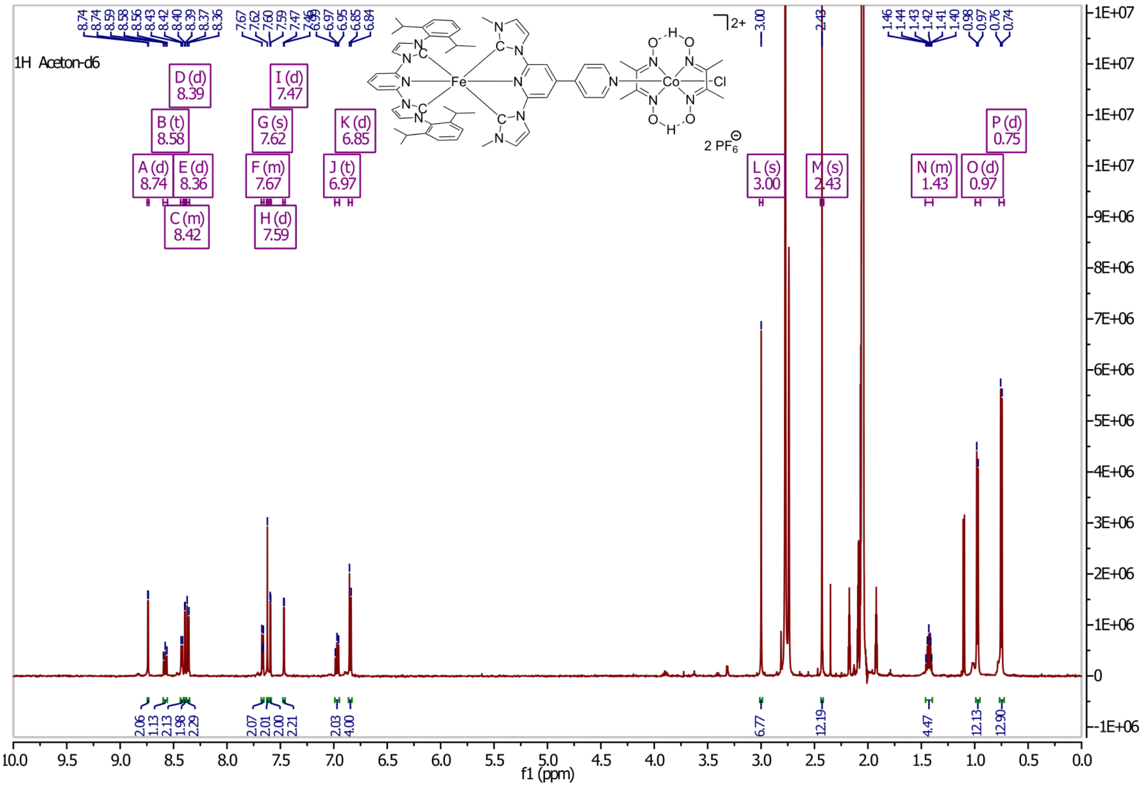

Figure S44: ^{1}H-NMR of [Fe-BL-Co] in acetone-d$_6$ (700 MHz, 298 K).

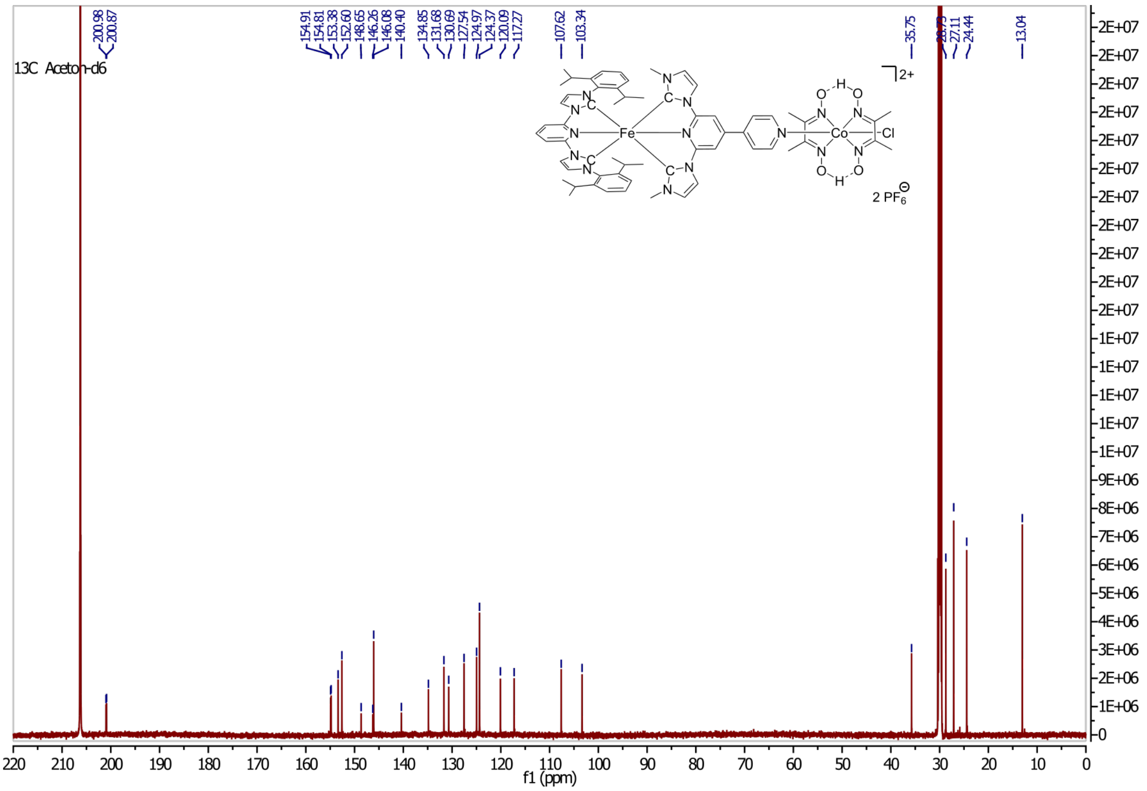

Figure S45: ^{13}C-NMR of [Fe-BL-Co] in acetone-d_6 (176 MHz, 298 K).

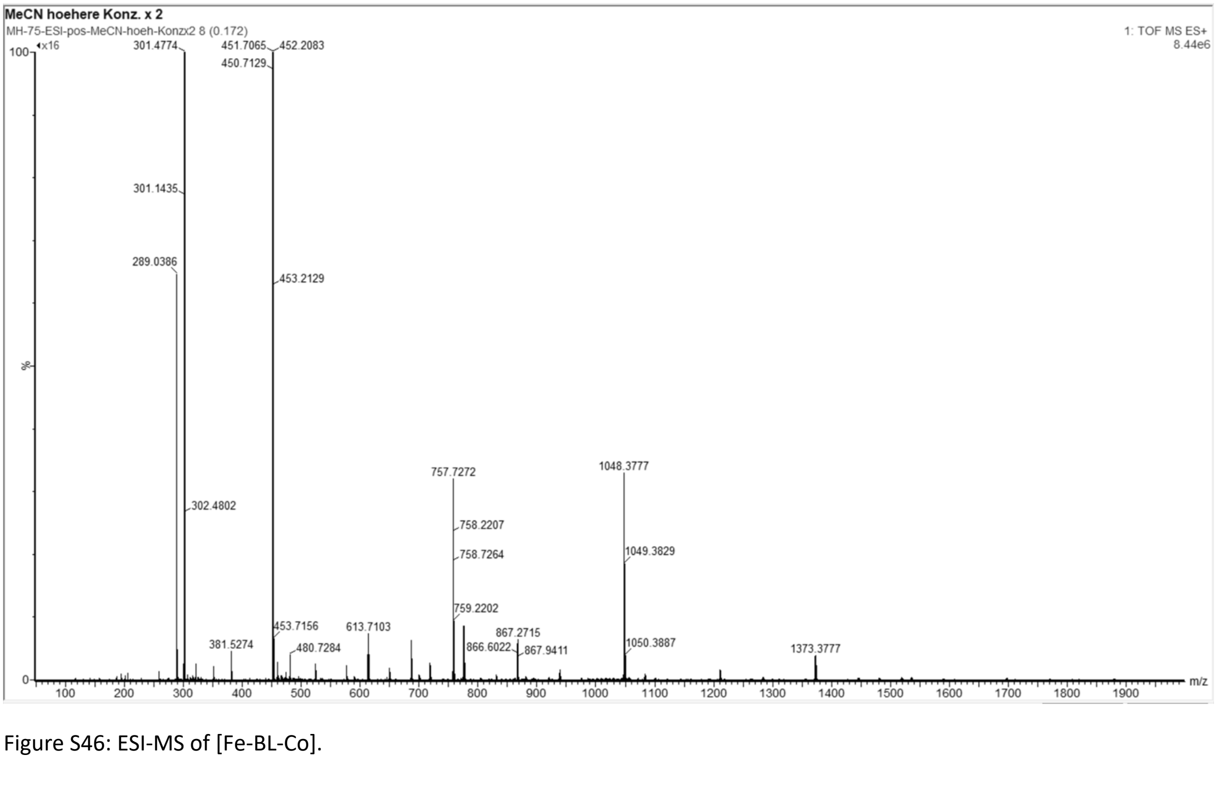

Figure S46: ESI-MS of [Fe-BL-Co].

References

(1) Ravel, B.; Newville, M. ATHENA, ARTEMIS, HEPHAESTUS: data analysis for X-ray absorption spectroscopy using IFEFFIT. *J. Synchrotron Rad.* **2005**, *12* (Pt 4), 537–541. DOI: 10.1107/S0909049505012719.

(2) Rehr, J. J.; Kas, J. J.; Prange, M. P.; Sorini, A. P.; Takimoto, Y.; Vila, F. Ab initio theory and calculations of X-ray spectra. *C. R. Physique* **2009**, *10* (6), 548–559. DOI: 10.1016/j.crhy.2008.08.004.

(3) Rehr, J. J.; Kas, J. J.; Vila, F. D.; Prange, M. P.; Jorissen, K. Parameter-free calculations of X-ray spectra with FEFF9. *Phys. Chem. Chem. Phys.* **2010**, *12* (21), 5503–5513. DOI: 10.1039/B926434E.

(4) Nowakowski, M.; Czapla-Masztafiak, J.; Szlachetko, J.; Kwiatek, W. M. Electronic structure of Fe, α-Fe 2 O 3 and Fe(NO 3) 3 × 9 H 2 O determined using RXES. *Chemical Physics* **2017**, *493*, 49–55. DOI: 10.1016/j.chemphys.2017.06.002.

(5) Stöhr, J. *Analysis of K-Shell Excitation Spectra by Curve Fitting. In: NEXAFS Spectroscopy. Springer Series in Surfaces Sciences*, Vol. 25; Springer Berlin Heidelberg, 1992. DOI: 10.1007/978-3-662-02853-7_7.

(6) Zimmer, P.; Burkhardt, L.; Friedrich, A.; Steube, J.; Neuba, A.; Schepper, R.; Müller, P.; Flörke, U.; Huber, M.; Lochbrunner, S.; Bauer, M. The Connection between NHC Ligand Count and Photophysical Properties in Fe(II) Photosensitizers:An Experimental Study. *Inorg. Chem.* **2018**, *57* (1), 360–373. DOI: 10.1021/acs.inorgchem.7b02624.

(7) Zimmer, P.; Burkhardt, L.; Schepper, R.; Zheng, K.; Gosztola, D.; Neuba, A.; Flörke, U.; Wölper, C.; Schoch, R.; Gawelda, W.; Canton, S. E.; Bauer, M. Towards Noble-Metal-Free Dyads:Ground and Excited State Tuning by a Cobalt Dimethylglyoxime Motif Connected to an Iron N-Heterocyclic Carbene Photosensitizer. *Eur. J. Inorg. Chem.* **2018**, *2018* (48), 5203–5214. DOI: 10.1002/ejic.201800946.

(8) Yamamoto, T. Assignment of pre-edge peaks in K-edge x-ray absorption spectra of 3d transition metal compounds: electric dipole or quadrupole? *X-Ray Spectrom.* **2008**, *37* (6), 572–584. DOI: 10.1002/xrs.1103.

(9) Groot, F. M. F. de; Glatzel, P.; Bergmann, U.; van Aken, P. A.; Barrea, R. A.; Klemme, S.; Hävecker, M.; Knop-Gericke, A.; Heijboer, W. M.; Weckhuysen, B. M. 1s2p resonant inelastic X-ray scattering of iron oxides. *J. Phys. Chem. B* **2005**, *109* (44), 20751–20762. DOI: 10.1021/jp054006s.

(10) Szlachetko, J.; MICHALOW-MAUKE, K.; Nachtegaal, M.; SÁ, J. Determination of conduction and valence band electronic structure of anatase and rutile TiO 2. *J Chem Sci* **2014**, *126* (2), 511–515. DOI: 10.1007/s12039-014-0584-1.

(11) Newville, M.; Kas, J. J.; Rehr, J. J. Improvements in modeling EXAFS with many-pole self-energy and FEFF 8.5. *J. Phys.: Conf. Ser.* **2009**, *190*, 12023. DOI: 10.1088/1742-6596/190/1/012023.

(12) Geremia, S.; Dreos, R.; Randaccio, L.; Tauzher, G.; Antolini, L. Evidence of the interaction between steric and electronic influence in rhodoximes and cobaloximes. Synthesis of pyRh(DH)2I and X-ray structure of pyRh(DH)2Cl, pyCo(DH)2Cl and pyRh(DH)2I. *Inorganica Chimica Acta* **1994**, *216* (1-2), 125–129. DOI: 10.1016/0020-1693(93)03708-I.

(13) Zimmer, P.; Müller, P.; Burkhardt, L.; Schepper, R.; Neuba, A.; Steube, J.; Dietrich, F.; Flörke, U.; Mangold, S.; Gerhards, M.; Bauer, M. N-Heterocyclic Carbene Complexes of Iron as

Photosensitizers for Light-Induced Water Reduction. *Eur. J. Inorg. Chem.* **2017**, *2017* (11), 1504–1509. DOI: 10.1002/ejic.201700064.
